# Transfer Learning for Moderate–Dimensional Ridge-Regularized Robust Linear Regression

**DOI:** 10.3390/e28050543

**Published:** 2026-05-11

**Authors:** Lingfeng Lyu, Xiao Guo, Zongqi Liu

**Affiliations:** Department of Statistics and Finance, School of Management, University of Science and Technology of China, Hefei 230026, China; lief1997@mail.ustc.edu.cn (L.L.); lzq20030713@mail.ustc.edu.cn (Z.L.)

**Keywords:** moderate dimension, non-sparse, robust regression, transfer learning

## Abstract

This paper studies transfer learning for ridge-regularized robust linear regression in the moderate–dimensional regime, where the number of predictors is of the same order as the sample size and the regression coefficients are not assumed to be sparse. We propose Trans-RR, which combines a robust ridge estimator from a source study with a robust ridge correction based on the target study. Under mild assumptions, we characterize the asymptotic estimation error of the proposed estimator and show that leveraging source data can substantially improve estimation accuracy relative to the traditional single-study ridge-regularized robust estimator. To guard against negative transfer when the source study is not sufficiently informative, we further propose an adaptive aggregation of Trans-RR with the single-task estimator that selects the mixing weight by cross-validation. Simulation studies and a real-data analysis support the theory and illustrate the transition between positive and negative transfer as the discrepancy between the source and target studies varies.

## 1. Introduction

Modern statistical analyses often involve several related datasets collected from different studies, populations, or experiments. A basic question is how to use these datasets together to improve prediction and estimation for a study of interest. Transfer learning addresses this question by borrowing useful information from related tasks. It is now standard in machine learning and has been successful in applications such as natural language processing, remote sensing, and computer vision [1,2,3]. In statistics, it has also become an important tool for improving performance in multi-study problems.

In many contemporary applications, regression problems arise in a moderate–dimensional regime, where the number of predictors is of the same order as the sample size and sparsity is often not a reasonable structural assumption. At the same time, heavy-tailed errors or outlying observations may substantially affect estimation accuracy. This setting arises naturally in multisite metabolomics studies, where many metabolites are measured simultaneously, between-cohort heterogeneity is often present, and outliers or other data contamination can be an important practical concern [4]. These features make transfer learning particularly challenging: related source studies may contain useful information, but effective borrowing requires methods that are robust to contamination and suitable for moderate–dimensional, non-sparse settings.

Existing theoretical work on transfer learning covers several important settings. For linear regression, ref. [5] studied data-enriched regression in a fixed-dimensional setting, and ref. [6] analyzed linear models with a shared low-dimensional representation across tasks. In the high-dimensional sparse regime, ref. [7] considered transfer learning with proxy data, while ref. [8] established prediction and estimation guarantees for sparse linear regression. For high-dimensional generalized linear models, refs. [9,10] developed transfer learning methods with theoretical guarantees. Transfer learning has also been studied for nonparametric classification [11], nonparametric regression [12], and settings with unreliable source data [13]. However, these works do not apply to the moderate–dimensional robust setting considered here.

Robust regression has, in contrast, been extensively studied in the single-study setting. For classical M-estimation, a substantial body of work has established asymptotic results when p/n→0 while p→∞, including [14,15,16,17,18,19]. When p/n→κ∈(0,1), robust regression has a qualitatively different asymptotic behavior [20]. When p/n→κ>0, ref. [21] proposed a ridge-regularized robust estimator to address the nonexistence of the ordinary robust estimator. However, these results do not directly extend to the transfer learning framework when related source data are available.

Motivated by these gaps, we study transfer learning under a moderate–dimensional robust linear model with one target study and one related source study. We allow the predictor dimension to be of the same order as the target and source sample sizes, do not impose sparsity assumptions on the regression coefficients, and permit heavy-tailed errors. Within this framework, our goal is to leverage information from the source study to improve the estimation performance of traditional single-task approaches.

Our main contribution is to develop and analyze a transfer learning method for moderate–dimensional ridge-regularized robust linear regression. First, we propose Trans-RR, a transfer learning procedure for ridge-regularized robust linear regression. It combines a robust ridge fit on the source study with a robust ridge correction on the target study and is designed for non-sparse coefficients. Second, we derive an asymptotic characterization of the $l_{2}$ risk of the resulting estimator under mild assumptions on the design and error distributions. The theory shows how auxiliary source data can improve estimation accuracy relative to the single-study ridge-regularized robust estimator, while also clarifying the possibility of negative transfer. Third, we propose an adaptive aggregation of Trans-RR with the single-task estimator that selects the mixing weight by cross-validation, providing a data-driven safeguard against negative transfer. Fourth, we conduct simulation studies and a real-data analysis to examine the practical performance of the proposed methods, including their sensitivity to tuning choices and to the identity–covariance assumption underlying the theory.

The rest of the paper is organized as follows. Section 2 introduces the model setup and the proposed algorithm. Section 3 presents the technical assumptions, the theoretical results, and the adaptive aggregation against negative transfer. Section 4 presents simulation studies to evaluate the performance of the proposed methods. Section 5 applies the proposed methods to a real-data example. The proofs of the main theoretical result as well as the lemmas are included in Appendix D and Appendix E.

Notation. Denote by $I_{m}$ the m×m identity matrix. Let $0_{m}\in R^{m}$ and $1_{m}\in R^{m}$ be the vectors of zeros and ones, respectively. For a vector $v={\left(v_{1},\ldots ,v_{m}\right)}^{⊤}$, the $l_{2}$ norms are $∥v∥={\left(\sum_{i=1}^{m}v_{i}^{2}\right)}^{1/2}$, whereas ${∥v∥}_{\infty }=\max_{1\leq k\leq p}\left|v_{k}\right|$. For an m×m matrix $A={\left\{a_{ij}\right\}}_{1\leq i,j\leq m}$, denote by $\lambda_{\max}\left(A\right)$ and $\lambda_{\min}\left(A\right)$ the maximum and minimum eigenvalues of A, respectively. The $L_{2}$ norm of A is defined as $∥A∥={\left\{\lambda_{\max}\left(A^{⊤}A\right)\right\}}^{1/2}$.

## 2. Methodology

### 2.1. Problem Setup

We consider a transfer learning problem with one target study and one related source study. In the target study, we observe *n* samples $x_{i}\in R^{p}$ and $y_{i}\in R$, i=1,…,n, generated from

(1) $$y_{i}=x_{i}^{⊤}\beta_{0}+\epsilon_{i},$$

where $\epsilon_{i},i=1,\ldots ,n$, are independently distributed errors and $\beta_{0}\in R^{p}$ is the unknown regression parameter of interest.

In addition to the target data, we observe $n_{1}$ samples $\left(x_{i}^{\left(1\right)},y_{i}^{\left(1\right)}\right)$, $i=1,\ldots ,n_{1}$, from the source study satisfying

(2) $$y_{i}^{\left(1\right)}={\left(x_{i}^{\left(1\right)}\right)}^{⊤}w_{0}+\epsilon_{i}^{\left(1\right)},$$

where $w_{0}\in R^{p}$ is the regression parameter for the source study and $\epsilon_{i}^{\left(1\right)},i=1,\ldots ,n_{1}$, are independently distributed errors. Throughout, both $\epsilon_{i}$ and $\epsilon_{i}^{\left(1\right)}$ are allowed to be heavy-tailed.

We work in the moderate–dimensional regime, where *p* is of the same order as both *n* and $n_{1}$, with p/n→κ∈(0,∞) and $p/n_{1}\to \kappa_{1}\in \left(0,\infty \right)$. We also do not impose sparsity assumptions on $\beta_{0}$ or $w_{0}$. Let $\delta_{0}=\beta_{0}-w_{0}$ denote the source–target discrepancy and let $h=∥\delta_{0}∥$ measure its size. Smaller values of *h* correspond to a more informative source study and hence a greater potential for useful transfer.

### 2.2. Trans-RR Algorithm

Based on this setup, we now introduce the proposed transfer learning algorithm, referred to as Trans-RR. Following the general two-stage strategy used in [7,8,9], the core of the procedure consists of two estimation steps. The first step estimates the source coefficient vector $w_{0}$ from the source data. The second step estimates the source–target discrepancy $\delta_{0}=\beta_{0}-w_{0}$ from the target data, after which the two estimates are combined. Algorithm 1 summarizes the procedure.    
**Algorithm 1:** Trans-RR algorithm
 **Input:** target data ${\left\{\left(x_{i},y_{i}\right)\right\}}_{i=1}^{n}$ and source data ${\left\{\left(x_{i}^{\left(1\right)},y_{i}^{\left(1\right)}\right)\right\}}_{i=1}^{n_{1}}$
 **Output:** the estimated coefficient vector $\hat{\beta }$
 Step 1. Compute
(3)$$\hat{w}=\underset{w\in R^{p}}{\mathrm{arg min}}\left[\frac{1}{n_{1}}\sum_{i=1}^{n_{1}}\tilde{\rho }\left\{y_{i}^{\left(1\right)}-{\left(x_{i}^{\left(1\right)}\right)}^{⊤}w\right\}+\frac{\tau_{1}}{2}{∥w∥}^{2}\right]$$

 for some constant $\tau_{1}$.

 Step 2. Compute
(4)$$\hat{\delta }=\underset{\delta \in R^{p}}{\mathrm{arg min}}\left[\frac{1}{n}\sum_{i=1}^{n}\rho \left\{y_{i}-x_{i}^{⊤}\left(\hat{w}+\delta \right)\right\}+\frac{\tau }{2}{∥\delta ∥}^{2}\right]$$
 for some constant τ.

 Step 3. Let
(5)$$\hat{\beta }=\hat{w}+\hat{\delta }.$$

 Step 4. Output $\hat{\beta }$.

The idea behind the construction is straightforward. Step 1 computes a ridge-regularized robust estimator from the source study. Step 2 then estimates the discrepancy relative to the source-stage fit by solving a second ridge-regularized robust regression problem on the target study. The final estimator is obtained by combining these two pieces, namely $\hat{\beta }=\hat{w}+\hat{\delta }$. The main difference between our procedure and those of [7,8,9] is that we use ridge/$l_{2}$ regularization in both steps, whereas they use $l_{1}$ regularization. This choice is motivated by our diffuse-coefficient setting: the regression parameters $\beta_{0}$ and $w_{0}$ have many small coordinates and are not well approximated by sparse vectors. In this setting, lasso-based methods are not well-suited to the problem, whereas ridge penalization is natural. Another difference is that we use robust loss functions rather than the quadratic loss, which makes the procedure less sensitive to outliers and heavy-tailed errors.

**Remark 1.** 
*When robustness to heavy-tailed errors or outliers is needed, Huber-type loss functions are natural candidates for ρ and $\tilde{\rho }$. Specific choices of ρ and $\tilde{\rho }$ under our theoretical framework are discussed in Section 3. The regularization parameters τ and $\tau_{1}$ may be selected by standard data-driven tuning methods such as cross-validation.*

## 3. Theoretical Results

This section introduces the assumptions for the analysis and then presents the main asymptotic error results for Trans-RR.

### 3.1. Technical Assumptions

We study the estimation error of the estimator in Algorithm 1 under the following assumptions. We state the assumptions separately for the target study (Assumption 1) and the source study (Assumption 2), since the two stages are based on different samples. The two sets of conditions are largely parallel.

**Assumption 1.** 

*(a)*  *p/n→κ∈(0,∞).*
*(b)*  *Suppose ρ is an even and convex function. Assume that $\psi =\rho^{\prime }$ is bounded and $\psi^{\prime }$ is Lipschitz and bounded. Moreover, we assume that sign(ψ(x))=sign(x) and that ρ(x)≥ρ(0)=0 for all x∈R.*
*(c)*  *Assume that there exist independent variables $\lambda_{i}$’s and $X_{i}$’s such that $x_{i}=\lambda_{i}X_{i}$. Suppose that $X_{i}$’s are i.i.d. with independent entries, and they have mean $0_{p}$ and $\mathrm{cov}\left(X_{i}\right)=I_{p}$. Suppose there exist $c_{n}$ and $C_{n}$ that vary with n, where $1/c_{n}=O\left(\mathrm{polyLog}\left(n\right)\right)$ and $C_{n}$ is bounded in n, such that for any convex 1-Lipschitz function G of $X_{i}$, $P(|G\left(X_{i}\right)-m_{G}\left|>t\right)\leq C_{n}\exp\left(-c_{n}t^{2}\right)$ holds for all t>0, where $m_{G}$ is the median of $G(X_{i})$. We require the same assumption to hold for the columns of the n×p design matrix X. Additionally, we assume that the coordinates of $X_{i}$ have moments of all orders, and the k-th moment of the entries of $X_{i}$ is assumed to be uniformly bounded independently of n and p for all k.*
*(d)*  *Suppose that $\lambda_{i}$’s are independent, with $E(\lambda_{i}^{2})=1$, $E(\lambda_{i}^{4})$ being bounded, and $\sup_{1\leq i\leq n}\left|\lambda_{i}\right|$ growing at most like $C_{\lambda }{\left(\log n\right)}^{k}$ for some k. $\lambda_{i}$’s may have finitely many possible distributions.*
*(e)*  *Suppose that $\epsilon_{i}$’s are independent and are also independent of $X_{i}$’s and $\lambda_{i}$’s. They may have finitely many possible distributions, each with a density that is differentiable, symmetric, and unimodal. If $f_{i}$ is the density of one such distribution, we assume that $\lim_{x\to \infty }xf_{i}\left(x\right)=0$.*
*(f)*  *The fraction of occurrences for each possible combination of distributions for $\left(\epsilon_{i},\lambda_{i}\right)$ has a limit as n→∞.*
*(g)*  *There exist constants $C_{\beta }$ and e>1/3 such that $∥\beta_{0}∥\;\leq \;C_{\beta }$ and $∥\beta_{0}{∥}_{\infty }\;=\;O\left(n^{-e}\right)$.*

**Assumption 2.** 

*(a)*  *$p/n_{1}\to \kappa_{1}\in \left(0,\infty \right)$.*
*(b)*  *$\tilde{\rho }$ and $\tilde{\psi }$ satisfy Assumption 1(b).*
*(c)*  *$x_{i}^{\left(1\right)}$, $X_{i}^{\left(1\right)}$’s and $\lambda_{i}^{\left(1\right)}$’s satisfy Assumption 1(c).*
*(d)*  *$\lambda_{i}^{\left(1\right)}$’s satisfy Assumption 1(d).*
*(e)*  *$\epsilon_{i}^{\left(1\right)}$’s, $X_{i}^{\left(1\right)}$’s and $\lambda_{i}^{\left(1\right)}$’s satisfy Assumption 1(e).*
*(f)*  *$\lambda_{i}^{\left(1\right)}$’s and $\epsilon_{i}^{\left(1\right)}$’s satisfy Assumption 1(f).*
*(g)*  *$∥w_{0}{∥}_{2}$ remains bounded. Furthermore, $∥w_{0}{∥}_{\infty }=O\left(n_{1}^{-e}\right)$, where e>1/3.*

For Assumptions 1(b) and 2(b), it is quite common in robust statistics to require ψ to be bounded. For example, the Huber loss

$$\rho_{H}\left(x\right)=\{\begin{array}{cc} \frac{x^{2}}{2} & \;\mathrm{if}\;|x|\leq \delta ,\\ \delta \cdot \left(\left|x\right|-\frac{1}{2}\delta \right) & \;\mathrm{otherwise}. \end{array}$$

is chosen to grow linearly to infinity, which reduces the influence of outliers on the resulting regression estimator. Although the Huber loss does not fully satisfy the assumptions because it is not differentiable at |x|=δ, these assumptions hold for a smoothed approximation such as

(6) $$\rho_{\eta }\left(x\right)=\{\begin{array}{cc} \frac{x^{2}}{2} & \mathrm{if}|x|\leq \delta -\eta ,\\ \left(\delta -\frac{\eta }{2}\right)\cdot \left|x\right|+\frac{{\left(\delta -|x|\right)}^{3}}{6\eta }+C_{\rho } & \mathrm{if}|x|\in (\delta -\eta ,\delta ),\\ \left(\delta -\frac{\eta }{2}\right)\cdot \left|x\right|+C_{\rho } & \mathrm{if}|x|\geq \delta , \end{array}$$

where $C_{\rho }=-\eta^{2}/6+\eta \delta /2-\delta^{2}/2$. The corresponding $\psi_{\eta }$ is given by

(7) $$\psi_{\eta }\left(x\right)=\{\begin{array}{cc} x & \mathrm{if}|x|\leq \delta -\eta ,\\ \mathrm{sign}\left(x\right)\cdot \left\{\delta -\frac{\eta }{2}-\frac{{\left(\delta -|x|\right)}^{2}}{2\eta }\right\} & \mathrm{if}|x|\in (\delta -\eta ,\delta ),\\ \mathrm{sign}\left(x\right)\cdot \left(\delta -\frac{\eta }{2}\right) & \mathrm{if}|x|\geq \delta . \end{array}$$

Another example that fully satisfies the assumptions is the pseudo-Huber loss function from [22,23], defined by

(8) $$\rho_{P}\left(x\right)=\delta^{2}\left(\sqrt{1+\frac{x^{2}}{\delta^{2}}}-1\right).$$

The assumptions on $x_{i}$’s and $x_{i}^{\left(1\right)}$’s, in particular that they have mean $0_{p}$ and covariance matrix $I_{p}$, are common in the study of M-estimators for linear models. These assumptions have been used in the low-dimensional regime p/n→0 studied in [14,15,24], in the moderate–dimensional regime p/n→κ∈(0,1) considered in [20], and in the regime p/n→κ>0 analyzed in [21,25].

The concentration assumption on $X_{i}$’s and $X_{i}^{\left(1\right)}$’s is weaker than the Gaussian assumptions often imposed in robust statistics. This assumption has also been studied in [21,26] and holds for a broad class of distributions. Corollary 4.10 in [27] demonstrates that our assumptions are satisfied if $X_{i}$ has independent entries bounded by $1/(2\sqrt{c_{1}})$ for some $c_{1}>0$. Additionally, Theorem 2.7 in [27] shows that the assumptions hold when $X_{i}$ has independent entries with density $f_{k}$, 1≤k≤p, such that $f_{k}\left(x\right)=\exp\left(-u_{k}\left(x\right)\right)$ and $u_{k}^{\prime \prime }\left(x\right)\geq \sqrt{c_{2}}$ for some $c_{2}>0$. In particular, this condition holds when $X_{i}$ has i.i.d. N(0,1) entries, where $c_{2}=1$. As will be seen in the proof, the functions *G* that arise in our analysis are either linear functions or square roots of quadratic forms. A similar discussion applies to the $X_{i}^{\left(1\right)}$’s.

The introduction of $\lambda_{i}$ and $\lambda_{i}^{\left(1\right)}$, as also considered in [20,21], is used to induce a nonspherical geometry on the predictors. Although the assumption $E(\lambda_{i}^{2})=1$ can be relaxed to the requirement that $E(\lambda_{i}^{2})$ be uniformly bounded, it remains statistically important because it guarantees that $\mathrm{cov}\left(x_{i}\right)=I_{p}$ in all the models we consider. This construction shows that many models can share the same covariance $\mathrm{cov}(x_{i})$ while exhibiting substantially different estimation errors for $\hat{\beta }$. This contrasts with the low-dimensional setting studied in [28], where $\mathrm{cov}(x_{i})$ is the key quantity for robust regression. A similar discussion applies to $\lambda_{i}^{\left(1\right)}$’s and $x_{i}^{\left(1\right)}$’s.

In Assumptions 1(e) and 2(e), no moment restriction is imposed on the $\epsilon_{i}$’s and $\epsilon_{i}^{\left(1\right)}$’s. For instance, smooth symmetric log-concave densities satisfy all of these assumptions; see [29,30]. Furthermore, the Cauchy distribution also satisfies these conditions; see Theorem 1.6 in [31]. This makes the framework compatible with heavy-tailed errors, which are of particular interest in robust regression.

Assumptions 1(g) and 2(g) impose a non-sparse structure on $\beta_{0}$ and $w_{0}$, meaning that these vectors cannot be well approximated by sparse vectors in $l_{2}$ norm. This setting is common in moderate–dimensional statistics and contrasts with the sparse regime, where only a small fraction of coefficients are substantial. Under these assumptions, the proposed Trans-RR estimator may outperform lasso-based methods.

We now turn to the target-stage result and the resulting error characterization for Trans-RR.

### 3.2. Asymptotic Characterization of Estimation Error

Our main theorem characterizes the asymptotic $l_{2}$ error of the Trans-RR estimator. Recall that $\hat{\beta }$ is defined in (5), and let τ>0 be fixed as *n* and *p* vary. To state the result, let prox(cρ) denote the proximal mapping of the function cρ, see [32]. It is given by

(9) $$\mathrm{prox}\left(c\rho \right)\left(x\right)=\underset{y\in R}{\mathrm{arg min}}\left\{c\;\rho \left(y\right)+\frac{1}{2}{\left(x-y\right)}^{2}\right\},\;x\in R.$$

When ρ is differentiable, prox(cρ)(x) is the unique y∈R satisfying y+cψ(y)=x, with $\psi =\rho^{\prime }$. prox(cρ)(x) can be viewed as a shrinkage of *x* toward the minimizer of ρ, with the amount of shrinkage depending on *c* and ρ. The proximal mapping is a standard object in convex analysis and convex optimization (see [33] for a review of its analytic properties and an introduction to proximal gradient algorithms). As explained in [34], the system in Theorem 1 can be reformulated in terms of $\mathrm{prox}({\left(c_{\rho }\left(\kappa \right)\rho \right)}^{*})$, where $f^{*}\left(u\right)=\sup_{y\in R}\left\{uy-f\left(y\right)\right\}$ denotes the Fenchel–Legendre conjugate of *f*.

Under Assumptions 1 and 2, the estimation error admits the following limit.

**Theorem 1.** 
*Under Assumptions 1 and 2, conditional on the source-stage estimator $\hat{w}$, which is independent of the target sample, we have $∥\hat{\beta }-\beta_{0}∥\to r_{\rho }\left(\kappa \right)$ in probability, where $r_{\rho }\left(\kappa \right)$ is deterministic for the given value of $\hat{w}$. Let $W_{i}=\epsilon_{i}+r_{\rho }\left(\kappa \right)\lambda_{i}Z_{i}$, where $Z_{i}$ is a standard normal random variable independent of $\epsilon_{i}$ and $\lambda_{i}$. Then there exists a constant $c_{\rho }\left(\kappa \right)$ such that*

(10) $$\{\begin{array}{cc} \lim_{n\to \infty }\frac{1}{n}\sum_{i=1}^{n}E\left\{{\left[\mathrm{prox}\left\{c_{\rho }\left(\kappa \right)\lambda_{i}^{2}\rho \right\}\right]}^{\prime }\left(W_{i}\right)\right\} & =1-\kappa +\tau c_{\rho }\left(\kappa \right),\\ \kappa \left[\lim_{n\to \infty }\frac{1}{n}\sum_{i=1}^{n}E\left(\frac{{\left[W_{i}-\mathrm{prox}\left\{c_{\rho }\left(\kappa \right)\lambda_{i}^{2}\rho \right\}\left(W_{i}\right)\right]}^{2}}{\lambda_{i}^{2}}\right)\right]+\tau^{2}{∥\beta_{0}-\hat{w}∥}^{2}c_{\rho }^{2}\left(\kappa \right) & =\kappa^{2}r_{\rho }^{2}\left(\kappa \right). \end{array}$$

The proof of Theorem 1 is given in Appendix E. Here and below, the dependence of $r_{\rho }\left(\kappa \right)$ and $c_{\rho }\left(\kappa \right)$ on $\hat{w}$ is suppressed for notational simplicity.

Theorem 1 shows that the asymptotic error $r_{\rho }\left(\kappa \right)$ depends on the source study only through the discrepancy $∥\beta_{0}-\hat{w}∥$. To investigate when positive transfer occurs, Section 4.2 numerically computes $r_{\rho }$ as a function of $∥\beta_{0}-\hat{w}∥$ under the smoothed Huber loss (6) in three simulation cases. The resulting curves are monotonically increasing across the displayed range. By the triangle inequality,

$$∥\beta_{0}-\hat{w}∥\leq ∥\beta_{0}-w_{0}∥+∥\hat{w}-w_{0}∥.$$

The right-hand side is the sum of the population gap between the source and target coefficients and the source-stage estimation error. Transfer is therefore expected to help when the source-stage estimator is accurate and close to the target coefficient, and to hurt when either the population gap $∥\beta_{0}-w_{0}∥$ or the source-stage estimation error $∥\hat{w}-w_{0}∥$ is large. In practice, Section 3.3 develops an adaptive Trans-RR estimator to avoid negative transfer.

Unlike several recent transfer learning analyses, such as [7,8,9], our theory does not impose direct structural restrictions on $\delta_{0}$, the difference between the target and source coefficients. Theorem 1 also shows that the performance of $\hat{\beta }$ depends on the distribution of the $\lambda_{i}$’s in the representation $x_{i}=\lambda_{i}X_{i}$ from Assumption 1(c). Thus, in the moderate–dimensional regime, the geometry of the target predictors encoded by $\lambda_{i}$ materially affects the estimation error. This again contrasts with low-dimensional robust regression, where $\mathrm{cov}(x_{i})$ is the dominant quantity.

When $\lambda_{i}^{2}=1$ for all *i* and the errors $\epsilon_{i}$ are i.i.d., Theorem 1 simplifies as follows.

**Corollary 1.** 
*Under the same assumptions as in Theorem 1, if $\lambda_{i}^{2}=1$ for all i and the errors $\epsilon_{i}$ are i.i.d., then, conditional on $\hat{w}$, we have $∥\hat{\beta }-\beta_{0}∥\to r_{\rho }\left(\kappa \right)$ in probability, where $r_{\rho }\left(\kappa \right)$ is deterministic for the given value of $\hat{w}$. Let $w=\epsilon +r_{\rho }\left(\kappa \right)z$, where ϵ has the same distribution as the $\epsilon_{i}$’s and z is a standard normal random variable independent of ϵ. Then there exists a constant $c_{\rho }\left(\kappa \right)$ such that*

$$\{\begin{array}{cc} E\left\{{\left[\mathrm{prox}\left\{c_{\rho }\left(\kappa \right)\rho \right\}\right]}^{\prime }\left(w\right)\right\} & =1-\kappa +\tau c_{\rho }\left(\kappa \right),\\ \kappa E\left({\left[w-\mathrm{prox}\left\{c_{\rho }\left(\kappa \right)\rho \right\}\left(w\right)\right]}^{2}\right)+\tau^{2}{∥\beta_{0}-\hat{w}∥}^{2}c_{\rho }^{2}\left(\kappa \right) & =\kappa^{2}r_{\rho }^{2}\left(\kappa \right). \end{array}$$

Corollary 1 shows that, under a homogeneous target design, the general characterization in Theorem 1 reduces to a simpler scalar system. This special case is useful for interpretation and will also serve as a convenient benchmark in the simulation study.

**Remark 2.** 
*The limits on the left-hand side of (10) exist because Assumption 1(f) guarantees convergence of the proportions associated with each pair $\left(L\left(\epsilon_{i}\right),L\left(\lambda_{i}\right)\right)$, where $L(\epsilon_{i})$ and $L(\lambda_{i})$ denote the laws of $\epsilon_{i}$ and $\lambda_{i}$. For the second equation in (10), the ratio can be interpreted through the identity*

$$\frac{{\left[x-\mathrm{prox}\left\{c_{\rho }\left(\kappa \right)\lambda^{2}\rho \right\}\left(x\right)\right]}^{2}}{\lambda^{2}}=c_{\rho }^{2}\left(\kappa \right)\lambda^{2}\psi^{2}\left(\mathrm{prox}\left\{c_{\rho }\left(\kappa \right)\lambda^{2}\rho \right\}\left(x\right)\right)$$

*which is well defined when λ=0. Equivalently, (10) can be written as*

$$\{\begin{array}{cc} \lim_{n\to \infty }\frac{1}{n}\sum_{i=1}^{n}E\left\{{\left[\mathrm{prox}\left\{c_{\rho }\left(\kappa \right)\lambda_{i}^{2}\rho \right\}\right]}^{\prime }\left(W_{i}\right)\right\} & =1-\kappa +\tau c_{\rho }\left(\kappa \right),\\ \kappa \left[\lim_{n\to \infty }\frac{1}{n}\sum_{i=1}^{n}E\left\{c_{\rho }^{2}\left(\kappa \right)\lambda_{i}^{2}\psi^{2}\left(\mathrm{prox}\left\{c_{\rho }\left(\kappa \right)\lambda_{i}^{2}\rho \right\}\left(W_{i}\right)\right)\right\}\right]+\tau^{2}{∥\beta_{0}-\hat{w}∥}^{2}c_{\rho }^{2}\left(\kappa \right) & =\kappa^{2}r_{\rho }^{2}\left(\kappa \right). \end{array}$$

*This representation shows that the expectation in (10) is well defined, and Assumption 1(f) ensures that the relevant limits exist.*

### 3.3. Adaptive Aggregation Against Negative Transfer

We now develop an adaptive aggregation of Trans-RR with the single-task estimator to address negative transfer. Specifically, let ${\hat{\beta }}_{st}$ denote the single-task ridge-regularized robust estimator on the target sample,

(11) $${\hat{\beta }}_{st}=\underset{\beta \in R^{p}}{\mathrm{arg min}}\left[\frac{1}{n}\sum_{i=1}^{n}\rho \left(y_{i}-x_{i}^{⊤}\beta \right)+\frac{\tau_{st}}{2}{∥\beta ∥}^{2}\right]$$

for some constant $\tau_{st}$. Theorem 1 and the discussion above show that negative transfer may happen when the target–source discrepancy $∥\beta_{0}-w_{0}∥$ or the source-stage estimation error is too large. We therefore propose the adaptive Trans-RR estimator to avoid negative transfer, defined for θ∈[0,1] by

(12) $${\hat{\beta }}_{\mathrm{ada}}\left(\theta \right)=\theta \;\hat{\beta }+\left(1-\theta \right)\;{\hat{\beta }}_{st}.$$

Intuitively, ${\hat{\beta }}_{\mathrm{ada}}\left(\theta \right)$ recovers Trans-RR at θ=1 and the single-task estimator at θ=0. Including both endpoints allows the procedure to fall back to either Trans-RR or the single-task estimator, while interior values allow partial transfer. We select the mixing weight θ from data by cross-validation and write ${\hat{\beta }}_{\mathrm{ada}}:={\hat{\beta }}_{\mathrm{ada}}\left(\hat{\theta }\right)$ for the resulting estimator.

We tune the ridge penalties before selecting θ. The source penalty $\tau_{1}$ is tuned by cross-validation on the source sample, yielding $\hat{w}$ via (3). With $\hat{w}$ fixed, the target penalty τ is tuned by cross-validation on the target sample through (4). The single-task penalty $\tau_{st}$ is tuned by cross-validation on the target sample. To select θ, we then use a *K*-fold partition ${\left\{V_{k}\right\}}_{k=1}^{K}$ of the target sample, drawn independently of the partitions used to tune τ and $\tau_{st}$. Here, $V_{k}$ denotes the validation index set of fold *k*.

For each k=1,…,K, let ${\hat{\beta }}^{\left(-k\right)}$ be the output of Algorithm 1 applied to the full source data and $\left\{\left(x_{i},y_{i}\right):i\notin V_{k}\right\}$ at the tuned $\left(\tau_{1},\tau \right)$. Let ${\hat{\beta }}_{st}^{\left(-k\right)}$ be the solution of (11) on $\left\{\left(x_{i},y_{i}\right):i\notin V_{k}\right\}$ at the tuned $\tau_{st}$. We reuse $\tau_{1}$, τ, and $\tau_{st}$ across all *K* folds rather than re-tuning per fold, which would multiply the penalty-tuning cost by *K*. We then choose

(13) $$\hat{\theta }=\underset{\theta \in \Theta }{\mathrm{arg min}}\frac{1}{n}\sum_{k=1}^{K}\sum_{i\in V_{k}}L\left(y_{i}-x_{i}^{⊤}\left[\theta \;{\hat{\beta }}^{\left(-k\right)}+\left(1-\theta \right)\;{\hat{\beta }}_{st}^{\left(-k\right)}\right]\right),$$

where $L:R\to R_{\geq 0}$ is a validation loss and Θ⊂[0,1] is a finite candidate set. A natural default is the absolute-error loss L(t)=|t|, which matches the criterion used to tune the ridge penalties. We refer to ${\hat{\beta }}_{\mathrm{ada}}$ as the adaptive Trans-RR estimator, denoted Trans-RR-Ada in the following numerical experiments. Algorithm 2 summarizes the procedure.    
**Algorithm 2:** Adaptive Trans-RR algorithm
 **Input:** target data ${\left\{\left(x_{i},y_{i}\right)\right\}}_{i=1}^{n}$, source data ${\left\{\left(x_{i}^{\left(1\right)},y_{i}^{\left(1\right)}\right)\right\}}_{i=1}^{n_{1}}$, fold count *K*, 
    candidate set Θ⊂[0,1], validation loss *L*
 **Output:** the adaptive estimator ${\hat{\beta }}_{\mathrm{ada}}$ and the selected weight $\hat{\theta }$
 Step 1. Tune $\tau_{1}$, τ, and $\tau_{st}$ by cross-validation on the corresponding samples. 
 Compute $\hat{\beta }$ from Algorithm 1 and ${\hat{\beta }}_{st}$ from (11).
 Step 2. Draw a *K*-fold partition ${\left\{V_{k}\right\}}_{k=1}^{K}$ of the target indices, independent of the
 partitions used in Step 1.
 Step 3. For each k=1,…,K, let ${\hat{\beta }}^{\left(-k\right)}$ be the output of Algorithm 1 applied to the 
 full source data and $\left\{\left(x_{i},y_{i}\right):i\notin V_{k}\right\}$ at the tuned $\left(\tau_{1},\tau \right)$. Let ${\hat{\beta }}_{st}^{\left(-k\right)}$ be the 
 solution of (11) on $\left\{\left(x_{i},y_{i}\right):i\notin V_{k}\right\}$ at the tuned $\tau_{st}$.
 Step 4. Compute
$$\hat{\theta }=\underset{\theta \in \Theta }{\mathrm{arg min}}\frac{1}{n}\sum_{k=1}^{K}\sum_{i\in V_{k}}L\left(y_{i}-x_{i}^{⊤}\left[\theta {\hat{\beta }}^{\left(-k\right)}+\left(1-\theta \right){\hat{\beta }}_{st}^{\left(-k\right)}\right]\right).$$
 Step 5. Output ${\hat{\beta }}_{\mathrm{ada}}=\hat{\theta }\hat{\beta }+\left(1-\hat{\theta }\right){\hat{\beta }}_{st}$ and $\hat{\theta }$.

### 3.4. Applicability and Limitations

Theorem 1 and Corollary 1 are derived under Assumptions 1 and 2, which include three structural conditions: identity covariance $\mathrm{cov}\left(x_{i}\right)=I_{p}$, the moderate–dimensional regime p/n→κ∈(0,∞), and a twice-differentiable robust loss. Under these assumptions, the asymptotic $l_{2}$ estimation error of Trans-RR equals the deterministic limit $r_{\rho }\left(\kappa \right)$, in agreement with the numerical results of Section 4. When any of these assumptions fails, Theorem 1 no longer applies.

The adaptive aggregation of Section 3.3, by contrast, is constructed without invoking these structural assumptions. Its mixing weight $\hat{\theta }$ is selected by cross-validation on the target sample and serves as a data-driven safeguard against negative transfer. Section 4.4 provides numerical support under AR(1)-correlated predictors across all three noise cases: a transition between positive and negative transfer is observed near h=1, and Trans-RR-Ada continues to track the better of the two base estimators.

## 4. Simulation

In this section, we conduct numerical studies to support the theoretical results. We set the dimension of both target and source data to be p∈{200,400,800}. We set n=p,p/4 and $n_{1}=2p,p/2$, corresponding to moderate–dimensional settings with κ=1,4 and $\kappa_{1}=1/2,2$. To generate data, we set $x_{i}=\lambda_{i}X_{i}$ and $x_{i}^{\left(1\right)}=\lambda_{i}^{\left(1\right)}X_{i}^{\left(1\right)}$, where $X_{i}$ and $X_{i}^{\left(1\right)}$ have i.i.d. N(0,1) entries. We consider three different cases for the choices of $\lambda_{i}$’s, $\epsilon_{i}$’s, $\lambda_{i}^{\left(1\right)}$’s and $\epsilon_{i}^{\left(1\right)}$’s:

- **Case I:** $\lambda_{i}=1$ for i=1,…,n and $\lambda_{j}^{\left(1\right)}=1$ for $j=1,\ldots ,n_{1}$. The target errors $\epsilon_{i}$ are i.i.d. N(0,1), and the source errors $\epsilon_{j}^{\left(1\right)}$ are i.i.d. $N(0,2^{2})$.
- **Case II:** The variables $\lambda_{i}$ and $\lambda_{j}^{\left(1\right)}$ are i.i.d. Unif$\left(0,\sqrt{3}\right)$, while $\epsilon_{i}$ and $\epsilon_{j}^{\left(1\right)}$ are i.i.d. Cauchy(0,1) and Cauchy(0,2), respectively.
- **Case III:** In both the target and source studies, half of the observations are generated as in Case I and the other half are generated as in Case II.

Case I is a standard Gaussian setup for linear regression. Case II features a non-Gaussian design and heavy-tailed errors. Case III is a mixture of the two cases and is designed to test the effectiveness of our theoretical results under non-identical $x_{i}$’s and $\epsilon_{i}$’s.

### 4.1. Validity of Theoretical Results

We first evaluate the validity of the proposed scalar $r_{\rho }$ in Theorem 1. For each setting, we generate $\beta^{*}$ and $w^{*}$ once with i.i.d. Unif(0,1) entries and set $\beta_{0}=\beta^{*}/\sqrt{n}$ and $w_{0}=w^{*}/\sqrt{n}$. This construction yields diffuse coefficients whose Euclidean norms remain bounded as *n* grows. These coefficient vectors are fixed across the 1000 replications, while the target and source samples are regenerated in each replicate. In each replicate, we first compute $\hat{w}$ from the source sample and then obtain $\hat{\beta }$ by applying Algorithm 1, using the smoothed Huber loss (6) for both $\tilde{\rho }$ and ρ with parameters δ=1.35 and η=0.1. We fix $\tau =\tau_{1}=1$ and repeat each setup 1000 times.

**Figure 1:**
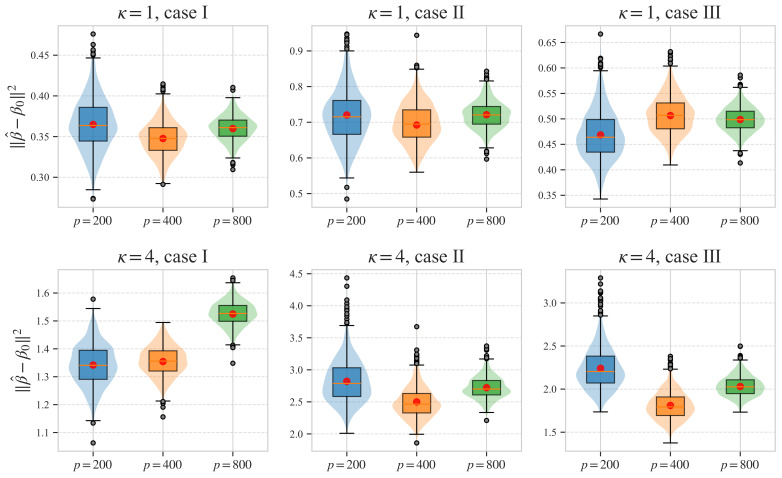
Boxplot of $∥\hat{\beta }-\beta_{0}{∥}^{2}$ over 1000 simulations. The red point in each boxplot represents the theoretical value $r_{\rho }^{2}$ from Theorem 1. Panels from top to bottom are for κ=1,4, respectively, while panels from left to right are for cases I,II,III, respectively.

Figure 1 presents boxplots of the estimation error $∥\hat{\beta }-\beta_{0}{∥}^{2}$ for cases I–III and κ=1,4. The red point in each boxplot marks the theoretical value $r_{\rho }^{2}$, obtained by numerically solving the system in Theorem 1 under the corresponding simulation specification. We observe that the empirical distribution of $∥\hat{\beta }-\beta_{0}{∥}^{2}$ is centered close to this value, and its dispersion decreases as *n* and *p* become larger. Table 1 shows the mean and standard deviation (SD) of $∥\hat{\beta }-\beta_{0}{∥}^{2}$ (denoted as ${\hat{r}}^{2}$) and the corresponding $r_{\rho }^{2}$ for each setup. As dimensionality increases, that is, as κ increases from 1 to 4, both the mean error and its variability increase, indicating that estimation becomes more difficult in more challenging moderate–dimensional regimes. The average estimation error also grows with heavier-tailed errors, highlighting the difficulty of estimation under such conditions. Results under case III demonstrate that Theorem 1 is effective in handling non-identical $x_{i}$’s and $\epsilon_{i}$’s. Overall, the findings in Figure 1 and Table 1 align well with the theoretical predictions of Theorem 1.

### 4.2. Theoretical Estimation Error Curves

**Figure 2:**
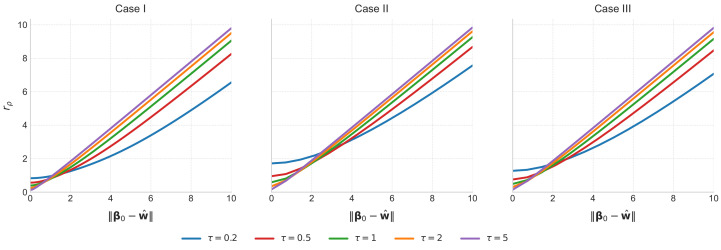
Theoretical curves of $r_{\rho }$ as a function of $∥\beta_{0}-\hat{w}∥$ for five values of τ under cases I–III, obtained by numerically solving Corollary 1. The three panels correspond to cases I, II, and III, respectively.

We take ρ to be our recommended smoothed Huber loss (6) with (δ,η)=(1.35,0.1) and fix κ=1. We numerically solve the associated scalar system while varying the discrepancy term $∥\beta_{0}-\hat{w}∥$ that enters the second equation of Theorem 1. Figure 2 plots the resulting curves of $r_{\rho }$ for five values of τ under cases I–III. In all three cases, the curves are monotonically increasing over the displayed range, so a larger $∥\beta_{0}-\hat{w}∥$ corresponds to a larger asymptotic estimation error in this setting.

### 4.3. Comparison with Existing Methods

To evaluate when transfer is beneficial, we compare our method with several competing procedures across the three scenarios described above. We set p=400, n=p and $n_{1}=2p$. We generate $\beta_{0}=\beta^{*}/∥\beta^{*}∥$, where $\beta^{*}={\left(\beta_{1}^{*},\ldots ,\beta_{p}^{*}\right)}^{⊤}$ has i.i.d. Unif(0,1) entries. To control the transfer strength $h=∥\delta_{0}∥$, we set

$$\delta_{0}=\exp\left(c_{d}\right)\cdot 1_{p}/\sqrt{p},\;c_{d}\in \left\{-2.0,-1.5,-1.0,-0.5,0,0.5,1.0\right\},$$

and define the source coefficient by $w_{0}=\beta_{0}-\delta_{0}$. By varying $c_{d}$ from −2.0 to 1.0, we obtain *h* ranging from approximately 0.135 to 2.718, providing a comprehensive evaluation across different levels of source-target similarity. For each value of $c_{d}$, the pair $\left(\beta_{0},w_{0}\right)$ is fixed across the 500 replications, and only the data are regenerated.

We compare four ridge-type methods (Single-RR, Trans-RR, Trans-RR-Ada, and Pooled-RR) and two lasso baselines (Single-Lasso and Trans-Lasso):

1. **Single-RR**: The single-task estimator ${\hat{\beta }}_{st}$ in (11), fit to the target sample alone.
2. **Trans-RR**: The two-stage estimator $\hat{\beta }=\hat{w}+\hat{\delta }$ in (5), computed by Algorithm 1.
3. **Trans-RR-Ada**: The adaptive aggregate ${\hat{\beta }}_{\mathrm{ada}}$ in (12), computed by Algorithm 2 with K=5, absolute-error loss L(t)=|t|, and weight grid Θ={0,0.1,…,0.9,1}.
4. **Pooled-RR**: The same robust ridge fit applied to the concatenation of the source and target samples.
5. **Single-Lasso**: The lasso on the target sample, with its regularization parameter chosen by five-fold cross-validation.
6. **Trans-Lasso**: The two-stage transfer-lasso of [8], in which a cross-validated source-stage lasso estimates $w_{0}$ and a cross-validated target-stage lasso fits the residual $y_{i}-x_{i}^{⊤}\hat{w}$ on the target sample.

The four ridge-type methods all use the smoothed Huber loss (6) with (δ,η)=(1.35,0.1). Each ridge penalty is tuned by five-fold cross-validation under the mean absolute error criterion, over the grid $\left\{3^{a}:a=-2,-3/2,\ldots ,2\right\}$. For Trans-RR, the source penalty $\tau_{1}$ in (3) is tuned on the source sample, yielding $\hat{w}$ as the minimizer of (3) at the tuned $\tau_{1}$. With $\hat{w}$ held fixed, the target penalty τ in (4) is then tuned on the target sample.

**Figure 3:**
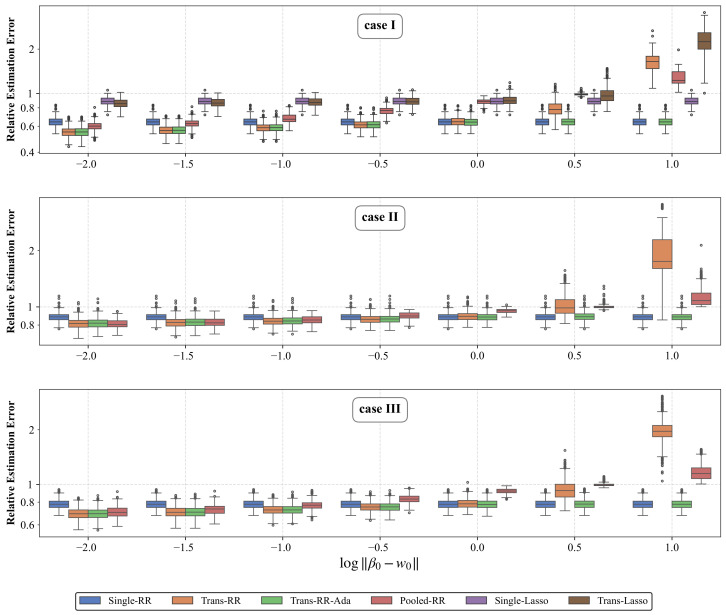
Boxplots of relative estimation errors (log scale) across 500 replications for varying $∥\beta_{0}-w_{0}∥$ under cases I–III, with p=400 and n=400. Case I includes all six methods, while cases II and III include only the four ridge-type procedures. Panels from top to bottom are for cases I,II,III, respectively.

Performance is summarized by the relative estimation error $∥\hat{\beta }-\beta_{0}{∥}^{2}/{∥\beta_{0}∥}^{2}$. Figure 3 presents boxplots of these errors on a logarithmic scale, for varying values of *h* across cases I–III. We report all six estimators under Case I. In Cases II and III, the noise is heavy-tailed, and the lasso methods are not designed for robustness. Their fits failed to converge to stable estimates, so we restrict these two cases to the ridge-type methods.

Under Case I, Trans-RR compares favorably with both lasso baselines, consistent with the non-sparse structure assumed throughout the paper. Among the ridge-type methods, Trans-RR outperforms Pooled-RR for small and moderate *h* across all three cases. Pooled-RR fits the source and target observations together as if they shared one coefficient, so its error reflects two mismatches: the gap between $\beta_{0}$ and $w_{0}$, and the difference between source and target noise levels. As *h* decreases, the gap between Pooled-RR and Trans-RR narrows, in line with the theoretical expectation that smaller *h* indicates greater similarity between the two domains.

More importantly, the comparison with Single-RR reveals the transition between positive and negative transfer. When *h* is small, Trans-RR achieves the lowest relative error among the ridge-type methods. As *h* grows, its advantage shrinks and eventually reverses. In our experiments, this turnover occurs near h=1. This is consistent with the numerical evidence in Section 3, since transfer is more favorable when the source-stage estimator is closer to the target coefficient. The same qualitative pattern appears in Cases II and III, where the transition near h=1 persists under heavy-tailed errors and heterogeneous designs.

Trans-RR-Ada provides a data-driven safeguard against this negative transfer. Figure 3 shows that it tracks the better of Single-RR and Trans-RR for every value of *h*. For small *h*, Trans-RR-Ada essentially coincides with Trans-RR, while for large *h* it coincides with Single-RR. At the transition h=1, where Single-RR and Trans-RR are comparable, Trans-RR-Ada stays close to the better one.

### 4.4. Robustness to Non-Identity Covariance

The asymptotic theory in Section 3 assumes $\mathrm{cov}\left(x_{i}\right)=I_{p}$. To verify that our methods remain effective under non-identity covariance, we re-run the comparison of Section 4.3 under AR(1) covariance $\sum_{jk}=\rho^{\left|j-k\right|}$ with ρ=0.6, across all three noise cases I, II, and III. All other settings, including the four ridge-type methods Single-RR, Trans-RR, Trans-RR-Ada, and Pooled-RR and the tuning protocol, match Section 4.3.

**Figure 4:**
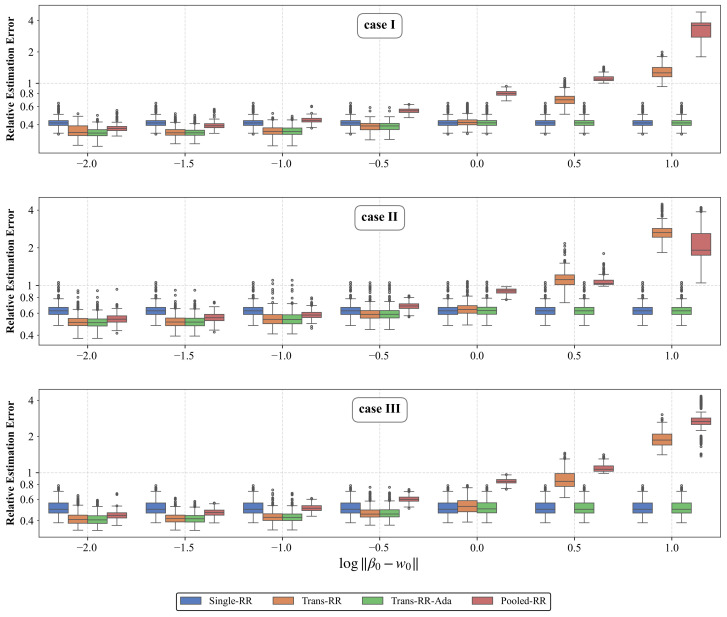
Robustness check under AR(1) correlated predictors. Boxplots of relative estimation errors (log scale) across 500 replications for varying $∥\beta_{0}-w_{0}∥$ under cases I–III with $x_{i}∼N\left(0,\sum_{\rho }\right)$, where $\sum_{\rho ,jk}=\rho^{\left|j-k\right|}$ and ρ=0.6. Panels from top to bottom are for cases I, II, and III, respectively. The four ridge-type procedures, Single-RR, Trans-RR, Trans-RR-Ada, and Pooled-RR are shown.

Figure 4 reports the resulting boxplots. The qualitative pattern of the i.i.d. comparison persists in all three cases. For small *h*, Trans-RR achieves the lowest error among the ridge-type methods. As *h* grows, its advantage shrinks and eventually reverses, with the transition again occurring near h=1. Trans-RR-Ada tracks the better of Single-RR and Trans-RR across all values of *h*. The negative-transfer transition and the safeguard role of Trans-RR-Ada are therefore not specific to the identity–covariance assumption underlying Theorem 1. This suggests that Trans-RR may remain effective under non-identity covariance.

### 4.5. Sensitivity to Tuning Choices

The comparison in Figure 3 fixes the smoothed Huber loss with (δ,η)=(1.35,0.1) and selects each ridge penalty by five-fold cross-validation under the mean absolute error criterion, on a common geometric grid of nine values from 1/9 to 9. This subsection examines whether the qualitative findings of that comparison are stable under perturbations to four tuning choices: the smoothed Huber parameters (δ,η), the cross-validation criterion, the robust loss family, and the ridge penalty grid. Each perturbation re-runs the full simulation with M=500 replications per setting.

#### 4.5.1. Choice of (δ,η)

The default δ=1.35 is the standard Huber tuning. The smoothing parameter η=0.1 closely approximates the unsmoothed Huber loss and keeps $\rho_{\eta }$ twice continuously differentiable, as required by Assumption 1(b). In the sensitivity experiment, we vary δ over {1.0,1.35,2.0} and η over {0.05,0.10,0.20}, giving nine (δ,η) pairs, and re-run the full simulation across the three cases and seven discrepancy values *h* for every pair. Within each (case,h) combination, varying the (δ,η) pair over the nine settings changes each method’s mean error by less than 7% (median 1.5%) of its mean. The four-method ranking matches the default (1.35,0.10) ranking in 171 of the 189 (case,h,(δ,η)) cells. The qualitative findings are therefore stable under this perturbation. Table 2 displays the four method means as a (δ,η) heatmap at h=1 in each of the three cases. Within each 3×3 grid, the entries vary only slightly and the four-method ordering is the same in every cell, illustrating the two findings stated above. Appendix B reports the heatmaps at the other six values of *h*, where the same pattern holds.

#### 4.5.2. Choice of Cross-Validation Criterion

The default selects each ridge penalty by five-fold cross-validation under the mean absolute error criterion. We re-run the simulation with every cross-validation loss changed to mean squared error, keeping the smoothed Huber loss for estimation. Table 3 compares the mean estimation errors under the two criteria across all cases and values of *h*.

Under Case I, the two criteria yield nearly identical mean errors and the same four-method ranking at every *h*. Under Cases II and III, the mean errors of all four methods become substantially larger, and two qualitative findings of Section 4.3 no longer hold. First, Trans-RR has a larger mean error than Single-RR at every *h* in both heavy-tailed cases, so the range on which transfer helps disappears entirely. Second, Trans-RR-Ada no longer approximates min(Single-RR, Trans-RR) in most settings of the heavy-tailed cases. This may result from the heavy tails of Cauchy errors, under which MSE-based cross-validation is more sensitive to extreme residuals than MAE-based cross-validation. MAE-CV is therefore the appropriate default for heavy-tailed errors, and the conclusions of Section 4.3 are specific to this choice.

#### 4.5.3. Choice of Robust Loss

In order to assess the sensitivity to the specific form of the robust loss, we replace the smoothed Huber loss in (6) with the pseudo-Huber loss $\rho_{P}\left(t;\delta \right)=\delta^{2}\left(\sqrt{1+{\left(t/\delta \right)}^{2}}-1\right)$ at the same δ=1.35. Table 4 compares the mean estimation errors under the two losses across all cases and values of *h*.

The mean errors are nearly identical to those under the smoothed Huber loss across all 21 settings. The four-method ranking matches the default in nearly every setting, and the few mismatches involve methods whose mean errors are essentially tied. The negative-transfer transition stays at the same value of *h* in every case, and Trans-RR-Ada continues to track min(Single-RR, Trans-RR) as closely as under the default. The qualitative findings of Section 4.3 are insensitive to this choice, consistent with Theorem 1.

#### 4.5.4. Choice of Ridge Penalty Grid

The four ridge penalties $\tau_{1}$, τ, $\tau_{\mathrm{st}}$, and $\tau_{p}$ are tuned by five-fold cross-validation over a common geometric grid of nine values from 1/9 to 9. We probe the sensitivity to this grid in two complementary ways: extending the grid to wider values, and forcing all four penalties to a common fixed value with no cross-validation.

We first extend the grid to thirteen values from 1/27 to 27 on the same geometric scale, which contains the default grid as a strict subset. Table 5 compares the mean estimation errors under the default and the wide grid across all cases and values of *h*. The impact on estimation is negligible: each method’s mean error barely changes across settings, the four-method ranking is unchanged except at settings with near-tied means, the negative-transfer transition does not move, and Trans-RR-Ada continues to track min(Single-RR, Trans-RR) as closely as under the default. The default grid is therefore wide enough on both sides, and the qualitative findings of Section 4.3 do not depend on the choice of upper or lower endpoint.

We next force all four ridge penalties to a common fixed value τ∈{1/3,1,3,9}, removing the ridge cross-validation entirely. Trans-RR-Ada’s mixing weight θ is still selected by five-fold cross-validation on the target sample (Algorithm 2). Table 6 reports the mean estimation errors at the four fixed τ values across all cases and values of *h*. At every fixed τ, the mean error of Trans-RR grows monotonically with *h* in all three cases, consistent with the theoretical risk curves of Figure 2. The absolute levels and the four-method ranking do vary substantially across the four τ values, but the qualitative dependence on *h* predicted by the theory is preserved at every τ.

The Trans-RR-Ada safeguard nevertheless remains effective: its mean error stays close to min(Single-RR, Trans-RR) in every one of the 84 settings, just as under cross-validated penalties. The agreement between Trans-RR-Ada and min(Single-RR, Trans-RR) is therefore robust to a misspecified ridge penalty.

## 5. Real Data Analysis

We evaluate the proposed transfer procedure on the near-infrared (NIR) spectral dataset from the 2002 International Diffuse Reflectance Conference (IDRC) “Shootout” competition. The data consist of pharmaceutical tablet measurements collected by two spectrometers, with ASSAY as the response. For each instrument, the dataset contains a training sample of size 460 and an external test sample of size 155. Each spectrum is recorded at 650 wavelengths, yielding a moderate–dimensional regression problem.

Let *X* and $X_{1}$ denote the spectra from the two instruments. We consider two transfer directions. In Direction A, *X* is the target domain and $X_{1}$ is the source domain. In Direction B, the roles are reversed. In each repetition, we randomly split the training sample of the target instrument into two parts of sizes 160 and 300. The subset of size 160 is used as the target training sample. The remaining 300 tablets are matched with their measurements from the other instrument to form the source training sample. When *X* is the target domain, the target fit is constructed from 160 observations in *X*, and the source fit is constructed from the corresponding 300 observations in $X_{1}$. The same scheme is applied symmetrically in the reverse direction. The external test sample of the target instrument is used for evaluation. We repeat this procedure 20 times to reduce Monte Carlo variability.

The preprocessing used in both transfer directions has two stages. First, we thin the spectrum by retaining every fourth wavelength of the original 650, yielding p=163 predictors. Two considerations motivate this thinning. Adjacent NIR wavelengths record highly overlapping absorption signals, so a four-fold thinning preserves nearly all the spectral information. This is standard practice in NIR chemometrics. A step of four also gives p=163≈n=160, the same p/n≈1 setting as in our simulations.

Second, for each domain, predictors are whitened using its own training sample,

$$\tilde{X}=\left(X-\hat{\mu }\right){\hat{\sum }}^{-1/2},$$

where $\hat{\mu }$ and $\hat{\sum }$ are estimated from that training sample, and the same transformation is applied to the associated test sample. Whitening decorrelates the highly collinear NIR predictors so that the sample second-moment matrix of $\tilde{X}$ approximates $I_{p}$, which is the design assumption underlying Theorem 1. The response is centered using the mean of the corresponding training response, and the same centering constant is used for the associated test response. All preprocessing parameters are thus estimated only on training data and carried over to the test set, avoiding information leakage.

We compare six methods: Single-RR, Trans-RR, the adaptive Trans-RR estimator ${\hat{\beta }}_{\mathrm{ada}}$ from Section 3.3 (denoted Trans-RR-Ada in the table), Pooled-RR, Single-Lasso, and Trans-Lasso. For the ridge-type methods, we use the smoothed Huber loss in (6) with (δ,η)=(1.35,0.1). All tuning parameters are selected by 5-fold cross-validation with mean absolute error as the validation criterion, from the common grid

$$G=\{10^{-5},10^{-4.5},10^{-4},\ldots ,10^{1}\}.$$

Performance is measured by the test-set RMSE,

$$\mathrm{RMSE}=\sqrt{\frac{1}{n_{\mathrm{test}}}\sum_{i=1}^{n_{\mathrm{test}}}{\left(y_{i}-{\hat{y}}_{i}\right)}^{2}},$$

where $y_{i}$ and ${\hat{y}}_{i}$ are on the original ASSAY scale (predictions are uncentered before evaluation) and $n_{\mathrm{test}}=155$ in both transfer directions.

**Figure 5:**
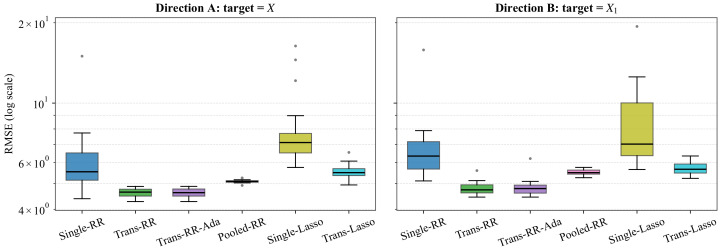
Distribution of RMSE across the 20 repeated random splits for each of the six methods, in the two transfer directions. Boxes show the inter-quartile range, whiskers extend to 1.5 × IQR, and circles mark splits beyond that.

Table 7 reports the average RMSE and its standard deviation over the 20 repetitions, and Figure 5 displays the corresponding distributional information. Trans-RR achieves the smallest mean RMSE in both transfer directions, with relatively small variability across the 20 splits. The adaptive variant Trans-RR-Ada matches Trans-RR within 0.03 in mean RMSE in both directions, indicating that the source and target are close enough for transfer to be uniformly beneficial on this dataset. Pooled-RR ranks third behind the two transfer-ridge methods and is uniformly dominated by Trans-RR, suggesting that naive pooling fails to account for cross-instrument differences. The lasso methods are less competitive overall, especially Single-Lasso. This is consistent with the non-sparse structure of NIR spectra, where relevant information is distributed over broad wavelength regions rather than concentrated on a small subset of predictors.

We further checked the procedure for four starting offsets of the every-fourth-wavelength thinning and for the same procedure without whitening. Across all five resulting preprocessing variants and both transfer directions, Trans-RR and Trans-RR-Ada are the top two methods by mean RMSE, and their mean RMSE values differ by at most 0.05. Mean RMSE for all six methods in each variant and direction is reported in Appendix C. The results, together with these robustness checks, therefore support the effectiveness of the proposed Trans-RR method.

## 6. Discussion

This paper introduces Trans-RR, a robust transfer-learning approach for moderate–dimensional linear regression. It extends transfer-learning ideas of [7,8] to a setting with non-sparse coefficients and heavy-tailed errors, without relying on sparsity assumptions or moment restrictions on the errors. The theory and the numerical results show that negative transfer can occur when the source study is not sufficiently informative for the target study. To guard against this, we also propose an adaptive aggregation of Trans-RR with the single-task estimator that selects the mixing weight by cross-validation. The theoretical results, simulation studies, and real-data analysis support the effectiveness of both procedures. A natural direction for further study is to identify the choice of loss function that minimizes the asymptotic estimation error in our framework. 

## Figures and Tables

**Table 1:** Mean and SD (in parentheses) of $∥\hat{\beta }-\beta_{0}{∥}^{2}$ (denoted as ${\hat{r}}^{2}$) and the corresponding $r_{\rho }^{2}$ over 1000 simulations. Rows are indexed by (p,n), with $n_{1}=2p$ when κ=1 and $n_{1}=p/2$ when κ=4.

(p,n)	Case I		Case II		Case III	
	${\hat{r}}^{2}$	$r_{\rho }^{2}$	${\hat{r}}^{2}$	$r_{\rho }^{2}$	${\hat{r}}^{2}$	$r_{\rho }^{2}$
κ=1						
(200, 200)	0.3653 (0.0318)	0.3649	0.7163 (0.0738)	0.7204	0.4683 (0.0478)	0.4685
(400, 400)	0.3472 (0.0208)	0.3477	0.6970 (0.0549)	0.6923	0.5076 (0.0368)	0.5065
(800, 800)	0.3603 (0.0151)	0.3598	0.7206 (0.0374)	0.7212	0.4989 (0.0238)	0.4986
κ=4						
(200, 50)	1.3415 (0.0734)	1.3419	2.8222 (0.3311)	2.8216	2.2410 (0.2407)	2.2415
(400, 100)	1.3565 (0.0531)	1.3544	2.4896 (0.2363)	2.4996	1.8087 (0.1590)	1.8102
(800, 200)	1.5261 (0.0427)	1.5247	2.7226 (0.1693)	2.7219	2.0342 (0.1153)	2.0301

**Table 2:** Sensitivity of relative estimation error to the smoothed Huber parameters (δ,η) at the transition discrepancy h=1. Each entry reports Single-RR/Trans-RR/Trans-RR-Ada/Pooled-RR mean estimation error over M=500 replications. The three blocks correspond to the three error distributions of Section 4.3.

Case I (Gaussian Errors)			
δ∖η	0.05	0.10	0.20
1.00	0.653/0.655/0.651/0.883	0.654/0.656/0.652/0.883	0.656/0.658/0.654/0.883
1.35	0.644/0.647/0.642/0.882	0.645/0.647/0.642/0.882	0.646/0.648/0.643/0.882
2.00	0.641/0.642/0.637/0.885	0.640/0.642/0.637/0.885	0.641/0.643/0.637/0.885
Case II (Cauchy Errors)			
δ∖η	0.05	0.10	0.20
1.00	0.884/0.893/0.884/0.952	0.884/0.892/0.884/0.952	0.884/0.893/0.884/0.952
1.35	0.883/0.893/0.884/0.951	0.883/0.892/0.883/0.951	0.883/0.892/0.884/0.951
2.00	0.890/0.899/0.891/0.951	0.889/0.899/0.890/0.951	0.889/0.899/0.890/0.951
Case III (Mixture Errors)			
δ∖η	0.05	0.10	0.20
1.00	0.779/0.786/0.779/0.921	0.780/0.787/0.779/0.921	0.780/0.787/0.779/0.921
1.35	0.781/0.788/0.781/0.920	0.781/0.787/0.780/0.920	0.781/0.787/0.780/0.921
2.00	0.794/0.801/0.793/0.924	0.793/0.800/0.792/0.924	0.792/0.800/0.791/0.923

**Table 3:** Sensitivity of relative estimation error to the cross-validation criterion. All settings are as in Figure 3, except that every cross-validation loss (used to select $\tau_{1}$, τ, $\tau_{\mathrm{st}}$, $\tau_{p}$, and θ) is changed from MAE to MSE. Each entry reports the default (MAE-CV) and the MSE-CV mean estimation error, in the format “default/MSE-CV”, over M=500 replications.

Case	*h*	Single-RR	Trans-RR	Trans-RR-Ada	Pooled-RR
I	0.135	0.645/0.644	0.550/0.546	0.550/0.546	0.600/0.601
	0.223	0.645/0.644	0.565/0.561	0.566/0.561	0.628/0.629
	0.368	0.645/0.644	0.588/0.585	0.589/0.585	0.678/0.681
	0.607	0.645/0.644	0.616/0.614	0.617/0.615	0.764/0.770
	1.000	0.645/0.644	0.647/0.645	0.642/0.641	0.882/0.888
	1.649	0.645/0.644	0.791/0.793	0.645/0.644	0.990/0.991
	2.718	0.645/0.644	1.639/1.646	0.645/0.644	1.261/1.479
II	0.135	0.883/1.772	0.816/2.582	0.819/1.895	0.811/1.324
	0.223	0.883/1.772	0.826/2.597	0.829/1.904	0.829/1.326
	0.368	0.883/1.772	0.840/2.618	0.843/1.915	0.855/1.353
	0.607	0.883/1.772	0.860/2.640	0.862/1.920	0.898/1.401
	1.000	0.883/1.772	0.892/2.745	0.883/1.963	0.951/1.497
	1.649	0.883/1.772	1.021/2.994	0.887/2.020	1.005/1.679
	2.718	0.883/1.772	1.898/3.827	0.883/2.108	1.123/2.223
III	0.135	0.781/1.108	0.693/1.319	0.692/1.094	0.706/0.944
	0.223	0.781/1.108	0.707/1.334	0.707/1.102	0.729/0.966
	0.368	0.781/1.108	0.727/1.358	0.727/1.114	0.770/0.993
	0.607	0.781/1.108	0.754/1.395	0.754/1.138	0.834/1.054
	1.000	0.781/1.108	0.787/1.473	0.780/1.161	0.920/1.155
	1.649	0.781/1.108	0.941/1.749	0.783/1.194	0.998/1.368
	2.718	0.781/1.108	1.985/2.646	0.781/1.274	1.181/1.961

**Table 4:** Sensitivity of relative estimation error to the choice of robust loss. All settings are as in Figure 3, except that the smoothed Huber loss is replaced by the pseudo-Huber loss $\rho_{P}\left(t;\delta \right)=\delta^{2}\left(\sqrt{1+{\left(t/\delta \right)}^{2}}-1\right)$ with δ=1.35 (the smoothing parameter η is no longer needed). Each entry reports the default (smoothed Huber) and the pseudo-Huber mean estimation error, in the format “default/pseudo-Huber”, over M=500 replications.

Case	*h*	Single-RR	Trans-RR	Trans-RR-Ada	Pooled-RR
I	0.135	0.645/0.645	0.550/0.545	0.550/0.546	0.600/0.595
	0.223	0.645/0.645	0.565/0.561	0.566/0.562	0.628/0.625
	0.368	0.645/0.645	0.588/0.585	0.589/0.586	0.678/0.675
	0.607	0.645/0.645	0.616/0.615	0.617/0.616	0.764/0.763
	1.000	0.645/0.645	0.647/0.647	0.642/0.643	0.882/0.883
	1.649	0.645/0.645	0.791/0.797	0.645/0.645	0.990/0.995
	2.718	0.645/0.645	1.639/1.642	0.645/0.645	1.261/1.299
II	0.135	0.883/0.891	0.816/0.828	0.819/0.830	0.811/0.823
	0.223	0.883/0.891	0.826/0.836	0.829/0.839	0.829/0.839
	0.368	0.883/0.891	0.840/0.850	0.843/0.852	0.855/0.865
	0.607	0.883/0.891	0.860/0.869	0.862/0.872	0.898/0.905
	1.000	0.883/0.891	0.892/0.900	0.883/0.891	0.951/0.954
	1.649	0.883/0.891	1.021/1.023	0.887/0.895	1.005/1.007
	2.718	0.883/0.891	1.898/1.871	0.883/0.891	1.123/1.129
III	0.135	0.781/0.793	0.693/0.703	0.692/0.703	0.706/0.713
	0.223	0.781/0.793	0.707/0.717	0.707/0.718	0.729/0.738
	0.368	0.781/0.793	0.727/0.740	0.727/0.740	0.770/0.777
	0.607	0.781/0.793	0.754/0.765	0.754/0.765	0.834/0.842
	1.000	0.781/0.793	0.787/0.798	0.780/0.791	0.920/0.925
	1.649	0.781/0.793	0.941/0.950	0.783/0.794	0.998/1.002
	2.718	0.781/0.793	1.985/1.972	0.781/0.793	1.181/1.199

**Table 5:** Sensitivity of relative estimation error to the ridge-penalty cross-validation grid. The default grid contains 9 values from 1/9 to 9 on a geometric scale. The wide grid extends this to 13 values from 1/27 to 27 on the same geometric scale, and contains the default grid as a strict subset. All other settings are as in Figure 3, with M=500 replications per cell. Each entry reports Single-RR/Trans-RR/Trans-RR-Ada/Pooled-RR mean estimation error.

Case	*h*	Default Grid (9 pts, [1/9, 9])	Wide Grid (13 pts, [1/27, 27])
I	0.135	0.645/0.550/0.550/0.600	0.645/0.550/0.550/0.600
I	0.223	0.645/0.565/0.566/0.628	0.645/0.565/0.566/0.628
I	0.368	0.645/0.588/0.589/0.678	0.645/0.589/0.590/0.678
I	0.607	0.645/0.616/0.617/0.764	0.645/0.618/0.619/0.764
I	1.000	0.645/0.647/0.642/0.882	0.645/0.648/0.642/0.883
I	1.649	0.645/0.791/0.645/0.990	0.645/0.791/0.645/0.990
I	2.718	0.645/1.639/0.645/1.261	0.645/1.639/0.645/1.261
II	0.135	0.883/0.816/0.819/0.811	0.887/0.824/0.826/0.811
II	0.223	0.883/0.826/0.829/0.829	0.887/0.835/0.838/0.829
II	0.368	0.883/0.840/0.843/0.855	0.887/0.850/0.852/0.856
II	0.607	0.883/0.860/0.862/0.898	0.887/0.869/0.871/0.900
II	1.000	0.883/0.892/0.883/0.951	0.887/0.896/0.888/0.955
II	1.649	0.883/1.021/0.887/1.005	0.887/1.021/0.891/1.006
II	2.718	0.883/1.898/0.883/1.123	0.887/1.899/0.887/1.120
III	0.135	0.781/0.693/0.692/0.706	0.782/0.694/0.694/0.706
III	0.223	0.781/0.707/0.707/0.729	0.782/0.709/0.709/0.729
III	0.368	0.781/0.727/0.727/0.770	0.782/0.731/0.731/0.770
III	0.607	0.781/0.754/0.754/0.834	0.782/0.758/0.758/0.834
III	1.000	0.781/0.787/0.780/0.920	0.782/0.789/0.781/0.922
III	1.649	0.781/0.941/0.783/0.998	0.782/0.941/0.783/0.999
III	2.718	0.781/1.985/0.781/1.181	0.782/1.985/0.782/1.180

**Table 6:** Sensitivity of relative estimation error to the ridge penalty value when cross-validation tuning is disabled. All four ridge penalties (Single-RR’s $\tau_{\mathrm{st}}$, Trans-RR’s $\tau_{1}$ and τ, Pooled-RR’s $\tau_{p}$) are forced to a common fixed value from {1/3,1,3,9}. Trans-RR-Ada’s mixing weight θ is still selected by 5-fold cross-validation on the target sample (Algorithm 2). All other settings are as in Figure 3, with M=500 replications per cell. Each entry reports Single-RR/Trans-RR/Trans-RR-Ada/Pooled-RR mean estimation error.

Case	*h*	τ = 1/3	τ = 1	τ = 3	τ = 9
I	0.135	0.742/0.769/0.687/0.619	0.628/0.523/0.525/0.605	0.742/0.620/0.620/0.773	0.881/0.814/0.814/0.906
I	0.223	0.742/0.781/0.696/0.645	0.628/0.538/0.538/0.625	0.742/0.631/0.632/0.783	0.881/0.820/0.820/0.910
I	0.368	0.742/0.804/0.709/0.694	0.628/0.565/0.561/0.661	0.742/0.651/0.652/0.801	0.881/0.829/0.829/0.917
I	0.607	0.742/0.851/0.728/0.790	0.628/0.616/0.596/0.726	0.742/0.687/0.690/0.833	0.881/0.846/0.846/0.929
I	1.000	0.742/0.953/0.745/0.990	0.628/0.714/0.629/0.848	0.742/0.752/0.740/0.890	0.881/0.876/0.876/0.951
I	1.649	0.742/1.185/0.743/1.423	0.628/0.913/0.628/1.074	0.742/0.866/0.742/0.986	0.881/0.924/0.881/0.986
I	2.718	0.742/1.739/0.742/2.321	0.628/1.287/0.628/1.436	0.742/1.033/0.742/1.113	0.881/0.987/0.881/1.030
II	0.135	2.026/2.443/2.014/1.143	0.978/0.964/0.930/0.789	0.861/0.778/0.784/0.850	0.924/0.877/0.878/0.936
II	0.223	2.026/2.459/2.019/1.167	0.978/0.979/0.938/0.804	0.861/0.787/0.793/0.857	0.924/0.881/0.882/0.939
II	0.368	2.026/2.490/2.027/1.211	0.978/1.005/0.953/0.833	0.861/0.803/0.810/0.870	0.924/0.888/0.889/0.943
II	0.607	2.026/2.551/2.037/1.297	0.978/1.055/0.972/0.885	0.861/0.832/0.836/0.894	0.924/0.900/0.903/0.952
II	1.000	2.026/2.681/2.045/1.475	0.978/1.152/0.988/0.983	0.861/0.885/0.865/0.937	0.924/0.923/0.922/0.968
II	1.649	2.026/2.971/2.042/1.848	0.978/1.342/0.984/1.161	0.861/0.977/0.864/1.008	0.924/0.959/0.927/0.994
II	2.718	2.026/3.615/2.034/2.569	0.978/1.663/0.978/1.420	0.861/1.096/0.861/1.093	0.924/1.000/0.925/1.022
III	0.135	1.286/1.442/1.246/0.827	0.784/0.713/0.704/0.686	0.798/0.693/0.694/0.808	0.902/0.844/0.844/0.920
III	0.223	1.286/1.456/1.253/0.852	0.784/0.729/0.715/0.705	0.798/0.703/0.704/0.817	0.902/0.849/0.849/0.923
III	0.368	1.286/1.484/1.265/0.900	0.784/0.756/0.735/0.737	0.798/0.721/0.724/0.833	0.902/0.857/0.857/0.929
III	0.607	1.286/1.541/1.282/0.994	0.784/0.808/0.763/0.797	0.798/0.754/0.759/0.861	0.902/0.872/0.873/0.940
III	1.000	1.286/1.661/1.294/1.187	0.784/0.909/0.789/0.910	0.798/0.815/0.799/0.912	0.902/0.898/0.899/0.959
III	1.649	1.286/1.933/1.290/1.600	0.784/1.109/0.786/1.115	0.798/0.920/0.799/0.996	0.902/0.941/0.903/0.990
III	2.718	1.286/2.559/1.286/2.420	0.784/1.466/0.784/1.426	0.798/1.064/0.798/1.103	0.902/0.993/0.902/1.026

**Table 7:** Prediction performance on the NIR spectral dataset over 20 repeated random splits. Each entry reports the average RMSE, with the standard deviation in parentheses.

Method	Direction A	Direction B
Trans-RR	4.6230 (0.1732)	4.7933 (0.2736)
Trans-RR-Ada	4.6294 (0.1861)	4.8211 (0.3757)
Pooled-RR	5.0952 (0.0812)	5.4909 (0.1335)
Trans-Lasso	5.5668 (0.3650)	5.6803 (0.3131)
Single-RR	6.2666 (2.2628)	6.8272 (2.2674)
Single-Lasso	8.0672 (2.8904)	8.5607 (3.3739)

## Data Availability

Publicly available datasets were analyzed in this study. The near-infrared spectral data are from the 2002 International Diffuse Reflectance Conference (IDRC) “Shootout” competition and can be downloaded from https://www.eigenvector.com/data/tablets/index.html, accessed on 7 May 2026. The code presented in this study is available on request from the corresponding author.

## Appendix A. Notation

For ease of reference, Table A1 collects the main symbols used throughout the paper, grouped by topic. Additional proof-level notation is introduced at the beginning of Appendix D.

**Table A1:** Summary of the main symbols used in the paper.

Symbol	Meaning
Dimensions and asymptotic regime	
*p*, *n*, $n_{1}$	predictor dimension; target and source sample sizes
$\kappa ,\kappa_{1}\in \left(0,\infty \right)$	limits of p/n and $p/n_{1}$
Target study (1) (i=1,…,n) and source study (2) ($i=1,\ldots ,n_{1}$)	
$x_{i}=\lambda_{i}X_{i}$, $x_{i}^{\left(1\right)}=\lambda_{i}^{\left(1\right)}X_{i}^{\left(1\right)}$	*i*-th target/source predictor, with components $X_{i},X_{i}^{\left(1\right)}\in R^{p}$ and scales $\lambda_{i},\lambda_{i}^{\left(1\right)}\in R$ (Assumptions 1(c) and 2(c))
$y_{i},\epsilon_{i}$, $y_{i}^{\left(1\right)},\epsilon_{i}^{\left(1\right)}$	target/source response and error ($\epsilon_{i}$ may be heavy-tailed)
$\beta_{0},w_{0}\in R^{p}$	target/source regression coefficient (non-sparse)
$\rho ,\psi =\rho^{\prime }$, $\tilde{\rho },\tilde{\psi }={\tilde{\rho }}^{\prime }$	target-stage/source-stage loss and its derivative
$\tau ,\tau_{st},\tau_{1}>0$	target-stage ridge for Trans-RR and for ${\hat{\beta }}_{st}$, source-stage ridge for $\hat{w}$
$\hat{w}$, $\hat{\delta }$, $\hat{\beta }=\hat{w}+\hat{\delta }$, ${\hat{\beta }}_{st}$	source-stage, Step 2, Trans-RR (5), and single-task (11) estimators
Source–target discrepancy	
$\delta_{0}=\beta_{0}-w_{0}$, $h=∥\delta_{0}∥$	discrepancy vector and its magnitude
Adaptive aggregation (Section 3.3)	
${\hat{\beta }}_{\mathrm{ada}}\left(\theta \right)=\theta \;\hat{\beta }+\left(1-\theta \right)\;{\hat{\beta }}_{st}$, ${\hat{\beta }}_{\mathrm{ada}}$	adaptive Trans-RR estimator and its instance at $\theta =\hat{\theta }$, (12)
θ∈[0,1], $\hat{\theta }$, Θ⊂[0,1]	mixing weight, its cross-validation choice, and candidate set
*K*, ${\left\{V_{k}\right\}}_{k=1}^{K}$, $L:R\to R_{\geq 0}$	fold count, target-sample fold partition, validation loss in (13)
Smoothed Huber family (choices for ρ and $\tilde{\rho }$)	
δ>0, η∈(0,δ]	Huber transition and smoothing parameters (scalar δ distinct from vector $\delta_{0}$)
$\rho_{H}$, $\rho_{\eta }$, $\rho_{P}$	Huber, smoothed Huber (6), and Pseudo-Huber (8) losses
Asymptotic quantities (Theorem 1)	
$r_{\rho }\left(\kappa \right),c_{\rho }\left(\kappa \right)$	asymptotic value of $∥\hat{\beta }-\beta_{0}∥$ and companion scalar in the fixed-point system
$Z_{i}$, $W_{i}=\epsilon_{i}+r_{\rho }\left(\kappa \right)\;\lambda_{i}Z_{i}$	standard normal (independent of $\epsilon_{i},\lambda_{i}$) and auxiliary random variable
prox(cρ)	proximal mapping: $\mathrm{prox}\left(c\rho \right)\left(x\right)={\mathrm{arg min}}_{y}\left\{c\rho \left(y\right)+{\left(x-y\right)}^{2}/2\right\}$

## Appendix B. Additional (*δ*, *η*) Heatmaps

This appendix reports the (δ,η) heatmaps at the six values of *h* not shown in Table 2 of the main text. The pattern at every *h* matches the one at h=1. Each method’s mean error varies by less than 7% across the nine (δ,η) pairs, and the four-method ranking matches the default (1.35,0.10) ranking in the large majority of cells. The few rank swaps that do occur happen at small *h* in Cases II and III, where Pooled-RR, Trans-RR, and Trans-RR-Ada have nearly tied mean errors.

**Table A2:** Sensitivity of relative estimation error to the smoothed Huber parameters (δ,η) at h=0.135. Each entry reports Single-RR/Trans-RR/Trans-RR-Ada/Pooled-RR mean estimation error over M=500 replications.

Case I (Gaussian Errors)			
δ∖η	0.05	0.10	0.20
1.00	0.653/0.560/0.561/0.604	0.654/0.561/0.562/0.604	0.656/0.562/0.563/0.606
1.35	0.644/0.549/0.550/0.600	0.645/0.550/0.550/0.600	0.646/0.551/0.551/0.601
2.00	0.641/0.537/0.538/0.594	0.640/0.537/0.539/0.594	0.641/0.538/0.539/0.593
Case II (Cauchy Errors)			
δ∖η	0.05	0.10	0.20
1.00	0.884/0.819/0.821/0.809	0.884/0.819/0.821/0.808	0.884/0.819/0.821/0.808
1.35	0.883/0.817/0.819/0.812	0.883/0.816/0.819/0.811	0.883/0.816/0.818/0.811
2.00	0.890/0.821/0.823/0.822	0.889/0.821/0.823/0.821	0.889/0.821/0.823/0.821
Case III (Mixture Errors)			
δ∖η	0.05	0.10	0.20
1.00	0.779/0.695/0.694/0.706	0.780/0.695/0.695/0.706	0.780/0.695/0.695/0.706
1.35	0.781/0.693/0.692/0.705	0.781/0.693/0.692/0.706	0.781/0.692/0.692/0.705
2.00	0.794/0.701/0.701/0.714	0.793/0.700/0.701/0.714	0.792/0.700/0.700/0.713

**Table A3:** Sensitivity of relative estimation error to the smoothed Huber parameters (δ,η) at h=0.223. Each entry reports Single-RR/Trans-RR/Trans-RR-Ada/Pooled-RR mean estimation error over M=500 replications.

Case I (Gaussian Errors)			
δ∖η	0.05	0.10	0.20
1.00	0.653/0.576/0.577/0.634	0.654/0.577/0.578/0.635	0.656/0.578/0.579/0.635
1.35	0.644/0.565/0.565/0.628	0.645/0.565/0.566/0.628	0.646/0.566/0.567/0.629
2.00	0.641/0.554/0.556/0.624	0.640/0.555/0.556/0.624	0.641/0.555/0.556/0.624
Case II (Cauchy Errors)			
δ∖η	0.05	0.10	0.20
1.00	0.884/0.828/0.830/0.824	0.884/0.828/0.830/0.825	0.884/0.828/0.831/0.825
1.35	0.883/0.826/0.829/0.829	0.883/0.826/0.829/0.829	0.883/0.825/0.828/0.828
2.00	0.890/0.829/0.832/0.838	0.889/0.830/0.832/0.837	0.889/0.829/0.831/0.836
Case III (Mixture Errors)			
δ∖η	0.05	0.10	0.20
1.00	0.779/0.709/0.709/0.729	0.780/0.709/0.709/0.729	0.780/0.710/0.709/0.730
1.35	0.781/0.708/0.707/0.728	0.781/0.707/0.707/0.729	0.781/0.707/0.706/0.728
2.00	0.794/0.715/0.715/0.737	0.793/0.715/0.715/0.737	0.792/0.714/0.714/0.736

**Table A4:** Sensitivity of relative estimation error to the smoothed Huber parameters (δ,η) at h=0.368. Each entry reports Single-RR/Trans-RR/Trans-RR-Ada/Pooled-RR mean estimation error over M=500 replications.

Case I (Gaussian Errors)			
δ∖η	0.05	0.10	0.20
1.00	0.653/0.598/0.600/0.683	0.654/0.599/0.601/0.684	0.656/0.601/0.602/0.686
1.35	0.644/0.588/0.589/0.678	0.645/0.588/0.589/0.678	0.646/0.589/0.591/0.678
2.00	0.641/0.579/0.580/0.677	0.640/0.579/0.580/0.677	0.641/0.579/0.580/0.677
Case II (Cauchy Errors)			
δ∖η	0.05	0.10	0.20
1.00	0.884/0.842/0.844/0.854	0.884/0.842/0.844/0.854	0.884/0.843/0.846/0.853
1.35	0.883/0.840/0.843/0.855	0.883/0.840/0.843/0.855	0.883/0.839/0.842/0.856
2.00	0.890/0.843/0.847/0.864	0.889/0.843/0.847/0.864	0.889/0.842/0.846/0.863
Case III (Mixture Errors)			
δ∖η	0.05	0.10	0.20
1.00	0.779/0.729/0.729/0.769	0.780/0.729/0.730/0.769	0.780/0.729/0.730/0.770
1.35	0.781/0.728/0.728/0.770	0.781/0.727/0.727/0.770	0.781/0.727/0.727/0.770
2.00	0.794/0.737/0.738/0.777	0.793/0.736/0.737/0.778	0.792/0.735/0.736/0.777

**Table A5:** Sensitivity of relative estimation error to the smoothed Huber parameters (δ,η) at h=0.607. Each entry reports Single-RR/Trans-RR/Trans-RR-Ada/Pooled-RR mean estimation error over M=500 replications.

Case I (Gaussian Errors)			
δ∖η	0.05	0.10	0.20
1.00	0.653/0.625/0.626/0.767	0.654/0.626/0.627/0.767	0.656/0.627/0.629/0.768
1.35	0.644/0.615/0.616/0.764	0.645/0.616/0.617/0.764	0.646/0.617/0.618/0.764
2.00	0.641/0.607/0.608/0.764	0.640/0.607/0.608/0.764	0.641/0.607/0.609/0.764
Case II (Cauchy Errors)			
δ∖η	0.05	0.10	0.20
1.00	0.884/0.862/0.864/0.897	0.884/0.862/0.864/0.897	0.884/0.863/0.865/0.897
1.35	0.883/0.860/0.863/0.898	0.883/0.860/0.862/0.898	0.883/0.859/0.862/0.898
2.00	0.890/0.865/0.868/0.903	0.889/0.864/0.868/0.903	0.889/0.864/0.868/0.902
Case III (Mixture Errors)			
δ∖η	0.05	0.10	0.20
1.00	0.779/0.755/0.755/0.835	0.780/0.755/0.755/0.835	0.780/0.755/0.755/0.836
1.35	0.781/0.754/0.754/0.834	0.781/0.754/0.754/0.834	0.781/0.753/0.754/0.834
2.00	0.794/0.764/0.764/0.841	0.793/0.763/0.764/0.840	0.792/0.762/0.762/0.840

**Table A6:** Sensitivity of relative estimation error to the smoothed Huber parameters (δ,η) at h=1.649. Each entry reports Single-RR/Trans-RR/Trans-RR-Ada/Pooled-RR mean estimation error over M=500 replications.

Case I(Gaussian Errors)			
δ∖η	0.05	0.10	0.20
1.00	0.653/0.797/0.653/0.987	0.654/0.795/0.654/0.987	0.656/0.796/0.657/0.987
1.35	0.644/0.792/0.645/0.990	0.645/0.791/0.645/0.990	0.646/0.792/0.646/0.990
2.00	0.641/0.788/0.641/0.999	0.640/0.789/0.641/0.999	0.641/0.787/0.641/0.998
Case II (Cauchy Errors)			
δ∖η	0.05	0.10	0.20
1.00	0.884/1.024/0.888/1.005	0.884/1.023/0.888/1.005	0.884/1.023/0.888/1.005
1.35	0.883/1.021/0.888/1.005	0.883/1.021/0.887/1.005	0.883/1.020/0.887/1.005
2.00	0.890/1.029/0.894/1.006	0.889/1.027/0.893/1.006	0.889/1.026/0.893/1.006
Case III (Mixture Errors)			
δ∖η	0.05	0.10	0.20
1.00	0.779/0.934/0.781/0.997	0.780/0.935/0.781/0.996	0.780/0.936/0.781/0.996
1.35	0.781/0.943/0.783/0.998	0.781/0.941/0.783/0.998	0.781/0.940/0.783/0.998
2.00	0.794/0.955/0.796/1.003	0.793/0.954/0.795/1.003	0.792/0.952/0.794/1.002

**Table A7:** Sensitivity of relative estimation error to the smoothed Huber parameters (δ,η) at h=2.718. Each entry reports Single-RR/Trans-RR/Trans-RR-Ada/Pooled-RR mean estimation error over M=500 replications.

Case I (Gaussian Errors)			
δ∖η	0.05	0.10	0.20
1.00	0.653/1.617/0.653/1.231	0.654/1.618/0.654/1.228	0.656/1.620/0.656/1.228
1.35	0.644/1.637/0.644/1.262	0.645/1.639/0.645/1.261	0.646/1.635/0.646/1.254
2.00	0.641/1.617/0.641/1.312	0.640/1.613/0.640/1.309	0.641/1.611/0.641/1.304
Case II (Cauchy Errors)			
δ∖η	0.05	0.10	0.20
1.00	0.884/1.882/0.884/1.118	0.884/1.882/0.884/1.119	0.884/1.890/0.884/1.118
1.35	0.883/1.902/0.883/1.122	0.883/1.898/0.883/1.123	0.883/1.900/0.883/1.121
2.00	0.890/1.873/0.890/1.137	0.889/1.869/0.889/1.139	0.889/1.864/0.889/1.136
Case III (Mixture Errors)			
δ∖η	0.05	0.10	0.20
1.00	0.779/1.975/0.779/1.169	0.780/1.971/0.780/1.171	0.780/1.993/0.780/1.169
1.35	0.781/1.992/0.781/1.183	0.781/1.985/0.781/1.181	0.781/1.971/0.781/1.178
2.00	0.794/1.999/0.794/1.209	0.793/2.001/0.793/1.208	0.792/2.009/0.792/1.205

## Appendix C. Robustness Checks for the Real-Data Analysis

In Section 5, we report robustness checks across two preprocessing variants: the four starting offsets of the every-fourth-wavelength thinning, and the same procedure without the whitening step (predictors still mean-centered per instrument). Table A8 reports the per-offset mean RMSE and standard deviation; Table A9 compares the whitened and the unwhitened analysis. Each entry reports the mean RMSE (with standard deviation in parentheses) over the 20 repeated random splits.

**Table A8:** Per-offset prediction performance on the NIR spectral dataset. The four offsets correspond to the four starting indices of the every-fourth-wavelength thinning, each yielding p=163 predictors.

Direction A: Target = *X*				
Method	Offset 0	Offset 1	Offset 2	Offset 3
Trans-RR	4.6230 (0.1732)	4.5555 (0.1619)	4.7143 (0.1660)	4.5511 (0.1149)
Trans-RR-Ada	4.6294 (0.1861)	4.5569 (0.1703)	4.7155 (0.1602)	4.5669 (0.1202)
Pooled-RR	5.0952 (0.0812)	4.9644 (0.1462)	5.0982 (0.0971)	4.9367 (0.0769)
Trans-Lasso	5.5668 (0.3650)	5.5799 (0.5213)	5.5239 (0.2604)	5.3689 (0.3004)
Single-RR	6.2666 (2.2628)	6.3039 (2.0647)	6.4540 (2.4723)	6.4131 (2.3509)
Single-Lasso	8.0672 (2.8904)	8.1289 (3.5097)	8.5205 (4.3280)	8.7014 (3.3436)
Direction B: Target = $X_{1}$				
Method	Offset 0	Offset 1	Offset 2	Offset 3
Trans-RR	4.7933 (0.2736)	4.7063 (0.1320)	4.6329 (0.2049)	4.6457 (0.1895)
Trans-RR-Ada	4.8211 (0.3757)	4.7405 (0.1403)	4.6222 (0.1583)	4.6447 (0.1779)
Pooled-RR	5.4909 (0.1335)	5.2180 (0.1444)	5.4916 (0.1550)	5.1584 (0.1728)
Trans-Lasso	5.6803 (0.3131)	5.9048 (0.5452)	5.5392 (0.4612)	5.6729 (0.3458)
Single-RR	6.8272 (2.2674)	6.9625 (2.5627)	6.6887 (2.2085)	6.6730 (2.4256)
Single-Lasso	8.5607 (3.3739)	9.0675 (3.7103)	7.8504 (2.7262)	7.9018 (2.8987)

**Table A9:** Comparison of mean RMSE between the whitened analysis (default) and the analysis without whitening, in both transfer directions.

	Direction A		Direction B	
Method	Whitened	Unwhitened	Whitened	Unwhitened
Trans-RR	4.6230 (0.1732)	4.6281 (0.2237)	4.7933 (0.2736)	4.9253 (0.2064)
Trans-RR-Ada	4.6294 (0.1861)	4.6225 (0.2182)	4.8211 (0.3757)	4.9386 (0.1926)
Pooled-RR	5.0952 (0.0812)	5.0615 (0.0959)	5.4909 (0.1335)	5.0178 (0.1401)
Trans-Lasso	5.5668 (0.3650)	4.8776 (0.3020)	5.6803 (0.3131)	5.0792 (0.2623)
Single-RR	6.2666 (2.2628)	4.8845 (0.3038)	6.8272 (2.2674)	5.2476 (0.3971)
Single-Lasso	8.0672 (2.8904)	4.9933 (0.3261)	8.5607 (3.3739)	5.2374 (0.3037)

## Appendix D. Assumptions

**Notation.** Let polyLog(p) replace a power of log(p). Denote by $I_{m}$ the m×m identity matrix. When this does not create problems, we also use the standard notation I for $I_{p}$. Let $0_{m}\in R^{m}$ and $1_{m}\in R^{m}$ be the vectors of zeros and ones, respectively. For an m×m matrix $A={\left\{a_{ij}\right\}}_{1\leq i,j\leq m}$, denote by $\lambda_{\max}\left(A\right)$ and $\lambda_{\min}\left(A\right)$ the maximum and minimum eigenvalues of A, respectively. The $L_{2}$ norm of A is defined as $∥A∥={\left\{\lambda_{\max}\left(A^{⊤}A\right)\right\}}^{1/2}$. We call $\hat{\sum }=n^{-1}\sum_{i=1}^{n}x_{i}x_{i}^{⊤}$ the sample covariance matrix of $x_{i}$’s when $x_{i}$’s are known to have zero mean. We say that X≤Y in $L_{k}$ if ${E(|X|}^{k}{)\leq E(|Y|}^{k})$. We use the notation (a,b) to represent either the interval (a,b) or (b,a), as in some cases we need to localize quantities within intervals defined by two values, *a* and *b*, without knowing in advance whether a<b or b>a. We denote by X the n×p design matrix whose *i*-th row is $x_{i}^{⊤}$, and by $X_{\left(i\right)}$ the matrix obtained by removing the *i*-th row of X. We write a∧b for min(a,b) and a∨b for max(a,b). For two symmetric matrices *A* and *B*, A⪰B means that A−B is positive semi-definite. For the random variable *W*, we use the definition ${∥W∥}_{L_{k}}={\left\{E(|W\right|}^{k}{)\}}^{1/k}$. For sequences of random variables $W_{n},Z_{n}$, we use the notation $W_{n}=O_{L_{k}}\left(Z_{n}\right)$ and $W_{n}=o_{L_{k}}\left(Z_{n}\right)$ when $∥W_{n}{∥}_{L_{k}}=O(∥Z_{n}{∥}_{L_{k}})$ and $∥W_{n}{∥}_{L_{k}}=o(∥Z_{n}{∥}_{L_{k}})$, respectively. For a vector $v={\left(v_{1},\ldots ,v_{m}\right)}^{⊤}$, the $L_{2}$ norms are $∥v∥={\left(\sum_{i=1}^{m}v_{i}^{2}\right)}^{1/2}$, whereas ${∥v∥}_{\infty }=\max_{1\leq k\leq p}\left|v\left(k\right)\right|$. For a function *f* from R to R, denote ${∥f∥}_{\infty }=\sup_{x\in R}\left|f\left(x\right)\right|$.

In this appendix, we refine the assumptions in the main text into the forms needed for the proof of Theorem 1. The proof is divided into three parts, and each part uses a slightly different set of technical conditions. In particular, the assumptions on $\beta_{0}$ are specified below according to the needs of the different proof subsections, whereas the source-side conditions remain as in Assumption 2.

Assumptions under which the whole proof goes through

- **M1.** p/n→κ∈(0,∞).
- **M2.** There exists constants $C_{\beta }$ and e>1/3 such that $∥\beta_{0}∥\leq C_{\beta }$ and $∥\beta_{0}{∥}_{\infty }=O\left(n^{-e}\right)$.
- **M3.** Suppose ρ is an even function. Assume that $\psi =\rho^{\prime }$ is bounded and $\psi^{\prime }$ is Lipschitz and bounded. Moreover, we assume that sign(ψ(x))=sign(x) and that ρ(x)≥ρ(0)=0 for all x∈R.
- **M4.** Assume that there exist independent variables $\lambda_{i}$’s and $X_{i}$’s such that $x_{i}=\lambda_{i}X_{i}$. Suppose that $X_{i}$’s are i.i.d. with independent entries, and they have mean $0_{p}$ and $\mathrm{cov}\left(X_{i}\right)=I_{p}$. Suppose there exist $c_{n}$ and $C_{n}$ that vary with *n*, where $1/c_{n}=O\left(\mathrm{polyLog}\left(n\right)\right)$ and $C_{n}$ is bounded in *n*, such that for any convex 1-Lipschitz function *G* of $X_{i}$, $P(|G\left(X_{i}\right)-m_{G}\left|>t\right)\leq C_{n}\exp\left(-c_{n}t^{2}\right)$ holds for all t>0, where $m_{G}$ is the median of $G(X_{i})$. We require the same assumption to hold for the columns of the n×p design matrix X. Additionally, we assume that the coordinates of $X_{i}$ have moments of all orders, and the *k*-th moment of the entries of $X_{i}$ is assumed to be uniformly bounded independently of *n* and *p* for all *k*. Also, for any 1≤k≤p, the vectors $\Theta_{k}=\left(X_{1}\left(k\right),\ldots ,X_{n}\left(k\right)\right)$ in $R^{n}$ satisfy: for any 1-Lipschitz (with respect to Euclidean norm) convex function *G*, if $m_{G(\Theta_{k})}$ is a median of $G(\Theta_{k})$, for any $t>0,P(|G\left(\Theta_{k}\right)-m_{G(\Theta_{k})}\left|>t\right)\leq C_{n}\exp\left(-c_{n}t^{2}\right)$, $C_{n}$ and $c_{n}$ can vary with *n*. As above, we assume that $1/c_{n}=O\left(\mathrm{polyLog}\left(n\right)\right)$.
- **M5.** $\lambda_{i}$’s are independent, with $E(\lambda_{i}^{2})=1$, $E(\lambda_{i}^{4})$ being bounded, and $\sup_{1\leq i\leq n}\left|\lambda_{i}\right|$ growing at most like $C_{\lambda }{\left(\log n\right)}^{k}$ for some *k*. $\lambda_{i}$’s may have finitely many possible distributions.
- **M6.** Suppose that $\epsilon_{i}$’s are independent and independent of $X_{i}$’s and $\lambda_{i}$’s. They may have finitely many possible distributions, each with a density that is differentiable, symmetric, and unimodal. Furthermore, for any r∈R, if z∼N(0,1), independent of $\epsilon_{i}$, $\epsilon_{i}+rz$ has a differentiable density $f_{i,r}$ which is increasing on (−∞,0) and decreasing on (0,∞). $\lim_{x\to \infty }xf_{i}\left(x\right)=0$.
- **M7.** $\epsilon_{i}$’s can have different distributions. Similarly, $\lambda_{i}$’s can have different distributions. The fraction of occurrences for each possible combination of distributions for $\left(\epsilon_{i},\lambda_{i}\right)$ has a limit as n→∞.

First part of the proof (Appendix E.3)

- **O1.** p/n→κ∈(0,∞).
- **O2.** ρ is twice differentiable, convex, and non-linear. $\psi =\rho^{\prime }$. Note that $\psi^{\prime }\geq 0$ since ρ is convex. We assume that sign(ψ(x))=sign(x) and ρ≥0=ρ(0).
- **O3.** $\sup_{x}\left|\psi \left(x\right)\right|\leq C\mathrm{polyLog}\left(n\right)$ and $∥\psi^{2}{∥}_{\infty }\leq C$ for some constant *C*. Furthermore, $\psi^{\prime }$ is assumed to be L(n)-Lipschitz with $L\left(n\right)\leq Cn^{\alpha },\alpha \geq 0$. We also assume that $∥\psi^{\prime }{∥}_{\infty }\leq C\mathrm{polyLog}\left(n\right)$.
- **O4.** Assume that there exist independent variables $\lambda_{i}$’s and $X_{i}$’s such that $x_{i}=\lambda_{i}X_{i}$. Suppose that $X_{i}$’s are i.i.d. with independent entries, and they have mean $0_{p}$ and $\mathrm{cov}\left(X_{i}\right)=I_{p}$. Suppose there exist $c_{n}$ and $C_{n}$ that vary with *n*, where $1/c_{n}=O\left(\mathrm{polyLog}\left(n\right)\right)$ and $C_{n}$ is bounded in *n*, such that for any convex 1-Lipschitz function *G* of $X_{i}$, $P(|G\left(X_{i}\right)-m_{G}\left|>t\right)\leq C_{n}\exp\left(-c_{n}t^{2}\right)$ holds for all t>0, where $m_{G}$ is the median of $G(X_{i})$. We require the same assumption to hold for the columns of the n×p design matrix X. Additionally, we assume that the coordinates of $X_{i}$ have moments of all orders, and the *k*-th moment of the entries of $X_{i}$ is assumed to be uniformly bounded independently of *n* and *p* for all *k*.
- **O5.** ${\left\{X_{i}\right\}}_{i=1}^{n}$ and ${\left\{\lambda_{i}\right\}}_{i=1}^{n}$ are independent of ${\left\{\epsilon_{i}\right\}}_{i=1}^{n}$. $\epsilon_{i}$’s are independent of each other.
- **O6.** $\sup_{1\leq i\leq n}\left|\lambda_{i}\right|≜L_{n}=O_{L_{k}}\left(\mathrm{polyLog}\left(n\right)\right)$ and $\lambda_{i}$ ’s are independent. Moreover, $E(\lambda_{i}^{2})=1$.
- **O7.** 1−2α>0 and $∥\beta_{0}∥=O\left(\mathrm{polyLog}\left(n\right)\right)$.

Second part of the proof (Appendix E.4)

- **P1.** $X_{i}$ ’s have independent entries. Furthermore, for any 1≤k≤p, the vectors $\Theta_{k}=\left(X_{1}\left(k\right),\ldots ,X_{n}\left(k\right)\right)$ in $R^{n}$ satisfy: for any 1-Lipschitz (with respect to Euclidean norm) convex function *G*, if $m_{G(\Theta_{k})}$ is a median of $G(\Theta_{k})$, for any $t>0,P(|G\left(\Theta_{k}\right)-m_{G(\Theta_{k})}\left|>t\right)\leq C_{n}\exp\left(-c_{n}t^{2}\right)$, $C_{n}$ and $c_{n}$ can vary with *n*. As above, we assume that $1/c_{n}=O\left(\mathrm{polyLog}\left(n\right)\right)$.
- **P2.** $∥\psi^{\prime }{∥}_{\infty }=O\left(1\right)$.
- **P3.** $∥\beta_{0}{∥}_{\infty }=O\left(n^{-e}\right)$, where e>0. Furthermore, $∥\beta_{0}{∥}_{2}\leq C$, where *C* is a constant independent of *p* and *n*. e satisfies α+1/4−e<0.
- **P4.** 1/2−2α>0 and min(1/2,e)−α−1/4>0. The latter implies that min(1/2,e)−α>0

Last part of the proof (Appendix E.5)

- **F1.** $\epsilon_{i}$’s may have different distributions; however, they may only come from finitely many distributions. Furthermore, for any r∈R, if z∼N(0,1), independent of $\epsilon_{i}$, $\epsilon_{i}+rz$ has a differentiable density $f_{i,r}$ which is increasing on (−∞,0) and decreasing on (0,∞). $\lim_{x\to \infty }xf_{i}\left(x\right)=0$.
- **F2.** ${∥\psi ∥}_{\infty }=O\left(1\right)$. $\psi^{\prime }$ has Lipschitz constant L(n). Furthermore, ${L\left(n\right)∥\psi ∥}_{\infty }=O\left(1\right)$.
- **F3.** α<1/6 and α+1/3<2min(1/2,e).
- **F4.** there exists constant *C* such that $E(\lambda_{i}^{4})\leq C$.
- **F5.** $\lambda_{i}$’s may have different distributions. The fraction of occurrences for each possible combination of distributions for $\left(\epsilon_{i},\lambda_{i}\right)$ has a limit as n→∞.

## Appendix E. Proof for Theorem 1

We call

$$F\left(\delta \right)=\frac{1}{n}\sum_{i=1}^{n}\rho \left\{\epsilon_{i}+x_{i}^{⊤}\left(w_{0}-\hat{w}\right)+x_{i}^{⊤}\left(\delta_{0}-\delta \right)\right\}+\frac{\tau }{2}{∥\delta ∥}^{2}.$$

$\hat{\delta }$ is defined as the solution of

$$\begin{array}{cc} f(\hat{\delta }) & =0\;\;\mathrm{with}\;\\ \nabla F=f(\delta ) & =\frac{1}{n}\sum_{i=1}^{n}-x_{i}\psi \left\{\epsilon_{i}+x_{i}^{⊤}\left(w_{0}-\hat{w}\right)+x_{i}^{⊤}\left(\delta_{0}-\delta \right)\right\}+\tau \delta . \end{array}$$

We further define

(A1) $${\tilde{\epsilon }}_{i}=\epsilon_{i}+x_{i}^{⊤}\left(w_{0}-\hat{w}\right),\;R_{i}={\tilde{\epsilon }}_{i}+x_{i}^{⊤}\left(\delta_{0}-\hat{\delta }\right),S=\frac{1}{n}\sum_{i=1}^{n}\psi^{\prime }\left(R_{i}\right)x_{i}x_{i}^{⊤},\;c_{\tau }=\frac{1}{n}\mathrm{tr}{\left(S+\tau I\right)}^{-1}.$$

### Appendix E.1. Preliminaries

**Lemma A1.** 
*Under Assumptions* ***P3*** *–* ***P4*** *, for any l=1,…,p, where w(l) denotes the lth coordinate of a vector w,*

$$|\hat{w}\left(l\right)-w_{0}\left(l\right)|=O_{L_{k}}\;\left(\mathrm{polyLog}\left(n_{1}\right)\;n_{1}^{-1/2}+n_{1}^{-e}\right)=O_{L_{k}}\;\left(\frac{\mathrm{polyLog}(n_{1})}{n_{1}^{1/2}\wedge n_{1}^{e}}\right).$$

*In particular, if we further assume $n=O(n_{1})$, we have*

$$|\hat{w}\left(l\right)-w_{0}\left(l\right)|=O_{L_{k}}\left(\frac{\mathrm{polyLog}(n)}{\sqrt{n}\wedge n^{e}}\right).$$

**Proof.** By Proposition 3.12 of El Karoui [21],

$$\left|w_{l}-w_{0}\left(l\right)\right|=O_{L_{k}}\;\left(\mathrm{polyLog}\left(n_{1}\right)\;n_{1}^{-1/2}+{∥w_{0}∥}_{\infty }\right),$$

where $w_{l}$ denotes the analog of $b_{p}$ defined in Appendix 4 of El Karoui [21]. Its explicit construction is rather involved and is not needed here; we only use $w_{l}$ as an intermediate quantity in the argument. Under Assumption P3, $∥w_{0}{∥}_{\infty }=O\left(n_{1}^{-e}\right)$, hence

(A2) $$\left|w_{l}-w_{0}\left(l\right)\right|=O_{L_{k}}\;\left(\mathrm{polyLog}\left(n_{1}\right)\;n_{1}^{-1/2}+n_{1}^{-e}\right)=O_{L_{k}}\;\left(\frac{\mathrm{polyLog}(n_{1})}{n_{1}^{1/2}\wedge n_{1}^{e}}\right).$$

Moreover, by Theorem 3.20 of El Karoui [21],

(A3) $$\hat{w}\left(l\right)-w_{l}=O_{L_{k}}\;\left(\frac{\mathrm{polyLog}\left(n_{1}\right)\;n_{1}^{\alpha }}{{\left[n_{1}^{1/2}\wedge n_{1}^{e}\right]}^{2}}\right).$$

Let m:=min(1/2,e). Then ${\left[n_{1}^{1/2}\wedge n_{1}^{e}\right]}^{2}=n_{1}^{2m}$ and

$$\frac{\mathrm{polyLog}\left(n_{1}\right)\;n_{1}^{\alpha }}{{\left[n_{1}^{1/2}\wedge n_{1}^{e}\right]}^{2}}=\mathrm{polyLog}\left(n_{1}\right)\;n_{1}^{-m}n_{1}^{\alpha -m}.$$

Assumption P4 gives m>α, thus

$$\frac{\mathrm{polyLog}\left(n_{1}\right)\;n_{1}^{\alpha }}{{\left[n_{1}^{1/2}\wedge n_{1}^{e}\right]}^{2}}=o\;\left(\mathrm{polyLog}\left(n_{1}\right)\;n_{1}^{-m}\right),$$

and hence (A3) yields

(A4) $$\hat{w}\left(l\right)-w_{l}=o_{L_{k}}\;\left(\mathrm{polyLog}\left(n_{1}\right)\;n_{1}^{-m}\right).$$

By the triangle inequality and Minkowski’s inequality in $L_{k}$,

$$∥\hat{w}\left(l\right)-w_{0}{\left(l\right)∥}_{L_{k}}\leq ∥\hat{w}\left(l\right)-w_{l}{∥}_{L_{k}}+{∥w_{l}-w_{0}\left(l\right)∥}_{L_{k}}.$$

Using (A2) and (A4), we obtain

$$∥\hat{w}\left(l\right)-w_{0}{\left(l\right)∥}_{L_{k}}=O\;\left(\frac{\mathrm{polyLog}(n_{1})}{n_{1}^{1/2}\wedge n_{1}^{e}}\right),$$

which is equivalent to

$$|\hat{w}\left(l\right)-w_{0}\left(l\right)|=O_{L_{k}}\;\left(\frac{\mathrm{polyLog}(n_{1})}{n_{1}^{1/2}\wedge n_{1}^{e}}\right).$$

Assume $n=O(n_{1})$, i.e., $n_{1}\geq n/C$ for some C>0 and all large *n*. Since polyLog(t) denotes a power of logt, write $\mathrm{polyLog}\left(t\right)={\left(\log t\right)}^{q}$ for some fixed q≥0 (up to a constant factor). Consider $f\left(t\right):={\left(\log t\right)}^{q}\;t^{-m}$. For t≥exp(q/m),

$$f^{\prime }\left(t\right)=\frac{{\left(\log t\right)}^{q-1}}{t^{m+1}}\left(q-m\log t\right)\leq 0,$$

so *f* is decreasing for all sufficiently large *t*. Hence, for all sufficiently large *n* and all $n_{1}\geq n/C$,

$$\frac{\mathrm{polyLog}(n_{1})}{\sqrt{n_{1}}\wedge n_{1}^{e}}=\frac{{\left(\log n_{1}\right)}^{q}}{n_{1}^{m}}=f\left(n_{1}\right)\leq f\left(n/C\right)=\frac{{\left(\log\left(n/C\right)\right)}^{q}}{{\left(n/C\right)}^{m}}\leq C^{m}\;\frac{{\left(\log n\right)}^{q}}{n^{m}}≍\frac{\mathrm{polyLog}(n)}{\sqrt{n}\wedge n^{e}},$$

where we use $a_{n}≍b_{n}$ to denote that $a_{n}=O\left(b_{n}\right)$ and $b_{n}=O\left(a_{n}\right)$. Therefore, we have

$$|\hat{w}\left(l\right)-w_{0}\left(l\right)|=O_{L_{k}}\;\left(\frac{\mathrm{polyLog}(n)}{\sqrt{n}\wedge n^{e}}\right).$$

□

**Proposition A1.** 

*Let $\delta_{1}$ and $\delta_{2}$ be the two vectors in $R^{p}$. Then, when ρ’s are convex and twice-differentiable,*

(A5)
$$∥\delta_{1}-\delta_{2}∥\leq \frac{1}{\tau }∥f\left(\delta_{1}\right)-f\left(\delta_{2}\right)∥.$$

**Proof.** We have by definition

$$f\left(\delta_{1}\right)-f\left(\delta_{2}\right)=\tau \left(\delta_{1}-\delta_{2}\right)+\frac{1}{n}\sum_{i=1}^{n}x_{i}\left[\psi \left\{{\tilde{\epsilon }}_{i}+x_{i}^{⊤}\left(\delta_{0}-\delta_{2}\right)\right\}-\psi \left\{{\tilde{\epsilon }}_{i}+x_{i}^{⊤}\left(\delta_{0}-\delta_{1}\right)\right\}\right].$$

By the mean value theorem, we have

$$\psi \left\{{\tilde{\epsilon }}_{i}+x_{i}^{⊤}\left(\delta_{0}-\delta_{2}\right)\right\}-\psi \left\{{\tilde{\epsilon }}_{i}+x_{i}^{⊤}\left(\delta_{0}-\delta_{1}\right)\right\}=\psi^{\prime }\left(\gamma_{i}^{🟉}\right)x_{i}^{⊤}\left(\delta_{0}-\delta_{1}\right),$$

where $\gamma_{i}^{🟉}$ is in the interval $\left({\tilde{\epsilon }}_{i}+x_{i}^{⊤}\left(\delta_{0}-\delta_{1}\right),{\tilde{\epsilon }}_{i}+x_{i}^{⊤}\left(\delta_{0}-\delta_{2}\right)\right)$. Hence,

$$\begin{array}{ccc} f\left(\delta_{1}\right)-f\left(\delta_{2}\right) & = & \tau \left(\delta_{1}-\delta_{2}\right)+\frac{1}{n}\sum_{i=1}^{n}\psi^{\prime }\left(\gamma_{i}^{🟉}\right)x_{i}x_{i}^{⊤}\left(\delta_{0}-\delta_{1}\right)\\ & = & \left(S_{\delta_{1},\delta_{2}}+\tau I_{p}\right)\left(\delta_{1}-\delta_{2}\right), \end{array}$$

where

$$S_{\delta_{1},\delta_{2}}=\frac{1}{n}\sum_{i=1}^{n}\psi^{\prime }\left(\gamma_{i}^{🟉}\right)x_{i}x_{i}^{⊤}.$$

This shows that

$$\delta_{1}-\delta_{2}={\left(S_{\delta_{1},\delta_{2}}+\tau I_{p}\right)}^{-1}\left\{f\left(\delta_{1}\right)-f\left(\delta_{2}\right)\right\}.$$

Since ρ is convex, $\psi^{\prime }=\rho^{\prime \prime }$ is non-negative and $S_{\delta_{1},\delta_{2}}$ is positive semi-definite. In the semi-definite order, we have $S_{\delta_{1},\delta_{2}}+\tau I_{p}⪰\tau I_{p}$. In particular,

$$∥\delta_{1}-\delta_{2}∥\leq \frac{1}{\tau }∥f\left(\delta_{1}\right)-f\left(\delta_{2}\right)∥.$$

□

Proposition A1 yields the following lemma.

**Lemma A2.** 

*For any $\delta_{1}$,*

$$∥\hat{\delta }-\delta_{1}∥\leq \frac{1}{\tau }∥f\left(\delta_{1}\right)∥.$$

The lemma is a simple consequence of Equation (A5) by definition $f(\hat{\delta })=0$.

### Appendix E.2. On $∥\hat{\delta }∥$ and $∥\hat{\delta }-\delta_{0}∥$

**Lemma A3.** 

*Define $q_{n}\left(b\right)=n^{-1}\sum_{i=1}^{n}x_{i}\psi \left\{{\tilde{\epsilon }}_{i}+x_{i}^{⊤}b\right\}$, $q_{n}\in R^{p}$.*

*If $D_{\psi }$ is the n×n diagonal matrix with (i,i)-entry $\psi \{{\tilde{\epsilon }}_{i}+x_{i}^{⊤}\delta_{0}\}$,*

$$∥\hat{\delta }∥\leq \frac{1}{\tau }∥q_{n}\left(\delta_{0}\right)∥=\frac{1}{\tau }\sqrt{\frac{1}{n^{2}}1^{⊤}D_{\psi }XX^{⊤}D_{\psi }1},$$

*and if $D_{\psi (\xi_{i})}$ is the n×n diagonal matrix with (i,i)-entry $\psi ({\tilde{\epsilon }}_{i})$,*

$$∥\hat{\delta }-\delta_{0}∥\;\leq \;∥\delta_{0}∥+\frac{1}{\tau }∥q_{n}\left(0\right)∥=∥\delta_{0}∥+\frac{1}{\tau }\sqrt{\frac{1}{n^{2}}1^{⊤}D_{\psi (\xi_{i})}XX^{⊤}D_{\psi (\xi_{i})}1},$$

*Also,*

$$∥q_{n}\left(\delta_{0}\right){∥}^{2}\leq \frac{1^{⊤}D_{\psi }^{2}1}{n}{∥XX^{⊤}/n∥}_{2},$$

*where ${∥A∥}_{2}$ denotes the largest singular value of the matrix A.*

*Therefore, under Assumptions **O1–O6*** *,*

$$\begin{array}{ccc} & & E(∥\hat{\delta }{∥}^{2})\leq \frac{1}{\tau^{2}}\frac{p}{n}C^{2}\mathrm{polyLog}\left(n\right),\\ & & E(∥\hat{\delta }{∥}^{4})\leq \frac{1}{\tau^{4}}C\mathrm{polyLog}\left(n\right). \end{array}$$

*Similarly, for any finite k,*

$$E(∥\hat{\delta }-\delta_{0}{∥}_{2}^{k})\leq C_{k}[∥\delta_{0}{∥}^{k}+\mathrm{polyLog}\left(n\right)/\tau^{k}].$$

*In the case k=2, we have the more precise bound*

$$E(∥\hat{\delta }-\delta_{0}{∥}^{2})\leq 2[∥\delta_{0}{∥}^{2}+\frac{p/n}{\tau^{2}}\frac{1}{n}\sum_{i=1}^{n}E\left\{\psi^{2}\left({\tilde{\epsilon }}_{i}\right)\right\}].$$

Noting that [21] has shown that $E(∥\hat{w}-w_{0}∥)=O\left(1\right)$, we have $E(∥\hat{\delta }+\hat{w}-\delta_{0}-w_{0}∥)$ is bounded by $K\mathrm{polyLog}\left(n\right)/\tau^{k}$.

**Proof.** Recall that $f\left(\delta \right)=\frac{1}{n}\sum_{i=1}^{n}-x_{i}\psi \left\{{\tilde{\epsilon }}_{i}+x_{i}^{⊤}\left(\delta_{0}-\delta \right)\right\}+\tau \delta$. Applying Lemma A2 with $\delta_{1}=0$ we have

$$∥\hat{\delta }-0∥\leq \frac{1}{\tau }∥f\left(0\right)∥=\frac{1}{\tau }∥\frac{1}{n}\sum_{i=1}^{n}-x_{i}\psi \left\{{\tilde{\epsilon }}_{i}+x_{i}^{⊤}\delta_{0}\right\}∥,$$

which gives the first inequality. Using $\delta_{1}=\delta_{0}$ we have

$$∥\hat{\delta }-\delta_{0}∥\leq \frac{1}{\tau }∥f\left(\delta_{0}\right)∥=\frac{1}{\tau }∥\frac{1}{n}\sum_{i=1}^{n}-x_{i}\psi \left({\tilde{\epsilon }}_{i}\right)+\tau \delta_{0}∥,$$

which gives the second inequality. We note that under our assumptions, according to Lemma 3.38 from [21], we have

$$∥XX^{⊤}{/n∥}_{2}=O_{L_{k}}\left(\mathrm{polyLog}\left(n\right)\right)$$

and

$$\frac{1}{n}\sum_{i=1}^{n}\psi^{2}\left\{{\tilde{\epsilon }}_{i}+x_{i}^{⊤}\delta_{0}\right\}\leq \frac{1}{n}\sum_{i=1}^{n}{∥\psi^{2}∥}_{\infty }=O\left(1\right),$$

which gives all the results about $L_{k}$ bounds. For the last result, we note that

$$∥q_{n}{\left(0\right)∥}^{2}=q_{n}{\left(0\right)}^{⊤}q_{n}\left(0\right)=\frac{1}{n^{2}}\sum_{i,j}x_{i}^{⊤}x_{j}\psi \left({\tilde{\epsilon }}_{i}\right)\psi \left({\tilde{\epsilon }}_{j}\right).$$

It implies that

$$E∥q_{n}{\left(0\right)∥}^{2}=\frac{1}{n^{2}}\sum_{i=1}^{n}E(∥x_{i}{∥}^{2})E\left\{\psi^{2}\left({\tilde{\epsilon }}_{i}\right)\right\}.$$

Because $E(∥x_{i}{∥}^{2})=p$, we can conclude that

$$E(∥q_{n}\left(0\right){∥}^{2})=\frac{p}{n}\frac{1}{n}\sum_{i=1}^{n}E\left\{\psi^{2}\left({\tilde{\epsilon }}_{i}\right)\right\}.$$

Together with the bound

$$∥\hat{\delta }-\delta_{0}{∥}^{2}\leq 2∥\delta_{0}{∥}^{2}+\frac{2}{\tau^{2}}{∥q_{n}\left(0\right)∥}^{2},$$

it implies the last result about k=2. □

### Appendix E.3. Leave-One-Observation-Out

In this subsection, we approximate $\hat{\delta }$ by ${\hat{\delta }}_{\left(i\right)}$ via leave-one-observation-out method.

We consider the situation where we leave the *i*-th observation, $\left(x_{i},\epsilon_{i}\right)$, out. By definition,

$${\hat{\delta }}_{\left(i\right)}=\underset{\delta \in R^{p}}{\mathrm{arg min}}F_{i}\left(\delta \right),\;\;\mathrm{where}\;F_{i}\left(\delta \right)=\frac{1}{n}\sum_{j\neq i}\rho_{j}\left\{{\tilde{\epsilon }}_{j}+x_{j}^{⊤}\delta_{0}-x_{j}^{⊤}\delta \right\}+\frac{\tau }{2}{∥\delta ∥}^{2}.$$

We call

$$\begin{array}{ccc} f_{i}\left(\delta \right) & = & -\frac{1}{n}\sum_{j\neq i}x_{j}\psi_{j}\left\{{\tilde{\epsilon }}_{j}+x_{j}^{⊤}\delta_{0}-x_{j}^{⊤}\delta \right\}+\tau \delta \\ & = & f\left(\delta \right)+\frac{1}{n}x_{i}\psi \left\{{\tilde{\epsilon }}_{i}+x_{i}^{⊤}\delta_{0}-x_{i}^{⊤}\delta \right\}. \end{array}$$

We have

$$f_{i}\left({\hat{\delta }}_{\left(i\right)}\right)=0.$$

We call

$${\tilde{r}}_{j,(i)}={\tilde{\epsilon }}_{j}-x_{j}^{⊤}\left({\hat{\delta }}_{\left(i\right)}-\delta_{0}\right)\;\mathrm{and}\;S_{i}=\frac{1}{n}\sum_{j\neq i}\psi_{j}^{\prime }\left({\tilde{r}}_{j,(i)}\right)x_{j}x_{j}^{⊤}.$$

Consider

$${\tilde{\delta }}_{i}={\hat{\delta }}_{\left(i\right)}+\frac{1}{n}{\left(S_{i}+\tau I\right)}^{-1}x_{i}\psi \left\{\mathrm{prox}\left(c_{i}\rho \right)\left({\tilde{r}}_{j,(i)}\right)\right\}≜{\hat{\delta }}_{\left(i\right)}+\eta_{i},$$

where

(A6) $$\begin{array}{ccc} & & c_{i}=\frac{1}{n}x_{i}^{⊤}{\left(S_{i}+\tau I\right)}^{-1}x_{i},\\ & & \eta_{i}=\frac{1}{n}{\left(S_{i}+\tau I\right)}^{-1}x_{i}\psi \left\{\mathrm{prox}\left(c_{i}\rho \right)\left({\tilde{r}}_{i,(i)}\right)\right\}. \end{array}$$

#### Appendix E.3.1. Deterministic Bounds

**Proposition A2.** 

*We have*

$$∥\hat{\delta }-{\tilde{\delta }}_{i}∥\leq \frac{1}{\tau }∥R_{i}∥,$$

*where*

$$R_{i}=\frac{1}{n}\sum_{j\neq i}\left[\psi_{j}^{\prime }\left\{\gamma^{★}\left(x_{j},{\hat{\delta }}_{\left(i\right)},\eta_{i}\right)\right\}-\psi_{j}^{\prime }\left({\tilde{r}}_{j,(i)}\right)\right]x_{j}x_{j}^{⊤}\eta_{i},$$

*and $\gamma^{★}\left(x_{j},{\hat{\delta }}_{\left(i\right)},\eta_{i}\right)$ is in the (“unordered”) interval $\left({\tilde{r}}_{j,(i)},{\tilde{r}}_{j,(i)}-x_{j}^{⊤}\eta_{i}\right)$.*

**Proof.** Recall that $y_{i}=\epsilon_{i}+x_{i}^{⊤}w_{0}+x_{i}^{⊤}\delta_{0}$. Since $f_{i}\left({\hat{\delta }}_{\left(i\right)}\right)=0$, and ${\tilde{\delta }}_{i}={\hat{\delta }}_{\left(i\right)}+\eta_{i}$, we have

$$\begin{array}{ccc} f({\tilde{\delta }}_{i}) & = & f\left({\tilde{\delta }}_{i}\right)-f_{i}\left({\hat{\delta }}_{\left(i\right)}\right)\\ & = & -\frac{1}{n}\sum_{i=1}^{n}x_{i}\psi \left\{{\tilde{\epsilon }}_{i}+x_{i}^{⊤}\delta_{0}-x_{i}^{⊤}{\tilde{\delta }}_{i}\right\}+\tau {\tilde{\delta }}_{i}+\frac{1}{n}\sum_{j\neq i}x_{j}\psi \left\{{\tilde{\epsilon }}_{j}+x_{j}^{⊤}\delta_{0}-x_{j}^{⊤}\delta_{\left(i\right)}\right\}-\tau \delta_{\left(i\right)}\\ & = & -\frac{1}{n}x_{i}\psi \left\{{\tilde{\epsilon }}_{i}+x_{i}^{⊤}\delta_{0}-x_{i}^{⊤}{\tilde{\delta }}_{i}\right\}+\tau \eta_{i}\\ & & +\frac{1}{n}\sum_{j\neq i}x_{j}\left[\psi_{j}\left\{{\tilde{\epsilon }}_{j}+x_{j}^{⊤}\delta_{0}-x_{j}^{⊤}{\hat{\delta }}_{\left(i\right)}\right\}-\psi_{j}\left\{{\tilde{\epsilon }}_{j}+x_{j}^{⊤}\delta_{0}-x_{j}^{⊤}\left({\hat{\delta }}_{\left(i\right)}+\eta_{i}\right)\right\}\right]. \end{array}$$

By the mean value theorem, we have

$$\begin{array}{ccc} & & \psi_{j}\left\{{\tilde{\epsilon }}_{j}+x_{j}^{⊤}\delta_{0}-x_{j}^{⊤}{\hat{\delta }}_{\left(i\right)}\right\}-\psi_{j}\left\{{\tilde{\epsilon }}_{j}+x_{j}^{⊤}\delta_{0}-x_{j}^{⊤}\left({\hat{\delta }}_{\left(i\right)}+\eta_{i}\right)\right\}\\ & & =\psi_{j}^{\prime }\left({\tilde{r}}_{j,(i)}\right)x_{j}^{⊤}\eta_{i}+\left[\psi_{j}^{\prime }\left\{\gamma^{★}\left(x_{j},{\hat{\delta }}_{\left(i\right)},\eta_{i}\right)\right\}-\psi_{j}^{\prime }\left({\tilde{r}}_{j,(i)}\right)\right]x_{j}^{⊤}\eta_{i}, \end{array}$$

where $\gamma^{★}\left(x_{j},{\hat{\delta }}_{\left(i\right)},\eta_{i}\right)$ is in the (“unordered”) interval $\left({\tilde{r}}_{j,(i)},{\tilde{r}}_{j,(i)}-x_{j}^{⊤}\eta_{i}\right)$. Therefore, if $R_{i}$ is defined as above, we have

$$\begin{array}{cc} \; & \frac{1}{n}\sum_{j\neq i}x_{j}\left[\psi_{j}\left\{{\tilde{\epsilon }}_{j}+x_{j}^{⊤}\delta_{0}-x_{j}^{⊤}{\hat{\delta }}_{\left(i\right)}\right\}-\psi_{j}\left\{{\tilde{\epsilon }}_{j}+x_{j}^{⊤}\delta_{0}-x_{j}^{⊤}\left({\hat{\delta }}_{\left(i\right)}+\eta_{i}\right)\right\}\right]\\ = & \frac{1}{n}\sum_{j\neq i}\psi_{j}^{\prime }\left({\tilde{r}}_{j,(i)}\right)x_{j}x_{j}^{⊤}\eta_{i}+R_{i}\\ = & S_{i}\eta_{i}+R_{i}. \end{array}$$

The previous simplicities yield that

$$f\left({\tilde{\delta }}_{i}\right)=-\frac{1}{n}x_{i}\psi \left\{{\tilde{\epsilon }}_{i}+x_{i}^{⊤}\delta_{0}-x_{i}^{⊤}{\tilde{\delta }}_{i}\right\}+\left(S_{i}+\tau I\right)\eta_{i}+R_{i}.$$

Since by definition, $\eta_{i}=n^{-1}{\left(S_{i}+\tau I\right)}^{-1}x_{i}\psi \left\{\mathrm{prox}\left(c_{i}\rho \right)\left({\tilde{r}}_{i,(i)}\right)\right\}$, we have

$$\left(S_{i}+\tau I\right)\eta_{i}=\frac{1}{n}x_{i}\psi \left\{\mathrm{prox}\left(c_{i}\rho \right)\left({\tilde{r}}_{i,(i)}\right)\right\}.$$

Also, by definition we have

$${\tilde{\epsilon }}_{i}+x_{i}^{⊤}\delta_{0}-x_{i}^{⊤}{\tilde{\delta }}_{i}={\tilde{r}}_{i,(i)}-c_{i}\psi \left\{\mathrm{prox}\left(c_{i}\rho \right)\left({\tilde{r}}_{i,(i)}\right)\right\}.$$

When ρ is differentiable, x−cψ{prox(cρ)(x)}=prox(cρ)(x). Therefore, ${\tilde{\epsilon }}_{i}+x_{i}^{⊤}\delta_{0}-x_{i}^{⊤}{\tilde{\delta }}_{i}=\mathrm{prox}\left(c_{i}\rho \right)\left({\tilde{r}}_{i,(i)}\right)$ and

$$-\frac{1}{n}x_{i}\psi \left\{{\tilde{\epsilon }}_{i}+x_{i}^{⊤}\delta_{0}-x_{i}^{⊤}{\tilde{\delta }}_{i}\right\}+\left(S_{i}+\tau I\right)\eta_{i}=-\frac{1}{n}x_{i}\left[\psi \left\{\mathrm{prox}\left(c_{i}\rho \right)\left({\tilde{r}}_{i,(i)}\right)\right\}-\psi \left({\tilde{r}}_{i,(i)}\right)\right]=0.$$

Therefore, $f\left({\tilde{\delta }}_{i}\right)=R_{i}$. Applying Lemma A2 we have

$$∥\hat{\delta }-{\tilde{\delta }}_{i}∥\leq \frac{1}{\tau }∥R_{i}∥.$$

□

i. On $R_{i}$

Next, we provide a bound for $R_{i}$.

**Lemma A4.** 

*We have*

$$∥\eta_{i}∥\leq \frac{1}{\sqrt{n}\tau }\frac{∥x_{i}∥}{\sqrt{n}}\left|\psi \left({\tilde{r}}_{i,(i)}\right)\right|,$$

*and*

$$∥R_{i}∥\leq ∥\hat{\sum }{∥}_{2}\underset{j\neq i}{\sup}|\psi_{j}^{\prime }\left\{\gamma^{★}\left(x_{j},{\hat{\delta }}_{\left(i\right)},\eta_{i}\right)\right\}-\psi_{j}^{\prime }\left({\tilde{r}}_{j,(i)}\right)|\frac{1}{\sqrt{n}\tau }\frac{∥x_{i}∥}{\sqrt{n}}\left|\psi \left({\tilde{r}}_{i,(i)}\right)\right|.$$

**Proof.** We have

$$R_{i}=\frac{1}{n}\sum_{j\neq i}\left[\psi_{j}^{\prime }\left\{\gamma^{★}\left(x_{j},{\hat{\delta }}_{\left(i\right)},\eta_{i}\right)\right\}-\psi_{j}^{\prime }\left({\tilde{r}}_{j,(i)}\right)\right]x_{j}x_{j}^{⊤}\eta_{i}.$$

Note that $S=n^{-1}\sum_{j\neq i}\left[\psi_{j}^{\prime }\left\{\gamma^{★}\left(x_{j},{\hat{\delta }}_{\left(i\right)},\eta_{i}\right)\right\}-\psi_{j}^{\prime }\left({\tilde{r}}_{j,(i)}\right)\right]x_{j}x_{j}^{⊤}$ can be written as $S=n^{-1}X^{⊤}DX$, where D is a diagonal matrix with (j,j)-entry $\left[\psi_{j}^{\prime }\left\{\gamma^{★}\left(x_{j},{\hat{\delta }}_{\left(i\right)},\eta_{i}\right)\right\}-\psi_{j}^{\prime }\left({\tilde{r}}_{j,(i)}\right)\right]$. Using the property of matrix norm ${∥\cdot ∥}_{2}$, we have ${∥S∥}_{2}\leq ∥\hat{\sum }{∥}_{2}{∥D∥}_{2}$, which implies that

$$∥R_{i}∥\leq ∥\hat{\sum }{∥}_{2}\underset{j\neq i}{\sup}|\psi_{j}^{\prime }\left\{\gamma^{★}\left(x_{j},{\hat{\delta }}_{\left(i\right)},\eta_{i}\right)\right\}-\psi_{j}^{\prime }\left({\tilde{r}}_{j,(i)}\right)|∥\eta_{i}∥,$$

where $\hat{\sum }=n^{-1}\sum_{j=1}^{n}x_{j}x_{j}^{⊤}$ is the sample covariance matrix. We now bound $∥\eta_{i}∥$. Note that

$$∥\eta_{i}∥\leq \frac{1}{\sqrt{n}\tau }\frac{∥x_{i}∥}{\sqrt{n}}\left|\psi \left\{\mathrm{prox}\left(c_{i}\rho \right)\left({\tilde{r}}_{i,(i)}\right)\right\}\right|.$$

Using Lemma A-1 in [25], we see that

$$|\psi \left({\mathrm{prox}}_{c_{i}}\left(\rho \right)\left({\tilde{r}}_{i,(i)}\right)\right)|\leq |\psi \left({\tilde{r}}_{i,(i)}\right)|.$$

□

Under our assumptions, we have

$$|\psi \left({\tilde{r}}_{i,(i)}\right){|\leq ∥\psi ∥}_{\infty }\leq C\mathrm{polyLog}\left(n\right)$$

and later in the proof of Lemma A6, we will show that

$$\underset{i}{\sup}\frac{∥x_{i}∥}{\sqrt{n}}=\underset{i}{\sup}\frac{|\lambda_{i}|∥X_{i}∥}{\sqrt{n}}=O_{L_{k}}(\underset{i}{\sup}|\lambda_{i}\left|\right).$$

ii. On $\gamma^{★}\left(x_{j},{\hat{\delta }}_{\left(i\right)},\eta_{i}\right)$ and related quantities

We now show how to control $n^{-1/2}\sup_{j\neq i}\left|\psi_{j}^{\prime }\left\{\gamma^{★}\left(x_{j},{\hat{\delta }}_{\left(i\right)},\eta_{i}\right)\right\}-\psi_{j}^{\prime }\left({\tilde{r}}_{j,(i)}\right)\right|$.

**Lemma A5.** 

*Suppose that $\psi^{\prime }$ is L(n)-Lipschitz. Then*

$$\underset{j\neq i}{\sup}|\psi_{j}^{\prime }\left\{\gamma^{★}\left(x_{j},{\hat{\delta }}_{\left(i\right)},\eta_{i}\right)\right\}-\psi_{j}^{\prime }\left({\tilde{r}}_{j,(i)}\right)|\leq L\left(n\right)\underset{j\neq i}{\sup}\left|x_{j}^{⊤}\eta_{i}\right|.$$

**Proof.** By definition, we have

$$|\gamma^{★}\left(x_{j},{\hat{\delta }}_{\left(i\right)},\eta_{i}\right)-{\tilde{r}}_{j,(i)}\left|\leq \right|x_{j}^{⊤}\eta_{i}|.$$

Therefore, the bound follows, using the fact that $\psi_{j}^{\prime }$ is L(n)-Lipschitz. □

#### Appendix E.3.2. Stochastic Aspects

Recall that

$$x_{j}^{⊤}\eta_{i}=\psi \left\{\mathrm{prox}\left(c_{i}\rho \right)\left({\tilde{r}}_{i,(i)}\right)\right\}\frac{1}{n}x_{j}^{⊤}{\left(S_{i}+\tau I\right)}^{-1}x_{i}.$$

Therefore, we can bound $∥R_{i}∥$ by

$$∥R_{i}∥\leq \left[\underset{j\neq i}{\sup}\frac{|x_{j}^{⊤}{\left(S_{i}+\tau I\right)}^{-1}x_{i}|}{n}\right]\frac{L(n)}{\sqrt{n}\tau }\frac{∥x_{i}∥}{\sqrt{n}}∥\hat{\sum }{∥}_{2}{∥\psi ∥}_{\infty }^{2}.$$

i. On $\sup_{j\neq i}\left|x_{j}^{⊤}{\left(S_{i}+\tau I\right)}^{-1}x_{i}\right|$

Now we control $x_{j}^{⊤}{\left(S_{i}+\tau I\right)}^{-1}x_{i}/n$.

**Lemma A6.** 
*Suppose $x_{i}$’s are independent and satisfy* ***O4*** *; suppose that $\lambda_{i}$’s satisfy* ***O6*** *. Then*

$$\underset{j\neq i}{\sup}\left|x_{j}^{⊤}{\left(S_{i}+\tau I\right)}^{-1}x_{i}/n\right|\leq \frac{1}{\sqrt{n}}\underset{j\neq i}{\sup}\frac{∥X_{j}∥}{\tau \sqrt{n}}\mathrm{polyLog}\left(n\right)$$

*in $L_{k}$, for any finite k. Note that under Assumption* ***O4*** *, for any finite k,*

$$\underset{j\neq i}{\sup}|∥X_{j}∥/\sqrt{n}|=O_{L_{k}}\left(1\right).$$

**Proof.** Note that

$$|x_{j}^{⊤}{\left(S_{i}+\tau I\right)}^{-1}x_{i}/n|=|\lambda_{i}\lambda_{j}\left|\right|X_{j}^{⊤}{\left(S_{i}+\tau I\right)}^{-1}X_{i}/n|.$$

Denote $X_{\left(i\right)}=\left(X_{1},\ldots ,X_{i-1},X_{i+1},\ldots ,X_{n}\right)$, and $v_{j,(i)}={\left(S_{i}+\tau I\right)}^{-1}X_{j}$. Then we have the map $F_{j}\left(X_{i}\right)=X_{j}^{⊤}{\left(S_{i}+\tau I\right)}^{-1}X_{i}=X_{i}^{⊤}v_{j,(i)}$ is linear in $X_{i}$ and it is Lipschitz with Lipschitz constant ${\left\{X_{j}^{⊤}{\left(S_{i}+\tau I\right)}^{-2}X_{j}\right\}}^{1/2}\leq ∥X_{j}∥/\tau$. Therefore, using Lemma B-2 in [25] and the fact that $X_{i}$ has mean 0, we have

$$\underset{j\neq i}{\sup}\left|X_{j}^{⊤}{\left(S_{i}+\tau I\right)}^{-1}X_{i}/n\right||X_{\left(i\right)}\leq \frac{1}{\sqrt{n}}\underset{j\neq i}{\sup}\frac{∥X_{j}∥}{\tau \sqrt{n}}\mathrm{polyLog}\left(n\right)/c_{n}^{1/2}$$

in $L_{k}$, when $\sup_{j\neq i}∥X_{j}∥/\left(\tau n^{1/2}\right)=O_{L_{k}}\left(1\right)$. To prove it, Using the fact that $X_{j}\to ∥X_{j}∥/n^{1/2}$ is $n^{-1/2}$-Lipschitz, we have

$$\underset{j\neq i}{\sup}|∥X_{j}∥/\sqrt{n}-m_{∥X_{j}∥/\sqrt{n}}|\leq \mathrm{polyLog}\left(n\right)/\sqrt{nc_{n}}\;\;\mathrm{in}\;\sqrt{L_{2k}}.$$

Note that $E(∥X_{j}∥)\leq \{E(∥X_{j}{∥}^{2}{)\}}^{1/2}=p^{1/2}$, so $m_{∥X_{j}∥/\sqrt{n}}$ is of order 1. Therefore, by Assumption **O4** on $c_{n}$ we have

$$\underset{j\neq i}{\sup}|∥X_{j}∥/\sqrt{n}|=O_{L_{k}}\left(1\right).$$

Now our Assumption O6 concerning $\sup_{i}\left|\lambda_{i}\right|=O_{L_{k}}\left(\mathrm{polyLog}\left(n\right)\right)$ guarantee that the bounds we announced are valid. □

Consequences

We have the following result.

**Proposition A3.** 
*Under Assumptions* ***O1–O6*** *, we have*

$$∥R_{i}∥=O_{L_{k}}\left(\frac{{\left[L\left(n\right)\right]∥\psi ∥}_{\infty }^{2}}{n\tau }\mathrm{polyLog}\left(n\right)\right).$$

*Furthermore, the same bound hold for $\sup_{1\leq i\leq n}∥R_{i}∥$.*

**Proof.** By aggregating all the intermediate results, using Holder’s inequality and the fact that $∥\hat{\sum }{∥}_{2}=O_{L_{k}}\left(\mathrm{polyLog}\left(n\right)\right)$ shown in Lemma 3.38 from [21], we finish the proof. □

We can now prove and state the following result. Recall that

$${\tilde{\delta }}_{i}={\hat{\delta }}_{\left(i\right)}+\frac{1}{n}{\left(S_{i}+\tau I_{p}\right)}^{-1}x_{i}\psi \left\{\mathrm{prox}\left(c_{i}\rho \right)\left({\tilde{r}}_{j,(i)}\right)\right\}≜{\hat{\delta }}_{\left(i\right)}+\eta_{i}.$$

**Theorem A1.** 
*Under Assumptions* ***O1–O7*** *, we have, for any fixed k, when τ is held fixed and $L\left(n\right)\leq Cn^{\alpha }$,*

$$\underset{1\leq i\leq n}{\sup}∥\hat{\delta }-{\tilde{\delta }}_{i}∥=O_{L_{k}}\left(\frac{\mathrm{polyLog}(n)}{n^{1-\alpha }}\right).$$

*In particular, we have*

$$\forall 1\leq i\leq n,E(∥\hat{\delta }-{\tilde{\delta }}_{i}{∥}^{2})=O\left(\frac{\mathrm{polyLog}(n)}{n^{2-2\alpha }}\right).$$

*Also,*

$$\underset{1\leq i\leq n}{\sup}\underset{j\neq i}{\sup}\left|{\tilde{r}}_{j,(i)}-R_{j}\right|=O_{L_{k}}\left(\frac{\mathrm{polyLog}(n)}{n^{1/2-\alpha }}\right).$$

*Finally,*

(A7)
$$\underset{1\leq i\leq n}{\sup}\left|R_{i}-\mathrm{prox}\left(c_{i}\rho \right)\left({\tilde{r}}_{i,(i)}\right)\right|=O_{L_{k}}\left(\frac{\mathrm{polyLog}(n)}{n^{1/2-\alpha }}\right).$$

**Proof.** The first two results are direct consequences of the previous propositions. The third result follows from that

$$\begin{array}{cc} \underset{j\neq i}{\sup}\left|{\tilde{r}}_{j,(i)}-R_{j}\right| & =\underset{j\neq i}{\sup}|x_{j}^{⊤}\left(\hat{\delta }-{\hat{\delta }}_{\left(i\right)}\right)|\leq \underset{j\neq i}{\sup}|x_{j}^{⊤}\left(\hat{\delta }-{\tilde{\delta }}_{i}\right)|+\underset{j\neq i}{\sup}\left|x_{j}^{⊤}\left({\tilde{\delta }}_{i}-{\hat{\delta }}_{\left(i\right)}\right)\right|\\ \; & =\left(\underset{1\leq j\leq n}{\sup}\frac{∥x_{j}∥}{\sqrt{n}}\right)\sqrt{n}∥\hat{\delta }-{\tilde{\delta }}_{i}∥+\underset{j\neq i}{\sup}\left|x_{j}^{⊤}\eta_{i}\right|, \end{array}$$

and the fact that $\sup_{1\leq j\leq n}∥x_{j}∥/n^{1/2}=O_{L_{k}}\left(\mathrm{polyLog}\left(n\right)\right)$ under our assumptions. Control of the first term follows from the results on $∥\hat{\delta }-{\tilde{\delta }}_{i}∥$. Control of the second term follows from Lemma A6 and the assumption that ψ is bounded by CpolyLog(n). For the last result, recall that

$$R_{i}={\tilde{\epsilon }}_{i}+x_{i}^{⊤}\left(\delta_{0}-\hat{\delta }\right)={\tilde{\epsilon }}_{i}+x_{i}^{⊤}\delta_{0}-x_{i}^{⊤}{\tilde{\delta }}_{i}-x_{i}^{⊤}\left(\hat{\delta }-{\tilde{\delta }}_{i}\right).$$

Given the definition of ${\tilde{\delta }}_{i}$, we have

$$x_{i}^{⊤}{\tilde{\delta }}_{i}=x_{i}^{⊤}{\hat{\delta }}_{\left(i\right)}+c_{i}\psi \left\{\mathrm{prox}\left(c_{i}\rho \right)\left({\tilde{r}}_{i,(i)}\right)\right\}.$$

By the property of the proximal operator, if y=prox(cρ)(x), y+cψ(y)=x, we have

$${\tilde{\epsilon }}_{i}+x_{i}^{⊤}\delta_{0}-x_{i}^{⊤}{\tilde{\delta }}_{i}={\tilde{r}}_{i,(i)}-c_{i}\psi \left[\mathrm{prox}\left(c_{i}\rho \right)\left({\tilde{r}}_{i,(i)}\right)\right]=\mathrm{prox}\left(c_{i}\rho \right)\left({\tilde{r}}_{i,(i)}\right).$$

Therefore, we have

$$\underset{i}{\sup}|R_{i}-\mathrm{prox}\left(c_{i}\rho \right)\left({\tilde{r}}_{i,(i)}\right)|=\underset{i}{\sup}\left|x_{i}^{⊤}\left(\hat{\delta }-{\tilde{\delta }}_{i}\right)\right|,$$

which is controlled in the previous results. □

On the limiting variance of $∥\hat{\delta }{∥}^{2}$ and $∥\hat{\delta }-\delta_{0}{∥}^{2}$

**Proposition A4.** 
*Under Assumptions* ***O1*** *–* ***O7*** *,*

$$\mathrm{var}(∥\hat{\delta }{∥}^{2})\to 0\;\mathrm{as}\;n\to \infty .$$

*Therefore, $∥\hat{\delta }{∥}^{2}$ has a deterministic equivalent in probability and in $L_{2}$.*
*More precisely, we have*

$$\mathrm{var}(∥\hat{\delta }{∥}^{2})=O\left(\frac{\mathrm{polyLog}(n)}{n^{1-2\alpha }}\right).$$

*The same type of results are true for $\mathrm{var}(∥\hat{\delta }-\delta_{0}{∥}^{2})$ and $\mathrm{var}(∥\hat{\beta }-\beta_{0}{∥}^{2})$ provided that $∥\delta_{0}∥=O\left(\mathrm{polyLog}\left(n\right)\right)$.*

**Proof.** We use the Efron–Stein inequality [35]: if *W* is a function of *n* independent random variables, and $W_{\left(i\right)}$ is any function of all those random variables except the *i*-th,

$$\mathrm{var}\left(W\right)\leq \sum_{i=1}^{n}\mathrm{var}\left(W-W_{\left(i\right)}\right)\leq \sum_{i=1}^{n}E\left({\left(W-W_{\left(i\right)}\right)}^{2}\right).$$

We apply this inequality to $W=∥\hat{\delta }{∥}^{2}$ and $W_{\left(i\right)}={∥{\hat{\delta }}_{\left(i\right)}∥}^{2}$. We first note that

$$E(|∥\hat{\delta }{∥}^{2}-∥{\hat{\delta }}_{\left(i\right)}{∥}^{2}{|}^{2})=2\left[E\left(|∥\hat{\delta }{∥}^{2}-∥{\tilde{\delta }}_{i}{{∥}^{2}|}^{2}\right)+E\left(|∥{\tilde{\delta }}_{i}{∥}^{2}-∥{\hat{\delta }}_{\left(i\right)}{{∥}^{2}|}^{2}\right)\right].$$

For the first term, we have $|∥\hat{\delta }{∥}^{2}-∥{\tilde{\delta }}_{i}{{∥}^{2}|}^{2}={\left[{\left(\hat{\delta }-{\tilde{\delta }}_{i}\right)}^{⊤}\left(\hat{\delta }+{\tilde{\delta }}_{i}\right)\right]}^{2}$ and ${\left(\hat{\delta }-{\tilde{\delta }}_{i}\right)}^{⊤}\left(\hat{\delta }+{\tilde{\delta }}_{i}\right)=2{\left(\hat{\delta }-{\tilde{\delta }}_{i}\right)}^{⊤}\hat{\delta }-{∥\hat{\delta }-{\tilde{\delta }}_{i}∥}^{2}$. Therefore, by the Cauchy–Schwarz inequality, we have

$$|∥\hat{\delta }{∥}^{2}-∥{\tilde{\delta }}_{i}{∥}^{2}{|}^{2}=O_{L_{1}}(∥\hat{\delta }-{\tilde{\delta }}_{i}{∥}^{4})+\sqrt{O_{L_{1}}\left(\mathrm{polyLog}\left(n\right)\right){∥\hat{\delta }-{\tilde{\delta }}_{i}∥}^{4}},$$

since $E(∥\hat{\delta }{∥}^{k})$ exists and is bounded by $k\mathrm{polyLog}\left(n\right)/\tau^{k}$. Using the results of Theorem A1 we have

$$E\left(|∥\hat{\delta }{∥}^{2}-∥{\tilde{\delta }}_{i}{{∥}^{2}|}^{2}\right)=O\left(\frac{\mathrm{polyLog}(n)}{n^{2-2\alpha }}\right)=o\left(n^{-1}\right),$$

provided that α<1/2. For the second term, by definition, we have

$$\begin{array}{cc} ∥{\tilde{\delta }}_{i}{∥}^{2}-{∥{\hat{\delta }}_{\left(i\right)}∥}^{2}= & \frac{2}{n}{\hat{\delta }}_{\left(i\right)}^{⊤}{\left(S_{i}+\tau I\right)}^{-1}x_{i}\psi \left(\mathrm{prox}\left(c_{i}\rho \right)\left({\tilde{r}}_{i,(i)}\right)\right)\\ \; & +\frac{1}{n^{2}}x_{i}^{⊤}{\left(S_{i}+\tau I\right)}^{-2}x_{i}\psi^{2}\left(\mathrm{prox}\left(c_{i}\rho \right)\left({\tilde{r}}_{i,(i)}\right)\right). \end{array}$$

Since $∥{\hat{\delta }}_{\left(i\right)}{∥}^{2}$ and $S_{i}$ are independent of $x_{i}$, and $∥{\left(S_{i}+\tau I\right)}^{-1}{∥}_{2}\leq \tau^{-1}$, we have ${\hat{\delta }}_{\left(i\right)}^{⊤}{\left(S_{i}+\tau I\right)}^{-1}x_{i}=O_{L_{2}}\left(\right|\lambda_{i}|∥{\hat{\delta }}_{\left(i\right)}∥/c_{n}^{1/2})$. Recall also that $\sup_{i}{∥\psi ∥}_{\infty }=O\left(\mathrm{polyLog}\left(n\right)\right)$. Therefore, both terms are of order $O_{L_{2}}\left(\mathrm{polyLog}\left(n\right)/nc_{n}^{1/2}\right)$. We can now conclude that

$$E\left(|∥{\tilde{\delta }}_{i}{∥}^{2}-∥{\hat{\delta }}_{\left(i\right)}{{∥}^{2}|}^{2}\right)=O\left(\frac{\mathrm{polyLog}(n)}{n^{2}}\right).$$

Therefore, we have

$$\mathrm{var}(∥\hat{\delta }{∥}^{2})=O\left(\frac{\mathrm{polyLog}(n)}{n^{1-2\alpha }}\right)=o\left(1\right).$$

This shows that $∥\hat{\delta }{∥}^{2}$ has a deterministic equivalent in probability and in $L_{2}$. For the second part of the result, similarly, we write that

$$E(|∥\hat{\delta }-\delta_{0}{∥}^{2}-∥{\hat{\delta }}_{\left(i\right)}-\delta_{0}{∥}^{2}{|}^{2})=2\left[E\left(|∥\hat{\delta }-\delta_{0}{∥}^{2}-∥{\tilde{\delta }}_{i}-\delta_{0}{{∥}^{2}|}^{2}\right)+E\left(|∥{\tilde{\delta }}_{i}-\delta_{0}{∥}^{2}-∥{\hat{\delta }}_{\left(i\right)}-\delta_{0}{{∥}^{2}|}^{2}\right)\right].$$

Using the fact that $|∥\hat{\delta }-\delta_{0}{∥}^{2}-∥{\tilde{\delta }}_{i}-\delta_{0}{{∥}^{2}|}^{2}={\left[{\left(\hat{\delta }-{\tilde{\delta }}_{i}\right)}^{⊤}\left(\hat{\delta }+{\tilde{\delta }}_{i}-2\delta_{0}\right)\right]}^{2}$ and ${\left(\hat{\delta }-{\tilde{\delta }}_{i}\right)}^{⊤}\left(\hat{\delta }+{\tilde{\delta }}_{i}-2\delta_{0}\right)=2{\left(\hat{\delta }-{\tilde{\delta }}_{i}\right)}^{⊤}\left(\hat{\delta }-\delta_{0}\right)-{∥\hat{\delta }-{\tilde{\delta }}_{i}∥}^{2}$, by the Cauchy–Schwarz inequality we have

$$|∥\hat{\delta }-\delta_{0}{∥}^{2}-∥{\tilde{\delta }}_{i}-\delta_{0}{∥}^{2}{|}^{2}=O_{L_{1}}(∥\hat{\delta }-{\tilde{\delta }}_{i}{∥}^{4})+\sqrt{O_{L_{1}}\left(\mathrm{polyLog}\left(n\right)\right){∥\hat{\delta }-{\tilde{\delta }}_{i}∥}^{4}},$$

since $E(∥\hat{\delta }-\delta_{0}{∥}^{k})$ exists and is bounded by $k\mathrm{polyLog}\left(n\right)/\tau^{k}$ following from Assumption **O7** and Lemma A3. Using the results of Theorem A1 we have

$$E\left(|∥\hat{\delta }-\delta_{0}{∥}^{2}-∥{\tilde{\delta }}_{i}-\delta_{0}{{∥}^{2}|}^{2}\right)=O\left(\frac{\mathrm{polyLog}(n)}{n^{2-2\alpha }}\right)=o\left(n^{-1}\right),$$

provided that α<1/2. Similarly, by definition we have

$$\begin{array}{cc} ∥{\tilde{\delta }}_{i}-\delta_{0}{∥}^{2}-{∥{\hat{\delta }}_{\left(i\right)}-\delta_{0}∥}^{2}= & \frac{2}{n}{\left({\hat{\delta }}_{\left(i\right)}-\delta_{0}\right)}^{⊤}{\left(S_{i}+\tau I\right)}^{-1}x_{i}\psi \left(\mathrm{prox}\left(c_{i}\rho \right)\left({\tilde{r}}_{i,(i)}\right)\right)\\ \; & +\frac{1}{n^{2}}x_{i}^{⊤}{\left(S_{i}+\tau I\right)}^{-2}x_{i}\psi^{2}\left(\mathrm{prox}\left(c_{i}\rho \right)\left({\tilde{r}}_{i,(i)}\right)\right). \end{array}$$

Since ${\left({\hat{\delta }}_{\left(i\right)}-\delta_{0}\right)}^{⊤}{\left(S_{i}+\tau I\right)}^{-1}x_{i}=O_{L_{2}}\left(\right|\lambda_{i}|∥{\hat{\delta }}_{\left(i\right)}-\delta_{0}∥/c_{n}^{1/2})$, both terms are of order $O_{L_{2}}\left(\mathrm{polyLog}\left(n\right)/nc_{n}^{1/2}\right)$. Similarly, we have

$$\mathrm{var}(∥\hat{\delta }-\delta_{0}{∥}^{2})=O\left(\frac{\mathrm{polyLog}(n)}{n^{1-2\alpha }}\right)=o\left(1\right).$$

The results for $\mathrm{var}(∥\hat{\beta }-\beta_{0}{∥}^{2})$ are simply followed by the fact that $\mathrm{var}(∥\hat{w}-w_{0}{∥}^{2})=o\left(1\right)$ in Proposition 3.10 of [21]. By assuming $\tilde{W}={∥\hat{\delta }+\hat{w}-\delta_{0}-w_{0}∥}^{2}$ and ${\tilde{W}}_{\left(i\right)}={∥{\hat{\delta }}_{\left(i\right)}+\hat{w}-\delta_{0}-w_{0}∥}^{2}$, we can similarly have

$$E\left(|∥\hat{\delta }+\hat{w}-\delta_{0}-w_{0}{∥}^{2}-∥{\hat{\delta }}_{\left(i\right)}+\hat{w}-\delta_{0}-w_{0}{{∥}^{2}|}^{2}\right)=o\left(1\right).$$

Note that $E(∥\hat{w}-w_{0}∥)=O\left(1\right)$, we have $E(∥\hat{\delta }+\hat{w}-\delta_{0}-w_{0}∥)$ is bounded by $K\mathrm{polyLog}\left(n\right)/\tau^{k}$. □

### Appendix E.4. Leaving Out a Predictor

Let V be the n×(p−1) matrix corresponding to the first (p−1) columns of the design matrix X. We call $v_{i}$ in $R^{p-1}$ the vector corresponding to the first p−1 entries of $x_{i}$, i.e $v_{i}^{⊤}=\left(x_{i}\left(1\right),\ldots ,x_{i}\left(p-1\right)\right)$. Denote $X_{i}={\left(X_{i}\left(1\right),\ldots ,X_{i}\left(p\right)\right)}^{⊤}$. We call X(p) the vector in $R^{n}$ with *j*-th entry $x_{j}\left(p\right)$, i.e the *p*-th entry of the vector $x_{j}$. When this does not create problems, we also use the standard notation $x_{j,p}$ for $x_{j}\left(p\right)$. We further denote $\delta_{0}={\left(\gamma_{0}^{⊤},\delta_{0}\left(p\right)\right)}^{⊤}$.

Call $\hat{\gamma }$ the solution of

(A8) $$\hat{\gamma }=\underset{\gamma \in R^{p-1}}{\mathrm{arg min}}\frac{1}{n}\sum_{i=1}^{n}\rho \left\{\epsilon_{i}+v_{i}^{⊤}\left(w_{0}-\hat{w}\right)-v_{i}^{⊤}\left(\gamma -\gamma_{0}\right)\right\}+\frac{\tau }{2}{∥\gamma ∥}^{2}.$$

Note that $\left[\begin{array}{c} \hat{\gamma }\\ 0 \end{array}\right]$ is the solution of the original optimization problem (5) when $x_{i}\left(p\right)$ is replaced by 0.

Approximation to $\hat{\delta }$ via leave-one-predictor-out

We use the notations and partitions

(A9) $$x_{i}=\left[\begin{array}{c} v_{i}\\ x_{i}\left(p\right) \end{array}\right],\;\hat{\delta }=\left[\begin{array}{c} {\hat{\delta }}_{-p}\\ \hat{\delta }\left(p\right) \end{array}\right].$$

Naturally, $\hat{\gamma }$ satisfies

(A10) $$-\frac{1}{n}\sum_{i=1}^{n}v_{i}\psi \left\{\epsilon_{i}+v_{i}^{⊤}\left(w_{0,-p}-{\hat{w}}_{-p}\right)-v_{i}^{⊤}\left(\hat{\gamma }-\gamma_{0}\right)\right\}+\tau \hat{\gamma }=0_{p-1}.$$

Denote

$$r_{i,[p]}=\epsilon_{i}+v_{i}^{⊤}\left(w_{0,-p}-{\hat{w}}_{-p}\right)-v_{i}^{⊤}\left(\hat{\gamma }-\gamma_{0}\right).$$

i.e., the residuals based on p−1 predictors.

Recall that

(A11) $$-\frac{1}{n}\sum_{i=1}^{n}x_{i}\psi \left\{\epsilon_{i}+x_{i}^{⊤}\left(w_{0}-\hat{w}\right)-x_{i}^{⊤}\left(\hat{\delta }-\delta_{0}\right)\right\}+\tau \hat{\delta }=0_{p},$$

and

$$R_{i}=\epsilon_{i}+x_{i}^{⊤}\left(w_{0}-\hat{w}\right)-x_{i}^{⊤}\left(\hat{\delta }-\delta_{0}\right).$$

Taking the difference between (A10) and (A11), we have

$$\frac{1}{n}\sum_{i}^{n}\left\{x_{i}\psi \left(R_{i}\right)-\left[\begin{array}{c} v_{i}\\ 0 \end{array}\right]\psi \left(r_{i,[p]}\right)\right\}-\tau \left(\hat{\delta }-\left[\begin{array}{c} \hat{\gamma }\\ 0 \end{array}\right]\right)=0_{p}.$$

Note that this *p*-dimensional equation can be separated into a scalar and a vector equation, namely,

$$\begin{array}{cc} \; & \frac{1}{n}\sum_{i}^{n}\left\{x_{i}\left(p\right)\psi \left(R_{i}\right)\right\}-\tau \hat{\delta }\left(p\right)=0,\\ \; & \frac{1}{n}\sum_{i}^{n}v_{i}\left\{\psi \left(R_{i}\right)-\psi \left(r_{i,[p]}\right)\right\}-\tau \left({\hat{\delta }}_{-p}-\hat{\gamma }\right)=0_{p-1}. \end{array}$$

Using a first-order Taylor expansion of $\psi (R_{i})$ around $\psi (r_{i,[p]})$ and noting that $R_{i}-r_{i,[p]}=v_{i}^{⊤}\left(\hat{\gamma }-{\hat{\delta }}_{-p}\right)+x_{i}\left(p\right)\left\{w_{0}\left(p\right)-\hat{w}\left(p\right)+\delta_{0}\left(p\right)-\hat{\delta }\left(p\right)\right\}$, we can transform the first equation above into

$$\frac{1}{n}\sum_{i}^{n}x_{i}\left(p\right)\left(\psi \left(r_{i,[p]}\right)+\psi^{\prime }\left(r_{i,[p]}\right)\left[v_{i}^{⊤}\left(\hat{\gamma }-{\hat{\delta }}_{-p}\right)+x_{i}\left(p\right)\left\{w_{0}\left(p\right)-\hat{w}\left(p\right)+\delta_{0}\left(p\right)-\hat{\delta }\left(p\right)\right\}\right]\right)-\tau \hat{\delta }\left(p\right)≃0.$$

This gives the near equivalence

$$\hat{\delta }\left(p\right)≃\frac{\frac{1}{n}\sum x_{i}\left(p\right)\left(\psi \left(r_{i,[p]}\right)+\psi^{\prime }\left(r_{i,[p]}\right)\left[v_{i}^{⊤}\left(\hat{\gamma }-{\hat{\delta }}_{-p}\right)+x_{i}\left(p\right)\left\{w_{0}\left(p\right)-\hat{w}\left(p\right)+\delta_{0}\left(p\right)\right\}\right]\right)}{\frac{1}{n}\sum x_{i}^{2}\left(p\right)\psi^{\prime }\left(r_{i,[p]}\right)+\tau }.$$

Working similarly on the second equation involving $v_{i}$, we have

$$\frac{1}{n}\sum_{i}^{n}\psi^{\prime }\left(r_{i,[p]}\right)v_{i}\left(R_{i}-r_{i,[p]}\right)-\tau \left({\hat{\delta }}_{-p}-\hat{\gamma }\right)≃0_{p-1}.$$

Since $R_{i}-r_{i,[p]}=v_{i}^{⊤}\left(\hat{\gamma }-{\hat{\delta }}_{-p}\right)+x_{i}\left(p\right)\left\{w_{0}\left(p\right)-\hat{w}\left(p\right)+\delta_{0}\left(p\right)-\hat{\delta }\left(p\right)\right\}$, the above equation can be transformed into

$$\left[\frac{1}{n}\sum_{i}^{n}\psi^{\prime }\left(r_{i,[p]}\right)v_{i}v_{i}^{⊤}\right]\left(\hat{\gamma }-{\hat{\delta }}_{-p}\right)+\left\{w_{0}\left(p\right)-\hat{w}\left(p\right)+\delta_{0}\left(p\right)-\hat{\delta }\left(p\right)\right\}\frac{1}{n}\sum_{i}^{n}\psi^{\prime }\left(r_{i,[p]}\right)v_{i}x_{i}\left(p\right)-\tau \left({\hat{\delta }}_{-p}-\hat{\gamma }\right)≃0_{p-1}.$$

Denote

$$u_{p}=\frac{1}{n}\sum_{i=1}^{n}\psi^{\prime }\left(r_{i,[p]}\right)v_{i}x_{i}\left(p\right),\;\mathrm{and}\;S_{p}=\frac{1}{n}\sum_{i=1}^{n}\psi^{\prime }\left(r_{i,[p]}\right)v_{i}v_{i}^{⊤},$$

we see that $\hat{\gamma }-{\hat{\delta }}_{-p}≃-\left\{w_{0}\left(p\right)-\hat{w}\left(p\right)+\delta_{0}\left(p\right)-\hat{\delta }\left(p\right)\right\}{\left(S_{p}+\tau I\right)}^{-1}u_{p}$. Using the above approximation in the equation for $\hat{\delta }\left(p\right)$, we can write

$$\hat{\delta }\left(p\right)≃\frac{\frac{1}{n}\sum x_{i}\left(p\right)\psi \left(r_{i,[p]}\right)+\left\{w_{0}\left(p\right)-\hat{w}\left(p\right)+\delta_{0}\left(p\right)\right\}\left\{\frac{1}{n}\sum x_{i}^{2}\left(p\right)\psi^{\prime }\left(r_{i,[p]}\right)-u_{p}^{⊤}{\left(S_{p}+\tau I\right)}^{-1}\right\}u_{p}}{\frac{1}{n}\sum x_{i}^{2}\left(p\right)\psi^{\prime }\left(r_{i,[p]}\right)-u_{p}^{⊤}{\left(S_{p}+\tau I\right)}^{-1}u_{p}+\tau }.$$

Denote

$$\xi_{n}≜\frac{1}{n}\sum_{i=1}^{n}x_{i}^{2}\left(p\right)\psi^{\prime }\left(r_{i,[p]}\right)-u_{p}^{⊤}{\left(S_{p}+\tau I\right)}^{-1}u_{p},$$

and

$$N_{p}≜\frac{1}{\sqrt{n}}\sum_{i=1}^{n}x_{i}\left(p\right)\psi \left(r_{i,[p]}\right).$$

We have

$$\left(\xi_{n}+\tau \right)\hat{\delta }\left(p\right)≃\sqrt{n}N_{p}+\left\{w_{0}\left(p\right)-\hat{w}\left(p\right)+\delta_{0}\left(p\right)\right\}\xi_{n}.$$

Also we have

$${\hat{\delta }}_{-p}≃\hat{\gamma }+\left\{w_{0}\left(p\right)-\hat{w}\left(p\right)+\delta_{0}\left(p\right)-\hat{\delta }\left(p\right)\right\}{\left(S_{p}+\tau I\right)}^{-1}u_{p}.$$

Thus, we construct an approximation to $\hat{\delta }$. As a summary, we introduce the following definitions:

**Definition A1.** 

*We call the residuals corresponding to this optimization problem ${\left\{r_{i,[p]}\right\}}_{i=1}^{n}$, in other words*

$$r_{i,[p]}=\epsilon_{i}+v_{i}^{⊤}\left(w_{0,-p}-{\hat{w}}_{-p}\right)-v_{i}^{⊤}\left(\hat{\gamma }-\gamma_{0}\right).$$

*We call*

$$u_{p}=\frac{1}{n}\sum_{i=1}^{n}\psi^{\prime }\left(r_{i,[p]}\right)v_{i}x_{i}\left(p\right),\;\mathrm{and}\;S_{p}=\frac{1}{n}\sum_{i=1}^{n}\psi^{\prime }\left(r_{i,[p]}\right)v_{i}v_{i}^{⊤}.$$

*Note that $u_{p}\in R^{p-1}$ and $S_{p}$ is (p−1)×(p−1). We call*

$$\xi_{n}≜\frac{1}{n}\sum_{i=1}^{n}x_{i}^{2}\left(p\right)\psi^{\prime }\left(r_{i,[p]}\right)-u_{p}^{⊤}{\left(S_{p}+\tau I\right)}^{-1}u_{p},$$

*and*

$$N_{p}≜\frac{1}{\sqrt{n}}\sum_{i=1}^{n}x_{i}\left(p\right)\psi \left(r_{i,[p]}\right).$$

*We consider*

$$b_{p}≜\left\{w_{0}\left(p\right)-\hat{w}\left(p\right)+\delta_{0}\left(p\right)\right\}\frac{\xi_{n}}{\tau +\xi_{n}}+\frac{1}{\sqrt{n}}\frac{N_{p}}{\tau +\xi_{n}}.$$

*Note that when $\xi_{n}>0$, we have*

$$b_{p}-\delta_{0}\left(p\right)=w_{0}\left(p\right)-\hat{w}\left(p\right)+\frac{n^{-1/2}N_{p}-\tau b_{p}}{\xi_{n}}.$$

*We call*

$$\tilde{b}=\left[\begin{array}{c} \hat{\gamma }\\ \delta_{0}\left(p\right)-\hat{w}\left(p\right)+w_{0}\left(p\right) \end{array}\right]+\left\{b_{p}-\delta_{0}\left(p\right)+\hat{w}\left(p\right)-w_{0}\left(p\right)\right\}\left[\begin{array}{c} -{\left(S_{p}+\tau I\right)}^{-1}u_{p}\\ 1 \end{array}\right].$$

#### Appendix E.4.1. Deterministic Aspects

**Proposition A5.** 

*We have*

(A12)
$$\begin{array}{c} ∥\hat{\delta }-\tilde{b}∥\leq \frac{1}{\tau }|b_{p}-\delta_{0}\left(p\right)+\hat{w}\left(p\right)-w_{0}\left(p\right)|\underset{1\leq i\leq n}{\sup}|d_{i,p}|∥\hat{\sum }{∥}_{2}\sqrt{∥{\left(S_{p}+\tau I\right)}^{-1}u_{p}{∥}^{2}+1} \end{array}$$

*where $d_{i,p}=\left[\psi^{\prime }\left(\gamma_{i,p}^{*}\right)-\psi^{\prime }\left(r_{i,[p]}\right)\right]$ and $\gamma_{i,p}^{*}$ is in the interval $\left(\epsilon_{i}+v_{i}^{⊤}\left(w_{0,-p}-{\hat{w}}_{-p}\right)-v_{i}^{⊤}\left(\hat{\gamma }-\gamma_{0}\right),\epsilon_{i}+x_{i}^{⊤}\left(w_{0}-\hat{w}\right)-x_{i}^{⊤}\left(\tilde{b}-\beta_{0}\right)\right)$. Furthermore,*

(A13)
$$∥{\left(S_{p}+\tau I\right)}^{-1}u_{p}{∥}^{2}\leq \frac{1}{n\tau }\sum_{i=1}^{n}x_{i}^{2}\left(p\right)\psi^{\prime }\left(r_{i,[p]}\right)=\frac{1}{n\tau }\sum_{i=1}^{n}\lambda_{i}^{2}\psi^{\prime }\left(r_{i,[p]}\right)X_{i}^{2}\left(p\right).$$

As in Lemma A2, we have

$$∥\hat{\delta }-\tilde{b}∥\leq \frac{1}{\tau }∥f\left(\tilde{b}\right)∥,$$

where

(A14) $$f\left(\tilde{b}\right)=-\frac{1}{n}\sum_{i=1}^{n}x_{i}\psi \left\{\epsilon_{i}+x_{i}^{⊤}\left(w_{0}-\hat{w}\right)-x_{i}^{⊤}\left(\tilde{b}-\delta_{0}\right)\right\}+\tau \tilde{b}.$$

We note furthermore that, by definition of $\hat{\gamma }$,

$$g\left(\hat{\gamma }\right)=-\frac{1}{n}\sum_{i=1}^{n}v_{i}\psi \left\{\epsilon_{i}+v_{i}^{⊤}\left(w_{0,-p}-{\hat{w}}_{-p}\right)-v_{i}^{⊤}\left(\hat{\gamma }-\gamma_{0}\right)\right\}+\tau \hat{\gamma }=0_{p-1}.$$

**Proof.** 
**i. Work on the first p−1 coordinates of $f(\tilde{b})$**
Denote $f_{p-1}\left(\delta \right)$ the first p−1 coordinates of f(δ). Denote ${\hat{\gamma }}_{ext}$ the *p*-dimensional vector whose first p−1 coordinates are $\hat{\gamma }$ and last coordinate is $\delta_{0}\left(p\right)$, i.e.,

$${\hat{\gamma }}_{ext}=\left[\begin{array}{c} \hat{\gamma }\\ \delta_{0}\left(p\right)-\hat{w}\left(p\right)+w_{0}\left(p\right) \end{array}\right].$$

For a vector v, we use the notation $v_{-k}$ to denote the vector obtained by removing the *k*-th coordinate of v. Note that

$$\begin{array}{ccc} f_{p-1}\left(\tilde{b}\right) & = & f_{p-1}\left(\tilde{b}\right)-g\left(\hat{\gamma }\right)\\ & = & -\frac{1}{n}\sum_{i=1}^{n}v_{i}\left[\psi \left\{\epsilon_{i}+x_{i}^{⊤}\left(w_{0}-\hat{w}\right)+x_{i}^{⊤}\left(\delta_{0}-\tilde{b}\right)\right\}-\psi \left\{\epsilon_{i}+v_{i}^{⊤}\left(w_{0,-p}-{\hat{w}}_{-p}\right)+v_{i}^{⊤}\left(\gamma_{0}-\hat{\gamma }\right)\right\}\right]\\ & & +\tau ({\tilde{b}}_{-p}-\hat{\gamma }). \end{array}$$

By the mean value theorem, for $\gamma_{i,p}^{*}$ in the interval $\left(\epsilon_{i}+v_{i}^{⊤}\left(w_{0}-\hat{w}\right)-v_{i}^{⊤}\left(w_{0,-p}-{\hat{w}}_{-p}\right),\epsilon_{i}+x_{i}^{⊤}\left(w_{0}-\hat{w}\right)-x_{i}^{⊤}\left(\tilde{b}-\delta_{0}\right)\right)$, we have

$$\begin{array}{ccc} & & \psi \left\{\epsilon_{i}+x_{i}^{⊤}\left(w_{0}-\hat{w}\right)+x_{i}^{⊤}\left(\delta_{0}-\tilde{b}\right)\right\}-\psi \left\{\epsilon_{i}+v_{i}^{⊤}\left(w_{0,-p}-{\hat{w}}_{-p}\right)+v_{i}^{⊤}\left(\gamma_{0}-\hat{\gamma }\right)\right\}\\ & = & \psi^{\prime }\left(\gamma_{i,p}^{*}\right)x_{i}^{⊤}\left({\hat{\gamma }}_{ext}-\tilde{b}\right)\\ & = & \psi^{\prime }\left(r_{i,[p]}\right)x_{i}^{⊤}\left({\hat{\gamma }}_{ext}-\tilde{b}\right)+\left\{\psi^{\prime }\left(\gamma_{i,p}^{*}\right)-\psi^{\prime }\left(r_{i,[p]}\right)\right\}x_{i}^{⊤}\left({\hat{\gamma }}_{ext}-\tilde{b}\right). \end{array}$$

Denote

$$\begin{array}{cc} d_{i,p} & =\psi^{\prime }\left(\gamma_{i,p}^{*}\right)-\psi^{\prime }\left(r_{i,[p]}\right),\\ \delta_{i,p} & =\left\{\psi^{\prime }\left(\gamma_{i,p}^{*}\right)-\psi^{\prime }\left(r_{i,[p]}\right)\right\}x_{i}^{⊤}\left({\hat{\gamma }}_{ext}-\tilde{b}\right),\\ R_{p} & =-\frac{1}{n}\sum_{i=1}^{n}v_{i}\left[\left\{\psi^{\prime }\left(\gamma_{i,p}^{*}\right)-\psi^{\prime }\left(r_{i,[p]}\right)\right\}x_{i}^{⊤}\left({\hat{\gamma }}_{ext}-\tilde{b}\right)\right]. \end{array}$$

Therefore, we have

$$f_{p-1}\left(\tilde{b}\right)=-\frac{1}{n}\sum_{i=1}^{n}\psi^{\prime }\left(r_{i,[p]}\right)v_{i}x_{i}^{⊤}\left({\hat{\gamma }}_{ext}-\tilde{b}\right)+\tau \left({\tilde{b}}_{-p}-\hat{\gamma }\right)+R_{p}≜A_{p}+R_{p}.$$

Note that by definition,

$$\begin{array}{cc} {\hat{\gamma }}_{ext}-\tilde{b} & =\left\{b_{p}-\delta_{0}\left(p\right)+\hat{w}\left(p\right)-w_{0}\left(p\right)\right\}\left[\begin{array}{c} {\left(S_{p}+\tau I\right)}^{-1}u_{p}\\ -1 \end{array}\right],\\ {\tilde{b}}_{-p}-\hat{\gamma } & =-\left\{b_{p}-\delta_{0}\left(p\right)+\hat{w}\left(p\right)-w_{0}\left(p\right)\right\}{\left(S_{p}+\tau I\right)}^{-1}u_{p}. \end{array}$$

Therefore, we have $x_{i}^{⊤}\left({\hat{\gamma }}_{ext}-\tilde{b}\right)=\left\{b_{p}-\delta_{0}\left(p\right)+\hat{w}\left(p\right)-w_{0}\left(p\right)\right\}\left\{v_{i}^{⊤}{\left(S_{p}+\tau I\right)}^{-1}u_{p}-x_{i}\left(p\right)\right\}$, and

$$A_{p}=-\left\{b_{p}-\delta_{0}\left(p\right)+\hat{w}\left(p\right)-w_{0}\left(p\right)\right\}\left[\frac{1}{n}\sum_{i=1}^{n}\psi^{\prime }\left(r_{i,[p]}\right)v_{i}\left\{v_{i}^{⊤}{\left(S_{p}+\tau I\right)}^{-1}u_{p}-x_{i}\left(p\right)\right\}+\tau {\left(S_{p}+\tau I\right)}^{-1}u_{p}\right].$$

By the definition of $S_{p}$ and $u_{p}$, we have

$$A_{p}=-\left\{b_{p}-\delta_{0}\left(p\right)+\hat{w}\left(p\right)-w_{0}\left(p\right)\right\}\left\{S_{p}{\left(S_{p}+\tau I\right)}^{-1}u_{p}-u_{p}+\tau {\left(S_{p}+\tau I\right)}^{-1}u_{p}\right\}=0_{p-1},$$

since $S_{p}{\left(S_{p}+\tau I\right)}^{-1}+\tau {\left(S_{p}+\tau I\right)}^{-1}=I$. Therefore, we conclude that

$$f_{p-1}\left(\tilde{b}\right)=R_{p}.$$

**ii. Work on the last coordinate of $f(\tilde{b})$**
Denote ${\left[f\left(\tilde{\delta }\right)\right]}_{p}$ the last coordinate of $f(\tilde{\delta })$. We have shown that

$$\begin{array}{ccc} & & \psi \left\{\epsilon_{i}+x_{i}^{⊤}\left(w_{0}-\hat{w}\right)+x_{i}^{⊤}\left(\delta_{0}-\tilde{b}\right)\right\}-\psi \left\{\epsilon_{i}+v_{i}^{⊤}\left(w_{0,-p}-{\hat{w}}_{-p}\right)+v_{i}^{⊤}\left(\gamma_{0}-\hat{\gamma }\right)\right\}\\ & = & \psi^{\prime }\left(r_{i,[p]}\right)x_{i}^{⊤}\left({\hat{\gamma }}_{ext}-\tilde{b}\right)+\left\{\psi^{\prime }\left(\gamma_{i,p}^{*}\right)-\psi^{\prime }\left(r_{i,[p]}\right)\right\}x_{i}^{⊤}\left({\hat{\gamma }}_{ext}-\tilde{b}\right). \end{array}$$

Recall that

$$\begin{array}{cc} r_{i,[p]} & =\epsilon_{i}+v_{i}^{⊤}\left(w_{0,-p}-{\hat{w}}_{-p}\right)-v_{i}^{⊤}\left(\hat{\gamma }-\gamma_{0}\right),\\ \delta_{i,p} & =\left\{\psi^{\prime }\left(\gamma_{i,p}^{*}\right)-\psi^{\prime }\left(r_{i,[p]}\right)\right\}x_{i}^{⊤}\left({\hat{\gamma }}_{ext}-\tilde{b}\right). \end{array}$$

We note that

$$\begin{array}{ccc} & & \psi \{\epsilon_{i}+x_{i}^{⊤}\left(w_{0}-\hat{w}\right)+x_{i}^{⊤}\left(\delta_{0}-\tilde{b}\right)\}\\ & = & \psi \left(r_{i,[p]}\right)+\psi^{\prime }\left(r_{i,[p]}\right)x_{i}^{⊤}\left({\hat{\gamma }}_{ext}-\tilde{b}\right)+\delta_{i,p}\\ & = & \psi \left(r_{i,[p]}\right)+\psi^{\prime }\left(r_{i,[p]}\right)\left\{b_{p}-\delta_{0}\left(p\right)+\hat{w}\left(p\right)-w_{0}\left(p\right)\right\}\left\{v_{i}^{⊤}{\left(S_{p}+\tau I\right)}^{-1}u_{p}-x_{i}\left(p\right)\right\}+\delta_{i,p}. \end{array}$$

Therefore, we have

$$\begin{array}{ccc} & & {\left[f\left(\tilde{\delta }\right)\right]}_{p}+\frac{1}{n}\sum_{i=1}^{n}x_{i}\left(p\right)\delta_{i,p}\\ & = & -\frac{1}{n}\sum_{i=1}^{n}x_{i}\left(p\right)\left[\psi \left(r_{i,[p]}\right)+\psi^{\prime }\left(r_{i,[p]}\right)\left\{b_{p}-\delta_{0}\left(p\right)+\hat{w}\left(p\right)-w_{0}\left(p\right)\right\}\left\{v_{i}^{⊤}{\left(S_{p}+\tau I\right)}^{-1}u_{p}-x_{i}\left(p\right)\right\}\right]+\tau \tilde{b}\left(p\right),\\ & = & -\frac{1}{n}\sum_{i=1}^{n}x_{i}\left(p\right)\psi \left(r_{i,[p]}\right)-\left\{b_{p}-\delta_{0}\left(p\right)+\hat{w}\left(p\right)-w_{0}\left(p\right)\right\}u_{p}^{⊤}{\left(S_{p}+\tau I\right)}^{-1}u_{p}\\ & & +\left\{b_{p}-\delta_{0}\left(p\right)+\hat{w}\left(p\right)-w_{0}\left(p\right)\right\}\frac{1}{n}\sum_{i=1}^{n}\psi^{\prime }\left(r_{i,[p]}\right)x_{i}^{2}\left(p\right)+\tau b_{p},\\ & = & -\left\{\frac{1}{n}\sum_{i=1}^{n}x_{i}\left(p\right)\psi \left(r_{i,[p]}\right)-\tau b_{p}\right\}\\ & & +\left\{b_{p}-\delta_{0}\left(p\right)+\hat{w}\left(p\right)-w_{0}\left(p\right)\right\}\left\{\frac{1}{n}\sum_{i=1}^{n}\psi^{\prime }\left(r_{i,[p]}\right)x_{i}^{2}\left(p\right)-u_{p}^{⊤}{\left(S_{p}+\tau I\right)}^{-1}u_{p}\right\},\\ & = & -\left(\frac{1}{\sqrt{n}}N_{p}-\tau b_{p}\right)+\left\{b_{p}-\delta_{0}\left(p\right)+\hat{w}\left(p\right)-w_{0}\left(p\right)\right\}\xi_{n},\\ & = & 0. \end{array}$$

We conclude that

$$\begin{array}{ccc} {\left[f\left(\tilde{\delta }\right)\right]}_{p} & = & -\frac{1}{n}\sum_{i=1}^{n}x_{i}\left(p\right)\delta_{i,p}\\ & = & -\frac{1}{n}\sum_{i=1}^{n}x_{i}\left(p\right)\left\{\psi^{\prime }\left(\gamma_{i,p}^{*}\right)-\psi^{\prime }\left(r_{i,[p]}\right)\right\}x_{i}^{⊤}\left({\hat{\gamma }}_{ext}-\tilde{b}\right). \end{array}$$

**iii. Representation of $f(\tilde{b})$**
Aggregating all the results we have obtained so far, we see that

(A15) $$\begin{array}{cc} f(\tilde{b})= & -\frac{1}{n}\sum_{i=1}^{n}d_{i,p}x_{i}x_{i}^{⊤}\left({\hat{\gamma }}_{ext}-\tilde{b}\right)\\ = & \left\{b_{p}-\delta_{0}\left(p\right)+\hat{w}\left(p\right)-w_{0}\left(p\right)\right\}\left\{\frac{1}{n}\sum_{i=1}^{n}d_{i,p}x_{i}x_{i}^{⊤}\right\}\left[\begin{array}{c} {\left(S_{p}+\tau I\right)}^{-1}u_{p}\\ -1 \end{array}\right], \end{array}$$

which implies (A12). For $∥{\left(S_{p}+\tau I\right)}^{-1}u_{p}{∥}^{2}$, denote $D_{\psi^{\prime }\left(r_{\cdot ,[p]}\right)}$ the diagonal matrix with (i,i) entry $\psi^{\prime }\left(r_{i,[p]}\right)$. We have

$$u_{p}=\frac{1}{n}V^{⊤}D_{\psi^{\prime }\left(r_{\cdot ,[p]}\right)}X\left(p\right).$$

Therefore,

$$\begin{array}{ccc} & & ∥{\left(S_{p}+\tau I\right)}^{-1}u_{p}{∥}^{2}\\ & = & \frac{X(p)}{\sqrt{n}}D_{\psi^{\prime }\left(r_{\cdot ,[p]}\right)}^{1/2}\frac{D_{\psi^{\prime }\left(r_{\cdot ,[p]}\right)}^{1/2}V}{\sqrt{n}}{\left(\frac{V^{⊤}D_{\psi^{\prime }\left(r_{\cdot ,[p]}\right)}V}{n}+\tau I\right)}^{-1}\frac{V^{⊤}D_{\psi^{\prime }\left(r_{\cdot ,[p]}\right)}^{1/2}}{\sqrt{n}}D_{\psi^{\prime }\left(r_{\cdot ,[p]}\right)}^{1/2}\frac{X(p)}{\sqrt{n}}. \end{array}$$

Note that

$$\frac{D_{\psi^{\prime }\left(r_{\cdot ,[p]}\right)}^{1/2}V}{\sqrt{n}}{\left(\frac{V^{⊤}D_{\psi^{\prime }\left(r_{\cdot ,[p]}\right)}V}{n}+\tau I\right)}^{-1}\frac{V^{⊤}D_{\psi^{\prime }\left(r_{\cdot ,[p]}\right)}^{1/2}}{\sqrt{n}}⪯I.$$

So we have

$$∥{\left(S_{p}+\tau I\right)}^{-1}u_{p}{∥}^{2}\leq \frac{1}{n}X{\left(p\right)}^{⊤}D_{\psi^{\prime }\left(r_{\cdot ,[p]}\right)}X\left(p\right)=\frac{1}{n}\sum_{i=1}^{n}x_{i}^{2}\left(p\right)\psi^{\prime }\left(r_{i,[p]}\right).$$

□

#### Appendix E.4.2. Stochastic Aspects

Assume that $X\left(p\right)={\left(X_{1}\left(p\right),\ldots ,X_{n}\left(p\right)\right)}^{⊤}$ is independent of ${\left\{V_{i},\epsilon_{i}\right\}}_{i=1}^{n}$, where $V_{i}=V_{i}/\lambda_{i}$. This is consistent with Assumption **P1**.

To bound $∥\hat{\delta }-\tilde{b}∥$, using Equation (A13) we have

$$∥{\left(S_{p}+\tau I\right)}^{-1}u_{p}{∥}^{2}\leq \frac{1}{n\tau }\sum_{i=1}^{n}\lambda_{i}^{2}{∥\psi^{\prime }∥}_{\infty }X_{i}^{2}\left(p\right),$$

and

$$∥{\left(S_{p}+\tau I\right)}^{-1}u_{p}{∥}^{2}\leq \frac{\sup_{i}{∥\psi^{\prime }∥}_{\infty }}{\tau }\frac{1}{n}\sum_{i=1}^{n}\lambda_{i}^{2}X_{i}^{2}\left(p\right).$$

Therefore, under Assumptions **O3**, **O4** and **O6**, we have for any fixed *k* and at fixed τ,

$$∥{\left(S_{p}+\tau I\right)}^{-1}u_{p}{∥}^{2}=O_{L_{k}}\left(\mathrm{polyLog}\left(n\right)\right).$$

It guarantees that

$${∥\begin{array}{c} {\left(S_{p}+\tau I\right)}^{-1}u_{p}\\ -1 \end{array}∥}^{2}\leq (1+∥{\left(S_{p}+\tau I\right)}^{-1}u_{p}{∥}^{2})=O_{L_{k}}\left(\mathrm{polyLog}\left(n\right)\right).$$

Thus

$$∥\hat{\delta }-\tilde{b}∥=O_{L_{k}}\left(\frac{1}{\tau }\mathrm{polyLog}\left(n\right)|b_{p}-\delta_{0}\left(p\right)+\hat{w}\left(p\right)-w_{0}\left(p\right)|\underset{1\leq i\leq n}{\sup}|d_{i,p}|∥\hat{\sum }{∥}_{2}\right).$$

Recall that Lemma 3.38 from [21] guarantees that $∥\hat{\sum }∥=O_{L_{k}}\left(\mathrm{polyLog}\left(n\right)\right)$ under assumption **O1–O7**. Also we will show in Proposition A6 that $|b_{p}-\delta_{0}\left(p\right)+\hat{w}\left(p\right)-w_{0}\left(p\right)|=O_{L_{k}}\left\{\mathrm{polyLog}\left(n\right)\left(n^{-1/2}\vee n^{-e}\right)\right\}$ and in Proposition A8 that $\sup_{1\leq i\leq n}\left|d_{i,p}\right|=\left\{\mathrm{polyLog}\left(n\right)\left(n^{\alpha -1/2}\vee n^{\alpha -e}\right)\right\}$ to show that $M_{1}$ is small.

On $b_{p}-\delta_{0}\left(p\right)$

Recall the notations

$$\begin{array}{cc} N_{p} & =\frac{1}{\sqrt{n}}\sum_{i=1}^{n}x_{i}\left(p\right)\psi \left(r_{i,[p]}\right)=\frac{1}{\sqrt{n}}\sum_{i=1}^{n}\lambda_{i}\psi \left(r_{i,[p]}\right)X_{i}\left(p\right),\\ \xi_{n} & =\frac{1}{n}\sum_{i=1}^{n}x_{i}^{2}\left(p\right)\psi^{\prime }\left(r_{i,[p]}\right)-u_{p}^{⊤}{\left(S_{p}+\tau I\right)}^{-1}u_{p}. \end{array}$$

Under our assumptions, we have $E\left(X_{i}\right)=0_{p}$ and $\mathrm{cov}\left(X_{i}\right)=I_{p}$, and X(p) is independent of ${\left\{r_{i,[p]}\right\}}_{i=1}^{n}$.

**Proposition A6.** 
*We have*

$$|b_{p}-\delta_{0}\left(p\right)+\hat{w}\left(p\right)-w_{0}\left(p\right)|\leq \frac{1}{\sqrt{n}\tau }|N_{p}|+∥\delta_{0}{∥}_{\infty }+\left|\hat{w}\left(p\right)-w_{0}\left(p\right)\right|.$$

*Furthermore, under Assumptions* ***O1–O7*** *and* ***P1*** *, $N_{p}=O_{L_{k}}\left(\mathrm{polyLog}\left(n\right)\right)$ and therefore, when τ is fixed,*

$$|b_{p}-\delta_{0}\left(p\right)+\hat{w}\left(p\right)-w_{0}\left(p\right)|=O_{L_{k}}(\mathrm{polyLog}\left(n\right)n^{-1/2}+∥\delta_{0}{∥}_{\infty }+|\hat{w}\left(p\right)-w_{0}\left(p\right)\left|\right).$$

Note that Lemma A1 has shown that

(A16) $$|\hat{w}\left(p\right)-w_{0}\left(p\right)|=O_{L_{k}}\left(\frac{\mathrm{polyLog}(n)}{\sqrt{n}\wedge n^{e}}\right).$$

Under Assumption **P3**, $n∥\delta_{0}{∥}_{\infty }^{2}\mathrm{polyLog}\left(n\right)n^{2\alpha -1/2}\to 0$, and therefore we have

(A17) $$|b_{p}-\delta_{0}\left(p\right)+\hat{w}\left(p\right)-w_{0}\left(p\right)|=O_{L_{k}}\left(\frac{\mathrm{polyLog}(n)}{\sqrt{n}\wedge n^{e}}\right).$$

**Proof.** From the definition of $b_{p}$, we have, when $\xi_{n}>0$,

$$b_{p}-\delta_{0}\left(p\right)+\hat{w}\left(p\right)-w_{0}\left(p\right)=\frac{1}{\sqrt{n}}\frac{N_{p}}{\tau +\xi_{n}}-\frac{\tau }{\tau +\xi_{n}}\left\{\delta_{0}\left(p\right)+w_{0}\left(p\right)-\hat{w}\left(p\right)\right\}.$$

We will see later that $\xi_{n}\geq 0$ in Lemma A7. It follows that

$$|b_{p}-\delta_{0}\left(p\right)+\hat{w}\left(p\right)-w_{0}\left(p\right)|\leq \frac{1}{\sqrt{n}\tau }|N_{p}\left|+\right|\delta_{0}\left(p\right)+w_{0}\left(p\right)-\hat{w}\left(p\right)|.$$

Using independence of X(p) and ${\left\{V_{i},\epsilon_{i}\right\}}_{i=1}^{n}$, and the fact that $E\left(X_{i}\right)=0_{p}$, we have

$$E\left(N_{p}^{2}\right)=\frac{1}{n}\sum_{i=1}^{n}E\left\{X_{i}^{2}\left(p\right)\right\}E\left\{\lambda_{i}^{2}\psi^{2}\left(r_{i,[p]}\right)\right\},$$

whether the right-hand side is finite or not. Using the bounds on $\max\lambda_{i}^{2}$ and $\sup_{i}{∥\psi ∥}_{\infty }$, we have

$$E\left(N_{p}^{2}\right)\leq \frac{1}{\sqrt{n}}\sum_{i=1}^{n}E\left\{X_{i}^{2}\left(p\right)\right\}{∥\psi ∥}_{\infty }E\left\{\lambda_{i}^{2}\right\}=O\left(1\right)=O\left(\mathrm{polyLog}\left(n\right)\right).$$

Similarly, it is clear that

$$N_{p}=O_{L_{k}}\left(\mathrm{polyLog}\left(n\right)\right).$$

Therefore, we have

$$|b_{p}-\delta_{0}\left(p\right)+\hat{w}\left(p\right)-w_{0}\left(p\right)|=\frac{1}{\sqrt{n}\tau }O_{L_{k}}\left(\mathrm{polyLog}\left(n\right)\right)+\underset{1\leq k\leq p}{\sup}|\delta_{0}\left(k\right)|+|\hat{w}\left(p\right)-w_{0}\left(p\right)|.$$

□

On $\xi_{n}$

Write $\xi_{n}$ in matrix form. Let $D_{\psi^{\prime }\left(r_{\cdot ,[p]}\right)}$ be the diagonal matrix with (i,i) entry $\psi^{\prime }\left(r_{i,[p]}\right)$. Denote X(p) the last column of the design matrix X. Then we have

$$\xi_{n}=\frac{1}{n}X{\left(p\right)}^{⊤}D_{\psi^{\prime }\left(r_{\cdot ,[p]}\right)}^{1/2}MD_{\psi^{\prime }\left(r_{\cdot ,[p]}\right)}^{1/2}X\left(p\right),$$

where

(A18) $$M=I_{n}-\frac{D_{\psi^{\prime }\left(r_{\cdot ,[p]}\right)}^{1/2}V}{\sqrt{n}}{\left(\frac{1}{n}V^{⊤}D_{\psi^{\prime }\left(r_{\cdot ,[p]}\right)}V+\tau I\right)}^{-1}\frac{V^{⊤}D_{\psi^{\prime }\left(r_{\cdot ,[p]}\right)}^{1/2}}{\sqrt{n}}.$$

**Lemma A7.** 

*We have*

$$\xi_{n}\geq 0.$$

*Furthermore, under Assumptions* ***O1–O7*** *and* ***P1*** *, if $D_{\lambda_{i}}$ is the diagonal matrix with (i,i) entry $\lambda_{i}$,*

$$|\xi_{n}-\frac{1}{n}\mathrm{tr}\left(D_{\lambda_{i}}D_{\psi^{\prime }\left(r_{\cdot ,[p]}\right)}^{1/2}MD_{\psi^{\prime }\left(r_{\cdot ,[p]}\right)}^{1/2}D_{\lambda_{i}}\right)|=O_{L_{k}}\left(\underset{1\leq i\leq n}{\sup}\lambda_{i}^{2}\psi^{\prime }\left(r_{i,[p]}\right)/\sqrt{nc_{n}}\right).$$

**Proof.** When τ>0, all the eigenvalues of M are positive. Indeed, if the singular values of $n^{1/2}D_{\psi^{\prime }\left(r_{\cdot ,[p]}\right)}^{1/2}V$ are denoted by $\sigma_{1}$, the eigenvalues of M are $\tau /(\sigma_{i}^{2}+\tau )$. Therefore, since $\xi_{n}=n^{-1}v^{⊤}Mv$ with $v=D_{\psi^{\prime }\left(r_{\cdot ,[p]}\right)}^{1/2}X\left(p\right)$, we have $\xi_{n}\geq 0$. Since M is symmetric and has eigenvalues between 0 and 1, using Lemma V.1.5 in [36], we have

$$0⪯D_{\psi^{\prime }\left(r_{\cdot ,[p]}\right)}^{1/2}MD_{\psi^{\prime }\left(r_{\cdot ,[p]}\right)}^{1/2}⪯D_{\psi^{\prime }\left(r_{\cdot ,[p]}\right)}.$$

Under Assumption **P1**, the matrix M is independent of X(p). $D_{\psi^{\prime }\left(r_{\cdot ,[p]}\right)}$ is also independent of X(p). By definition, $X\left(p\right)=D_{\lambda_{i}}X\left(p\right)$. Under Assumption **P1**, $X_{p}$ satisfy the necessary concentration assumptions. Using Lemma 3.37 in [21], we have

$$\begin{array}{cc} \; & \left|\frac{1}{n}X{\left(p\right)}^{⊤}D_{\psi^{\prime }\left(r_{\cdot ,[p]}\right)}^{1/2}MD_{\psi^{\prime }\left(r_{\cdot ,[p]}\right)}^{1/2}X\left(p\right)-\frac{1}{n}\mathrm{tr}\left(D_{\lambda_{i}}D_{\psi^{\prime }\left(r_{\cdot ,[p]}\right)}^{1/2}MD_{\psi^{\prime }\left(r_{\cdot ,[p]}\right)}^{1/2}D_{\lambda_{i}}\right)\right|\\ \; & =O_{L_{k}}\left(\frac{1}{\sqrt{nc_{n}}}\underset{1\leq i\leq n}{\sup}\lambda_{i}^{2}\psi^{\prime }\left(r_{i,[p]}\right)\right). \end{array}$$

□

About $\frac{1}{n}\mathrm{tr}\left(D_{\lambda_{i}}D_{\psi^{\prime }\left(r_{\cdot ,[p]}\right)}^{1/2}MD_{\psi^{\prime }\left(r_{\cdot ,[p]}\right)}^{1/2}D_{\lambda_{i}}\right)$

**Lemma A8.** 
*Denote*

$$S_{p}=\frac{1}{n}\sum_{i=1}^{n}\psi^{\prime }\left(r_{i,[p]}\right)v_{i}v_{i}^{⊤},\;\mathrm{and}\;\;S_{p}\left(i\right)=S_{p}-\frac{1}{n}\psi^{\prime }\left(r_{i,[p]}\right)v_{i}v_{i}^{⊤}.$$

*Denote*

(A19) $$\begin{array}{cc} c_{\tau ,p} & =\frac{1}{n}\mathrm{tr}\left\{{\left(S_{p}+\tau I\right)}^{-1}\right\},\\ \zeta_{i} & =\frac{1}{n}v_{i}^{⊤}{\left\{S_{p}\left(i\right)+\tau I\right\}}^{-1}v_{i}-\lambda_{i}^{2}c_{\tau ,p}. \end{array}$$

*Then we have under Assumptions* ***O1–O7*** *and* ***P1*** *, if M is the matrix defined in Lemma A7,*

$$|\frac{1}{n}\mathrm{tr}\left(I_{n}-M\right)-\left\{\frac{1}{n}\mathrm{tr}\left(D_{\lambda_{i}}D_{\psi^{\prime }\left(r_{\cdot ,[p]}\right)}^{1/2}MD_{\psi^{\prime }\left(r_{\cdot ,[p]}\right)}^{1/2}D_{\lambda_{i}}\right)\right\}c_{\tau ,p}|\leq \underset{i}{\sup}\left|\zeta_{i}\right|\cdot \frac{1}{n}\sum_{i=1}^{n}\psi^{\prime }\left(r_{i,[p]}\right).$$

*We also have*

$$\frac{1}{n}\mathrm{tr}\left(I_{n}-M\right)=\frac{p-1}{n}-\tau c_{\tau ,p}.$$

**Proof.** Denote $d_{i,i}=\psi^{\prime }\left(r_{i,[p]}\right)/n$. By using the Sherman-MorrisonWoodbury formula (see, e.g., [37], p. 19),

$$\begin{array}{cc} M_{i,i} & =1-d_{i,i}v_{i}^{⊤}{\left(V^{⊤}D_{\psi^{\prime }\left(r_{\cdot ,[p]}\right)}V/n+\tau I\right)}^{-1}v_{i},\\ \; & =1-d_{i,i}\frac{v_{i}^{⊤}{\left(S_{p}\left(i\right)+\tau I\right)}^{-1}v_{i}}{1+d_{i,i}v_{i}^{⊤}{\left(S_{p}\left(i\right)+\tau I\right)}^{-1}v_{i}},\\ \; & =\frac{1}{1+d_{i,i}v_{i}^{⊤}{\left(S_{p}\left(i\right)+\tau I\right)}^{-1}v_{i}}. \end{array}$$

Recall that we are interested in

$$\frac{1}{n}\sum_{i}\lambda_{i}^{2}\psi^{\prime }\left(r_{i,[p]}\right)M_{i,i}=\frac{1}{n}\left(D_{\lambda_{i}}D_{\psi^{\prime }\left(r_{\cdot ,[p]}\right)}^{1/2}MD_{\psi^{\prime }\left(r_{\cdot ,[p]}\right)}^{1/2}D_{\lambda_{i}}\right).$$

By the property of trace, we have

$$\begin{array}{cc} \mathrm{tr}(I_{n}-M) & =\mathrm{tr}\{{\left(S_{p}+\tau I\right)}^{-1}S_{p}\}\\ \; & =p-1-\tau \mathrm{tr}\left\{{\left(S_{p}+\tau I\right)}^{-1}\right\}=p-1-n\tau c_{\tau ,p}. \end{array}$$

This shows the second result of the lemma. The first result follows from the fact that

(A20) $$\begin{array}{cc} \mathrm{tr}(I_{n}-M) & =\sum_{i}\left(1-M_{i,i}\right)\\ & =\sum_{i}d_{i,i}\frac{v_{i}^{⊤}{\left(S_{p}\left(i\right)+\tau I\right)}^{-1}v_{i}}{1+d_{i,i}v_{i}^{⊤}{\left(S_{p}\left(i\right)+\tau I\right)}^{-1}v_{i}}. \end{array}$$

With our definitions, we have, since $\lambda_{i}^{2}c_{\tau ,p}+\zeta_{i}=n^{-1}v_{i}^{⊤}{\left(S_{p}\left(i\right)+\tau I\right)}^{-1}v_{i}$,

$$\begin{array}{cc} \frac{1}{n}\mathrm{tr}\left(I_{n}-M\right)= & \left(\frac{1}{n}\sum_{i}\lambda_{i}^{2}\psi^{\prime }\left(r_{i,[p]}\right)M_{i,i}\right)c_{\tau ,p}+\frac{1}{n}\sum_{i}\psi^{\prime }\left(r_{i,[p]}\right)\frac{\zeta_{i}}{1+d_{i,i}v_{i}^{⊤}{\left(S_{p}\left(i\right)+\tau I\right)}^{-1}v_{i}}. \end{array}$$

It immediately follows that

$$|\frac{1}{n}\mathrm{tr}\left(I_{n}-M\right)-\left(\frac{1}{n}\sum_{i}\lambda_{i}^{2}\psi^{\prime }\left(r_{i,[p]}\right)M_{i,i}\right)c_{\tau ,p}|\leq \underset{i}{\sup}\left|\zeta_{i}\right|\cdot \frac{1}{n}\sum_{i=1}^{n}\psi^{\prime }\left(r_{i,[p]}\right).$$

□

Controlling $\zeta_{i}$

**Lemma A9.** 

*Suppose we can find ${\left\{r_{i,[p]}^{\left(i\right)}\right\}}_{j\neq i}$ independent of $\left(\lambda_{i},V_{i}\right)$ and $K_{n}$ such that*

$$\underset{i}{\sup}\underset{j\neq i}{\sup}\left|\psi_{j}^{\prime }\left(r_{j,[p]}^{\left(i\right)}\right)-\psi_{j}^{\prime }\left(r_{j,[p]}\right)\right|\leq K_{n}.$$

*Then*

$$\underset{i}{\sup}\left|\zeta_{i}\right|=O_{L_{k}}\left[\left\{\frac{1}{\tau^{2}}K_{n}{∥\hat{\sum }∥}_{2}+\frac{\mathrm{polyLog}(n)}{\tau \sqrt{nc_{n}}}+\frac{1}{n\tau }\right\}\mathrm{polyLog}\left(n\right)\right],$$

*provided that $K_{n}$ has 3k uniformly bounded moments.*

**Proof.** Denote

$${\mathrm{AM}}_{i,p}=\frac{1}{n}\sum_{j\neq i}\psi^{\prime }\left(r_{j,[p]}^{\left(i\right)}\right)v_{j}v_{j}^{⊤}.$$

Then, using the fact that $A^{-1}-B^{-1}=A^{-1}\left(B-A\right)B^{-1}$, we have

$$∥{\left(S_{p}\left(i\right)+\tau I\right)}^{-1}-{\left({\mathrm{AM}}_{i,p}+\tau I\right)}^{-1}∥\leq \frac{1}{\tau^{2}}K_{n}{∥\hat{\sum }∥}_{2},$$

since $∥n^{-1}\sum_{i}v_{i}v_{i}^{⊤}∥\leq ∥\hat{\sum }{∥}_{2}$. In particular, we have

$$|\frac{1}{n}v_{i}^{⊤}{\left(S_{p}\left(i\right)+\tau I\right)}^{-1}v_{i}-\frac{1}{n}v_{i}^{⊤}{\left({\mathrm{AM}}_{i,p}+\tau I\right)}^{-1}v_{i}|\leq \frac{∥v_{i}{∥}^{2}}{n}\frac{1}{\tau^{2}}K_{n}{∥\hat{\sum }∥}_{2}.$$

Since ${\mathrm{AM}}_{i,p}$ is independent of $\left(\lambda_{i},V_{i}\right)$, we can use Lemma 3.37 in [21] and since $v=\lambda_{i}V_{i}$, we have

$$\underset{1\leq i\leq n}{\sup}|\frac{1}{n}v_{i}^{⊤}{\left({\mathrm{AM}}_{i,p}+\tau I\right)}^{-1}v_{i}-\frac{\lambda_{i}^{2}}{n}\mathrm{tr}\left\{{\left({\mathrm{AM}}_{i,p}+\tau I\right)}^{-1}\right\}|=O_{L_{k}}\left(\frac{\mathrm{polyLog}(n)}{\tau \sqrt{nc_{n}}}\underset{1\leq i\leq n}{\sup}\lambda_{i}^{2}\right),$$

by using the fact that $\lambda_{\max}\left({\left({\mathrm{AM}}_{i,p}+\tau I\right)}^{-1}\right)\leq \tau^{-1}$.
Using the operator norm bound we gave above, we also have

$$|\frac{1}{n}\mathrm{tr}\left\{{\left({\mathrm{AM}}_{i,p}+\tau I\right)}^{-1}\right\}-\frac{1}{n}\mathrm{tr}\left\{{\left(S_{p}\left(i\right)+\tau I\right)}^{-1}\right\}|\leq \frac{1}{\tau^{2}}K_{n}{∥\hat{\sum }∥}_{2}\frac{p}{n}.$$

We can now conclude that

$$\begin{array}{cc} \; & \underset{1\leq i\leq n}{\sup}|\frac{1}{n}v_{i}^{⊤}{\left(S_{p}\left(i\right)+\tau I\right)}^{-1}v_{i}-\frac{\lambda_{i}^{2}}{n}\mathrm{tr}\left\{{\left(S_{p}\left(i\right)+\tau I\right)}^{-1}\right\}|\\ \; & \;=O\left[\left\{\frac{1}{\tau^{2}}K_{n}{∥\hat{\sum }∥}_{2}\underset{1\leq i\leq n}{\sup}\left(\frac{p}{n}+\frac{∥v_{i}{∥}^{2}}{n}\right)+\frac{\mathrm{polyLog}(n)}{\tau \sqrt{nc_{n}}}\right\}\left(\underset{1\leq i\leq n}{\sup}\lambda_{i}^{2}\vee 1\right)\right]. \end{array}$$

So under **O1** and **O4**, $\sup_{1\leq i\leq n}{∥v_{i}∥}^{2}/n=O_{L_{k}}\left(1\right)$ and finally

$$\begin{array}{cc} \; & \underset{1\leq i\leq n}{\sup}|\frac{1}{n}v_{i}^{⊤}{\left(S_{p}\left(i\right)+\tau I\right)}^{-1}v_{i}-\frac{\lambda_{i}^{2}}{n}\mathrm{tr}\left\{{\left(S_{p}\left(i\right)+\tau I\right)}^{-1}\right\}|\\ \; & \;=O_{L_{k}}\left\{\left(\frac{1}{\tau^{2}}K_{n}{∥\hat{\sum }∥}_{2}+\frac{\mathrm{polyLog}(n)}{\tau \sqrt{nc_{n}}}\right)\left(\underset{1\leq i\leq n}{\sup}\lambda_{i}^{2}\vee 1\right)\right\}. \end{array}$$

Control of $n^{-1}\mathrm{tr}\left\{{\left(S_{p}\left(i\right)+\tau I\right)}^{-1}\right\}-n^{-1}\mathrm{tr}\left\{{\left(S_{p}+\tau I\right)}^{-1}\right\}$ Using the Sherman-Woodbury-Morrison formula, we have

$${\left(S_{p}\left(i\right)+\tau I\right)}^{-1}-{\left(S_{p}+\tau I\right)}^{-1}=\frac{\psi^{\prime }\left(r_{i,[p]}\right)}{n}\frac{{\left(S_{p}\left(i\right)+\tau I\right)}^{-1}v_{i}v_{i}^{⊤}{\left(S_{p}\left(i\right)+\tau I\right)}^{-1}}{1+\frac{\psi^{\prime }\left(r_{i,[p]}\right)}{n}v_{i}^{⊤}{\left(S_{p}\left(i\right)+\tau I\right)}^{-1}v_{i}}.$$

Take the trace of both sides, and we have

$$0\leq \mathrm{tr}\left\{{\left(S_{p}\left(i\right)+\tau I\right)}^{-1}\right\}-\mathrm{tr}\left\{{\left(S_{p}+\tau I\right)}^{-1}\right\}\leq \frac{1}{\tau },$$

since $v_{i}^{⊤}{\left(S_{p}\left(i\right)+\tau I\right)}^{-2}v_{i}\leq \tau^{-1}v_{i}^{⊤}{\left(S_{p}\left(i\right)+\tau I\right)}^{-1}v_{i}$. Therefore,

$$0\leq \frac{1}{n}\mathrm{tr}\left\{{\left(S_{p}\left(i\right)+\tau I\right)}^{-1}\right\}-\frac{1}{n}\mathrm{tr}\left\{{\left(S_{p}+\tau I\right)}^{-1}\right\}\leq \frac{1}{n\tau }.$$

We can now conclude that

$$\underset{i}{\sup}\left|\zeta_{i}\right|=O_{L_{k}}\left[\left\{\frac{1}{\tau^{2}}K_{n}{∥\hat{\sum }∥}_{2}+\frac{\mathrm{polyLog}(n)}{\tau \sqrt{nc_{n}}}+\frac{1}{n\tau }\right\}\left(\underset{1\leq i\leq n}{\sup}\lambda_{i}^{2}\vee 1\right)\right].$$

provided we can use Holder’s inequality. This, in turn, requires $K_{n}$ to have 3k moments. □

Control of $K_{n}$

A natural choice for ${\left\{r_{i,[p]}^{\left(i\right)}\right\}}_{j\neq i}$ defined in Lemma A9 is to use a leave-one-out estimator of $\hat{\gamma }$, where the *i*-th observation (and hence $v_{i}$) is removed. Hence, all the work performed in Theorem A1 can be used here.

**Lemma A10.** 

*Suppose we use for $r_{i,[p]}^{\left(i\right)}$ the residuals we would obtain by using a leave-one-out estimator of $\hat{\gamma }$, i.e., excluding $\left(v_{i},\epsilon_{i}\right)$ from problem (A8).*
*With the notations of Lemma A9, we have under Assumptions* ***O1–O7*** *and* ***P1*** *,*

$$K_{n}=O_{L_{k}}\left(n^{2\alpha -1/2}\mathrm{polyLog}\left(n\right)\right).$$

*In particular, for any fixed τ,*

$$\underset{i}{\sup}\left|\zeta_{i}\right|=O_{L_{k}}\left(n^{2\alpha -1/2}\mathrm{polyLog}\left(n\right)\right).$$

**Proof.** Denote $\delta_{n}\left(i\right)$ random variables such that

$$\underset{j\neq i}{\sup}\left|r_{j,[p]}^{\left(i\right)}-r_{j,[p]}\right|\leq \delta_{n}\left(i\right).$$

Applying Theorem A1 with $R_{j}=r_{j,[p]}$ and ${\tilde{r}}_{j,(i)}=r_{j,[p]}^{\left(i\right)}$, we have

$$\underset{i}{\sup}\delta_{n}\left(i\right)=O_{L_{k}}\left(n^{2\alpha -1/2}\mathrm{polyLog}\left(n\right)\right).$$

The control of $K_{n}$ follows by using the fact that $\psi^{\prime }$ is $Cn^{\alpha }$-Lipschitz. □

**Corollary A1.** 
*Recall that in (A6) and (A1)*

$$c_{i}=\frac{1}{n}x_{i}^{⊤}{\left(S_{i}+\tau I\right)}^{-1}x_{i},\;c_{\tau }=\frac{1}{n}\mathrm{tr}{\left(S+\tau I\right)}^{-1}.$$

*Then under Assumptions* ***O1–O7*** *and* ***P1*** *, we have*

$$\underset{i}{\sup}\left|c_{i}-\lambda_{i}^{2}c_{\tau }\right|=O_{L_{k}}\left(n^{2\alpha -1/2}\mathrm{polyLog}\left(n\right)\right).$$

**Proof.** We have shown that

$$\underset{i}{\sup}|\frac{1}{n}v_{i}^{⊤}{\left(S_{p}\left(i\right)+\tau I\right)}^{-1}v_{i}-\lambda_{i}^{2}c_{\tau ,p}|=O_{L_{k}}\left(n^{2\alpha -1/2}\mathrm{polyLog}\left(n\right)\right).$$

Recall that

$$c_{\tau }=\frac{1}{n}\mathrm{tr}\left[{\left\{\frac{1}{n}\sum_{i=1}^{n}\psi^{\prime }\left(R_{i}\right)x_{i}x_{i}^{⊤}+\tau I\right\}}^{-1}\right].$$

We see that $c_{\tau }$ is analogous to $c_{\tau ,p}$ when we use all the data, rather than (p−1) of them. Indeed, $c_{i}$ in (A6) is defined, in the notation of the proof of Lemma A9 as an analog of $n^{-1}v_{i}^{⊤}{\left({\mathrm{AM}}_{i,p}+\tau I\right)}^{-1}v_{i}$, with the role of ${\left\{r_{i,[p]}^{\left(i\right)}\right\}}_{j\neq i}$ being played by the residuals obtained by the leave-one-out estimator of $\hat{\gamma }$, excluding $\left(x_{i},y_{i}\right)$ from the problem. Lemma A9 in connection with Theorem A2 shows that $\sup_{i}\left|n^{-1}v_{i}^{⊤}{\left({\mathrm{AM}}_{i,p}+\tau I\right)}^{-1}v_{i}-\lambda_{i}^{2}c_{\tau ,p}\right|=O_{L_{k}}\left(n^{2\alpha -1/2}\mathrm{polyLog}\left(n\right)\right)$. Passing from the p−1 dimensional version of this result, i.e., Lemma A9, to the *p*-dimensional version gives the approximation stated in the corollary. We therefore have

$$\underset{i}{\sup}\left|c_{i}-\lambda_{i}^{2}c_{\tau }\right|=O_{L_{k}}\left(n^{2\alpha -1/2}\mathrm{polyLog}\left(n\right)\right).$$

□

Further results on $\xi_{n}$ and $b_{p}$

**Proposition A7.** 
*Under Assumptions* ***O1–O7*** *and* ***P1*** *, we have*

(A21) $$|c_{\tau ,p}\left(\xi_{n}+\tau \right)-\frac{p-1}{n}|=O_{L_{k}}\left(\frac{\mathrm{polyLog}(n)}{n^{1/2-2\alpha }}\right).$$

*Furthermore, under Assumptions* ***O1–O7*** *and* ***P1–P3*** *, since $∥\delta_{0}{∥}_{\infty }=O_{L_{k}}\left(n^{-e}\right)$,*

(A22) $$\begin{array}{cc} {\left(\frac{p}{n}\right)}^{2}nE\left[{\left\{b_{p}-\delta_{0}\left(p\right)+\hat{w}\left(p\right)-w_{0}\left(p\right)\right\}}^{2}\right]= & \frac{1}{n}\sum_{i=1}^{n}E\left[{\left\{c_{\tau ,p}\lambda_{i}\psi \left(r_{i,[p]}\right)\right\}}^{2}\right]\\ \; & +n\tau^{2}{\left\{\delta_{0}\left(p\right)-\hat{w}\left(p\right)+w_{0}\left(p\right)\right\}}^{2}E\left(c_{\tau ,p}^{2}\right)+o\left(1\right). \end{array}$$

**Proof.**  **For Equation** (A21): By Lemma A8, we have

$$\frac{p-1}{n}-\tau c_{\tau ,p}=\frac{1}{n}\mathrm{tr}\left(I_{n}-M\right)\geq 0.$$

The latter quantity was approximated in Lemma A8 by

$$\frac{1}{n}\mathrm{tr}\left(D_{\lambda_{i}}D_{\psi^{\prime }\left(r_{\cdot ,[p]}\right)}^{1/2}MD_{\psi^{\prime }\left(r_{\cdot ,[p]}\right)}^{1/2}D_{\lambda_{i}}\right)c_{\tau ,p},$$

which approximate ξ as in Lemma A7. This gives the result of Equation (A21), by simply keeping track of the approximation errors we make at each step. **For Equation** (A22): Recall that by definition:

$$\sqrt{n}\left[\left(\tau +\xi_{n}\right)b_{p}-\xi_{n}\delta_{0}\left(p\right)+\xi_{n}\left\{\hat{w}\left(p\right)-w_{0}\left(p\right)\right\}\right]=N_{p}=\frac{1}{\sqrt{n}}\sum_{i=1}^{n}\lambda_{i}\psi \left(r_{i,[p]}\right)X_{i}\left(p\right).$$

Therefore, we have

$$\begin{array}{cc} c_{\tau ,p}\sqrt{n}\left(\tau +\xi_{n}\right)\left\{b_{p}-\delta_{0}\left(p\right)+\hat{w}\left(p\right)-w_{0}\left(p\right)\right\}= & \frac{1}{\sqrt{n}}\sum_{i=1}^{n}c_{\tau ,p}\lambda_{i}\psi \left(r_{i,[p]}\right)X_{i}\left(p\right)\\ \; & -c_{\tau ,p}\sqrt{n}\tau \left\{\delta_{0}\left(p\right)-\hat{w}\left(p\right)+w_{0}\left(p\right)\right\}. \end{array}$$

Note that $c_{\tau ,p}\lambda_{i}\psi \left(r_{i,[p]}\right)$ is independent of $X_{i}\left(p\right)$, and $X_{i}\left(p\right)$’s are independent with mean zero and variance one. We have

(A23) $$\begin{array}{cc} E\left[c_{\tau ,p}^{2}n{\left(\tau +\xi_{n}\right)}^{2}{\left\{b_{p}-\delta_{0}\left(p\right)+\hat{w}\left(p\right)-w_{0}\left(p\right)\right\}}^{2}\right]= & \frac{1}{n}\sum_{i=1}^{n}E\left[{\left\{c_{\tau ,p}\lambda_{i}\psi \left(r_{i,[p]}\right)\right\}}^{2}\right]\\ \; & +n\tau^{2}{\left\{\delta_{0}\left(p\right)-\hat{w}\left(p\right)+w_{0}\left(p\right)\right\}}^{2}E\left(c_{\tau ,p}^{2}\right). \end{array}$$

Recall that Proposition A6 gives that

$$|b_{p}-\delta_{0}\left(p\right)+\hat{w}\left(p\right)-w_{0}\left(p\right)|\leq \frac{1}{\sqrt{n}\tau }|N_{p}|+∥\delta_{0}{∥}_{\infty }+\left|\hat{w}\left(p\right)-w_{0}\left(p\right)\right|.$$

Under Assumption **P3**, $n∥\delta_{0}{∥}_{\infty }^{2}\mathrm{polyLog}\left(n\right)n^{2\alpha -1/2}\to 0$. Together with Equations (A21) and (A16), the LHS of (A23) can be written as

$$\begin{array}{cc} \; & E\left[c_{\tau ,p}^{2}{\left(\tau +\xi_{n}\right)}^{2}n{\left\{b_{p}-\delta_{0}\left(p\right)+\hat{w}\left(p\right)-w_{0}\left(p\right)\right\}}^{2}\right]\\ = & E\left[{\left\{c_{\tau ,p}\left(\tau +\xi_{n}\right)-\frac{p-1}{n}+\frac{p-1}{n}\right\}}^{2}n{\left\{b_{p}-\delta_{0}\left(p\right)+\hat{w}\left(p\right)-w_{0}\left(p\right)\right\}}^{2}\right]\\ = & {\left(\frac{p}{n}\right)}^{2}nE\left[{\left\{b_{p}-\delta_{0}\left(p\right)+\hat{w}\left(p\right)-w_{0}\left(p\right)\right\}}^{2}\right]+o\left(1\right). \end{array}$$

This implies Equation (A22). □

On $d_{i,p}$

Recall that

$$d_{i,p}=\psi^{\prime }\left(\gamma_{i,p}^{*}\right)-\psi^{\prime }\left(r_{i,[p]}\right),$$

where $\gamma_{i,p}^{*}$ in the interval $\left(r_{i,[p]},r_{i,[p]}+\nu_{i}\right)$, with

$$\begin{array}{cc} \nu_{i} & =\left\{b_{p}-\delta_{0}\left(p\right)+\hat{w}\left(p\right)-w_{0}\left(p\right)\right\}x_{i}^{⊤}\left[\begin{array}{c} {\left(S_{p}+\tau I\right)}^{-1}u_{p}\\ -1 \end{array}\right]\\ \; & =\left\{b_{p}-\delta_{0}\left(p\right)+\hat{w}\left(p\right)-w_{0}\left(p\right)\right\}\pi_{i}. \end{array}$$

We have the following result.

**Proposition A8.** 
*Under Assumptions* ***O1–O7*** *and* ***P1–P3*** *, we have, at fixed τ,*

$$\underset{i}{\sup}\left|d_{i,p}\right|=O_{L_{k}}\left(\frac{\mathrm{polyLog}\left(n\right)n^{\alpha }}{n^{1/2}\wedge n^{e}}\right).$$

**Proof.** Note that we can write

$$\pi_{i}=x_{i}^{⊤}\left[\begin{array}{c} {\left(S_{p}+\tau I\right)}^{-1}u_{p}\\ -1 \end{array}\right]=v_{i}^{⊤}{\left(S_{p}+\tau I\right)}^{-1}u_{p}-x_{i}\left(p\right).$$

Recall that $u_{p}=n^{-1}V^{⊤}D_{\psi^{\prime }\left(r_{\cdot ,[p]}\right)}X\left(p\right)$. We can also write it as

$$u_{p}=\frac{1}{n}V^{⊤}D_{\lambda_{i}^{2}\psi^{\prime }\left(r_{\cdot ,[p]}\right)}X\left(p\right).$$

Using the independence of X(p) with ${\left\{\left(V_{i},\epsilon_{i}\right)\right\}}_{i=1}^{n}$, and the concentration assumptions on X(p) in Assumption **P1**, according to Lemma 3.36 in [21], we have

$$\underset{i}{\sup}\left|v_{i}^{⊤}{\left(S_{p}+\tau I\right)}^{-1}u_{p}\right|=O_{L_{k}}\left(\frac{\mathrm{polyLog}(n)}{c_{n}^{1/2}}\underset{i}{\sup}∥\frac{1}{n}D_{\lambda_{i}^{2}\psi^{\prime }\left(r_{\cdot ,[p]}\right)}V{\left(S_{p}+\tau I\right)}^{-1}V_{i}∥\right),$$

where we view $v_{i}^{⊤}{\left(S_{p}+\tau I\right)}^{-1}u_{p}$ as a linear form in $V_{i}$. Note that we have absorbed the $\sup_{i}\left|\lambda_{i}\right|$ in the polyLog(n) term. We can write

$$∥\frac{1}{n}D_{\lambda_{i}^{2}\psi^{\prime }\left(r_{\cdot ,[p]}\right)}V{\left(S_{p}+\tau I\right)}^{-1}V_{i}∥=\frac{1}{n}V_{i}^{⊤}{\left(S_{p}+\tau I\right)}^{-1}\frac{V^{⊤}D_{\lambda_{i}^{2}\psi^{\prime }\left(r_{\cdot ,[p]}\right)}^{2}V}{n}{\left(S_{p}+\tau I\right)}^{-1}V_{i}.$$

We notice that $S_{p}=n^{-1}V^{⊤}D_{\lambda_{i}^{2}\psi^{\prime }\left(r_{\cdot ,[p]}\right)}V$. Hence,

$$\frac{V^{⊤}D_{\lambda_{i}^{2}\psi^{\prime }\left(r_{\cdot ,[p]}\right)}^{2}V}{n}⪯{∥D_{\lambda_{i}^{2}\psi^{\prime }\left(r_{\cdot ,[p]}\right)}∥}_{2}S_{p}.$$

Therefore, we conclude that

$$\frac{1}{n}V_{i}^{⊤}{\left(S_{p}+\tau I\right)}^{-1}\frac{V^{⊤}D_{\lambda_{i}^{2}\psi^{\prime }\left(r_{\cdot ,[p]}\right)}^{2}V}{n}{\left(S_{p}+\tau I\right)}^{-1}V_{i}\leq \frac{∥V_{i}{∥}^{2}}{n\tau }{∥D_{\lambda_{i}^{2}\psi^{\prime }\left(r_{\cdot ,[p]}\right)}∥}_{2}=\frac{∥V_{i}{∥}^{2}}{n\tau }\underset{i}{\sup}\lambda_{i}^{2}\psi^{\prime }\left(r_{i,[p]}\right).$$

Note that $\sup_{i}\left|x_{i}\left(p\right)\right|=O_{L_{k}}\left(\mathrm{polyLog}\left(n\right)/\sqrt{c_{n}}\right)$ under Assumption **O4**, **O6** and **P1**, according to Appendix 7 in [21]. Therefore, we have

$$\begin{array}{cc} \underset{i}{\sup}\left|\pi_{i}\right| & =O_{L_{k}}\left[\frac{\mathrm{polyLog}(n)}{c_{n}^{1/2}}\left\{1+\sqrt{\sup_{i}\lambda_{i}^{2}\psi^{\prime }\left(r_{i,[p]}\right)\sup_{i}\frac{∥V_{i}{∥}^{2}}{n\tau }}\right\}\right]\\ \; & =O_{L_{k}}\left[\frac{\mathrm{polyLog}(n)}{c_{n}^{1/2}}\left\{1+\sqrt{\sup_{i}\lambda_{i}^{2}\psi^{\prime }\left(r_{i,[p]}\right)}\right\}\right]\\ \; & =O_{L_{k}}\left\{\mathrm{polyLog}\left(n\right)\right\}. \end{array}$$

Recall that $|b_{p}-\delta_{0}\left(p\right)+\hat{w}\left(p\right)-w_{0}\left(p\right)|=O_{L_{k}}(\mathrm{polyLog}\left(n\right)n^{-1/2}+∥\delta_{0}{∥}_{\infty }+|\hat{w}\left(p\right)-w_{0}\left(p\right)\left|\right)$ and by Lemma A1 we have

$$|\hat{w}\left(p\right)-w_{0}\left(p\right)|=O_{L_{k}}\left(\frac{\mathrm{polyLog}(n)}{\sqrt{n}\wedge n^{e}}\right).$$

We have

$$\underset{i}{\sup}\left|\nu_{i}\right|=O_{L_{k}}\left(\frac{\mathrm{polyLog}(n)}{\sqrt{n}\wedge n^{e}}\right).$$

Note that [21] has shown that Under our assumption that $\psi^{\prime }$ is $Cn^{\alpha }$-Lipschitz, we see that

$$\underset{i}{\sup}\left|d_{i,p}\right|=O_{L_{k}}\left(\frac{\mathrm{polyLog}\left(n\right)n^{\alpha }}{n^{1/2}\wedge n^{e}}\right).$$

□

#### Appendix E.4.3. Final Conclusions

Gathering all the results, we have the following Theorem.

**Theorem A2.** 
*Under Assumptions* ***O1–O7*** *and* ***P1–P3*** *, we have, for any fixed τ,*

$$∥\hat{\delta }-\tilde{b}∥\leq O_{L_{k}}\left(\frac{\mathrm{polyLog}\left(n\right)n^{\alpha }}{{\left(n^{1/2}\wedge n^{e}\right)}^{2}}\right).$$

*In particular,*

$$\begin{array}{cc} \sqrt{n}\left({\hat{\delta }}_{p}-b_{p}\right) & =O_{L_{k}}\left(\frac{\mathrm{polyLog}\left(n\right)n^{\alpha +1/2}}{{\left(n^{1/2}\wedge n^{e}\right)}^{2}}\right),\\ \underset{i}{\sup}\left|x_{i}^{⊤}\left(\hat{\delta }-\tilde{b}\right)\right| & =O_{L_{k}}\left(\frac{\mathrm{polyLog}\left(n\right)n^{\alpha +1/2}}{{\left(n^{1/2}\wedge n^{e}\right)}^{2}}\right),\\ \underset{i}{\sup}\left|R_{i}-r_{i,[p]}\right| & =O_{L_{k}}\left(\left\{\frac{\mathrm{polyLog}(n)}{\sqrt{n}\wedge n^{e}}\right\}\vee \left\{\frac{\mathrm{polyLog}\left(n\right)n^{\alpha +1/2}}{{\left(n^{1/2}\wedge n^{e}\right)}^{2}}\right\}\right). \end{array}$$

**Proof.** The first two results are direct consequences of all our results, using the key bound on $∥\hat{\delta }-\tilde{b}∥$ of Proposition A6. The third result is a direct consequence of the fact that $\sup_{i}∥X_{i}∥/n^{1/2}=O_{L_{k}}\left(1\right)$, which was shown in the proof of Lemma A6. The last result follows from the fact that $R_{i}-r_{i,[p]}=x_{i}^{⊤}\left(\hat{\delta }-\tilde{b}\right)-\nu_{i}$. The result on $\nu_{i}$ is given in the proof of Proposition A8. □

Recall Equation (A22)

$$\begin{array}{cc} {\left(\frac{p}{n}\right)}^{2}nE\left[{\left\{b_{p}-\delta_{0}\left(p\right)+\hat{w}\left(p\right)-w_{0}\left(p\right)\right\}}^{2}\right]= & \frac{1}{n}\sum_{i=1}^{n}E\left[{\left\{c_{\tau ,p}\lambda_{i}\psi \left(r_{i,[p]}\right)\right\}}^{2}\right]\\ \; & +n\tau^{2}{\left\{\delta_{0}\left(p\right)-\hat{w}\left(p\right)+w_{0}\left(p\right)\right\}}^{2}E\left(c_{\tau ,p}^{2}\right)+o\left(1\right). \end{array}$$

and Equation (A17)

$$|b_{p}-\delta_{0}\left(p\right)+\hat{w}\left(p\right)-w_{0}\left(p\right)|=O_{L_{k}}\left(\frac{\mathrm{polyLog}(n)}{\sqrt{n}\wedge n^{e}}\right).$$

We have

$$\begin{array}{cc} \; & {\left(\frac{p}{n}\right)}^{2}nE\left[{\left\{{\hat{\delta }}_{p}-\delta_{0}\left(p\right)+\hat{w}\left(p\right)-w_{0}\left(p\right)\right\}}^{2}\right]\\ \; & \;\;={\left(\frac{p}{n}\right)}^{2}nE\left[{\left\{{\hat{\delta }}_{p}-b_{p}+b_{p}-\delta_{0}\left(p\right)+\hat{w}\left(p\right)-w_{0}\left(p\right)\right\}}^{2}\right]\\ \; & \;\;=\frac{1}{n}\sum_{i=1}^{n}E\left[{\left\{c_{\tau ,p}\lambda_{i}\psi \left(r_{i,[p]}\right)\right\}}^{2}\right]+n\tau^{2}{\left\{\delta_{0}\left(p\right)-\hat{w}\left(p\right)+w_{0}\left(p\right)\right\}}^{2}E\left(c_{\tau ,p}^{2}\right)+o\left(A\right), \end{array}$$

where $A=\frac{1}{n}\sum_{i=1}^{n}E\left[{\left\{c_{\tau ,p}\lambda_{i}\psi \left(r_{i,[p]}\right)\right\}}^{2}\right]+n\tau^{2}{\left\{\delta_{0}\left(p\right)-\hat{w}\left(p\right)+w_{0}\left(p\right)\right\}}^{2}E\left(c_{\tau ,p}^{2}\right)$.

We note that the index *p* in Equation (A22) and the previous theorem plays no particular role, and similar results hold when *p* is replaced by any *k* in the range 1≤k≤p. Summing over all the coordinates, we have under Assumptions **O1–O7** and **P1–P4**,

$$\begin{array}{cc} {\left(\frac{p}{n}\right)}^{2}E(∥\hat{\delta }-\delta_{0}+\hat{w}-w_{0}{∥}^{2})= & \frac{1}{n}\sum_{k=1}^{p}\left(\frac{1}{n}\sum_{i=1}^{n}E\left[{\left\{c_{\tau ,k}\lambda_{i}\psi \left(r_{i,[k]}\right)\right\}}^{2}\right]\right)\\ \; & +\tau^{2}\sum_{k=1}^{p}{\left\{\delta_{0}\left(k\right)-\hat{w}\left(k\right)+w_{0}\left(k\right)\right\}}^{2}E\left(c_{\tau ,k}^{2}\right)+o\left(B\right), \end{array}$$

where $B=\frac{1}{n}\sum_{k=1}^{p}\left(\frac{1}{n}\sum_{i=1}^{n}E\left[{\left\{c_{\tau ,k}\lambda_{i}\psi \left(r_{i,[k]}\right)\right\}}^{2}\right]\right)+\tau^{2}\sum_{k=1}^{p}{\left\{\delta_{0}\left(k\right)-\hat{w}\left(k\right)+w_{0}\left(k\right)\right\}}^{2}E\left(c_{\tau ,k}^{2}\right)$.

Note that $0\leq c_{\tau ,k}\leq \kappa /\tau$, $n^{-1}\sum_{i=1}^{n}{∥\psi^{2}∥}_{\infty }\leq C$ from Assumption **O3**, and $∥\delta_{0}{∥}^{2}=O\left(1\right)$. We have B=O(1). Therefore, we have

$$\begin{array}{cc} {\left(\frac{p}{n}\right)}^{2}E(∥\hat{\delta }-\delta_{0}+\hat{w}-w_{0}{∥}^{2})= & \frac{1}{n}\sum_{k=1}^{p}\left(\frac{1}{n}\sum_{i=1}^{n}E\left[{\left\{c_{\tau ,k}\lambda_{i}\psi \left(r_{i,[k]}\right)\right\}}^{2}\right]\right)\\ \; & +\tau^{2}\sum_{k=1}^{p}{\left\{\delta_{0}\left(k\right)-\hat{w}\left(k\right)+w_{0}\left(k\right)\right\}}^{2}E\left(c_{\tau ,k}^{2}\right)+o\left(1\right). \end{array}$$

On $c_{\tau ,k}$ and $c_{\tau }$

We now show that $c_{\tau ,k}$ and $c_{\tau }$ are close to each other.

**Proposition A9.** 
*We have, under Assumptions* ***O1–O7*** *and* ***P1–P3*** *,*

$$\underset{1\leq k\leq p}{\sup}\left|c_{\tau ,k}-c_{\tau }\right|=O_{L_{k}}\left(\left[\frac{\mathrm{polyLog}\left(n\right)n^{\alpha }}{\sqrt{n}\wedge n^{e}}\right]\vee \left[\frac{\mathrm{polyLog}\left(n\right)n^{2\alpha +1/2}}{{\left(n^{1/2}\wedge n^{e}\right)}^{2}}\right]\vee \frac{\mathrm{polyLog}(n)}{n}\right).$$

*Of course, we also have $0\leq c_{\tau }\leq p/\left(n\tau \right)$ and $0\leq c_{\tau ,k}\leq p/\left(n\tau \right)$.*

**Proof.** Recall that

$$S=\frac{1}{n}\sum_{i=1}^{n}\psi^{\prime }\left(R_{i}\right)x_{i}x_{i}^{⊤}.$$

Denote $\Gamma =n^{-1}\sum_{i=1}^{n}\psi^{\prime }\left(R_{i}\right)vv^{⊤}$ and $a=n^{-1}\sum_{i=1}^{n}\psi^{\prime }\left(R_{i}\right)x_{i}^{2}\left(p\right)$, we have

$$S=\left(\begin{array}{cc} \Gamma & v\\ v & a \end{array}\right).$$

According to Lemma 3.40 in [21], we have, since $c_{\tau }=n^{-1}\mathrm{tr}\left\{{\left(S+\tau I_{p}\right)}^{-1}\right\}$,

$$|c_{\tau }-\frac{1}{n}\mathrm{tr}\left\{{\left(\Gamma +\tau I_{p-1}\right)}^{-1}\right\}|\leq \frac{1}{n}\frac{1+a/\tau }{\tau }.$$

We also have

$$a=\frac{1}{n}\sum_{i=1}^{n}\lambda_{i}^{2}X_{i}^{2}\left(p\right)\psi^{\prime }\left(R_{i}\right)\leq \mathrm{polyLog}\left(n\right)\frac{1}{n}\sum_{i=1}^{n}\lambda_{i}^{2}X_{i}^{2}\left(p\right)=O_{L_{k}}\left(\mathrm{polyLog}\left(n\right)\right).$$

Since $\psi^{\prime }$ is $Cn^{\alpha }$-Lipschitz and

$$\underset{i}{\sup}\left|R_{i}-r_{i,[p]}\right|=O_{L_{k}}\left(\left\{\frac{\mathrm{polyLog}(n)}{\sqrt{n}\wedge n^{e}}\right\}\vee \left\{\frac{\mathrm{polyLog}\left(n\right)n^{\alpha +1/2}}{{\left(n^{1/2}\wedge n^{e}\right)}^{2}}\right\}\right),$$

we have

$$\underset{i}{\sup}\left|\psi^{\prime }\left(R_{i}\right)-\psi^{\prime }\left(r_{i,[p]}\right)\right|=O_{L_{k}}\left(\left\{\frac{\mathrm{polyLog}\left(n\right)n^{\alpha }}{\sqrt{n}\wedge n^{e}}\right\}\vee \left\{\frac{\mathrm{polyLog}\left(n\right)n^{2\alpha +1/2}}{{\left(n^{1/2}\wedge n^{e}\right)}^{2}}\right\}\right),$$

Hence, using arguments similar to those in the proof of Lemma A9, we have

$$|\frac{1}{n}\mathrm{tr}\left\{{\left(\Gamma +\tau I_{p-1}\right)}^{-1}\right\}-\frac{1}{n}\mathrm{tr}\left\{{\left(S_{p}+\tau I_{p}\right)}^{-1}\right\}|=O_{L_{k}}\left(\left[\frac{\mathrm{polyLog}\left(n\right)n^{\alpha }}{\sqrt{n}\wedge n^{e}}\right]\vee \left[\frac{\mathrm{polyLog}\left(n\right)n^{2\alpha +1/2}}{{\left(n^{1/2}\wedge n^{e}\right)}^{2}}\right]\right).$$

Since $c_{\tau ,k}=n^{-1}\mathrm{tr}\left\{{\left(S_{p}+\tau I_{p}\right)}^{-1}\right\}$, the result follows immediately. We note that *p* did not play a particular role in the proof and hence taking the sup over those indices only adds a polyLog(n) term. Hence the result holds for $\sup_{1\leq k\leq n}\left|c_{\tau ,k}-c_{\tau }\right|$. □

We are now ready to prove the last proposition of this section, which will help us obtain the second equation of our System.

**Proposition A10.** 

(A24)
$$\begin{array}{cc} {\left(\frac{p}{n}\right)}^{2}E(∥\hat{\delta }-\delta_{0}+\hat{w}-w_{0}{∥}^{2})= & \frac{p}{n}\frac{1}{n}\sum_{i=1}^{n}E\left[{\left\{c_{\tau }\lambda_{i}\psi \left(\mathrm{prox}\left(c_{\tau }\lambda_{i}^{2}\rho \right)\left({\tilde{r}}_{i,(i)}\right)\right)\right\}}^{2}\right]\\ \; & +\tau^{2}{∥\delta_{0}+w_{0}-\hat{w}∥}^{2}E\left(c_{\tau }^{2}\right)+o\left(1\right). \end{array}$$

*Furthermore, when all $\lambda_{i}$’s are non-zero,*

$$\frac{1}{n}\sum_{i=1}^{n}E\left[{\left\{c_{\tau }\lambda_{i}\psi \left(\mathrm{prox}\left(c_{\tau }\lambda_{i}^{2}\rho \right)\left({\tilde{r}}_{i,(i)}\right)\right)\right\}}^{2}\right]=\frac{1}{n}\sum_{i=1}^{n}E\left(\frac{{\left\{{\tilde{r}}_{i,(i)}-\mathrm{prox}\left(c_{\tau }\lambda_{i}^{2}\rho \right)\left({\tilde{r}}_{i,(i)}\right)\right\}}^{2}}{\lambda_{i}^{2}}\right).$$

**Proof.** In light of Proposition A9 and Assumption **P3** which guarantees that $∥\delta_{0}{∥}^{2}$ is uniformly bounded in *p* and *n*, we have

$$\sum_{k=1}^{p}{\left\{\delta_{0}\left(k\right)-\hat{w}\left(k\right)+w_{0}\left(k\right)\right\}}^{2}E\left(c_{\tau ,k}^{2}\right)={∥\delta_{0}+w_{0}-\hat{w}∥}^{2}E\left(c_{\tau }^{2}\right)+o\left(1\right).$$

Using Theorem A2 and the bound on $∥\psi^{\prime }{∥}_{\infty }$ in Assumption **O3**, we have

$$\frac{1}{p}\sum_{k=1}^{p}E\left[{\left\{c_{\tau ,k}\lambda_{i}\psi \left(r_{i,[k]}\right)\right\}}^{2}\right]=\frac{1}{p}\sum_{k=1}^{p}E\left[{\left\{c_{\tau ,k}\lambda_{i}\psi \left(R_{i}\right)\right\}}^{2}\right]+o\left(1\right).$$

With the help of Proposition A9, we have

$$\frac{1}{p}\sum_{k=1}^{p}E\left[{\left\{c_{\tau ,k}\lambda_{i}\psi \left(R_{i}\right)\right\}}^{2}\right]=\frac{1}{p}\sum_{k=1}^{p}E\left[{\left\{c_{\tau }\lambda_{i}\psi \left(R_{i}\right)\right\}}^{2}\right]+o\left(1\right)=E\left[{\left\{c_{\tau }\lambda_{i}\psi \left(R_{i}\right)\right\}}^{2}\right]+o\left(1\right).$$

In light of Equation (A7), we have

$$\frac{1}{n}\sum_{i=1}^{n}E\left\{{\left(c_{\tau }\lambda_{i}\psi \left(R_{i}\right)\right)}^{2}\right\}=\frac{1}{n}\sum_{i=1}^{n}E\left({\left[c_{\tau }\lambda_{i}\psi \left\{\mathrm{prox}\left(c_{i}\rho \right)\left({\tilde{r}}_{i,(i)}\right)\right\}\right]}^{2}\right)+o\left(1\right).$$

Lemma A11 gives the computation of the derivative of prox(cρ)(x) with respect to *c*, which allows us to bound the error $|\psi \left\{\mathrm{prox}\left(c_{i}\rho \right)\left(x\right)\right\}-\psi \left\{\mathrm{prox}\left(c_{\tau }\lambda_{i}^{2}\rho \right)\left(x\right)\right\}|$. In light of this, by using the fact that $\sup_{i}\left|c_{i}-\lambda_{i}^{2}c_{\tau }\right|=O_{L_{k}}\left(n^{2\alpha -1/2}\mathrm{polyLog}\left(n\right)\right)$ in Corollary A1, we can re-express the previous equation as

$$\frac{1}{n}\sum_{i=1}^{n}E\left\{{\left(c_{\tau }\lambda_{i}\psi \left(R_{i}\right)\right)}^{2}\right\}=\frac{1}{n}\sum_{i=1}^{n}E\left({\left[c_{\tau }\lambda_{i}\psi \left\{\mathrm{prox}\left(c_{\tau }\lambda_{i}^{2}\rho \right)\left({\tilde{r}}_{i,(i)}\right)\right\}\right]}^{2}\right)+o\left(1\right).$$

When all $\lambda_{i}$’s are non-zero, we have

$$\frac{1}{n}\sum_{i=1}^{n}E\left\{{\left(c_{\tau }\lambda_{i}\psi \left(R_{i}\right)\right)}^{2}\right\}=\frac{1}{n}\sum_{i=1}^{n}E\left(\frac{{\left[c_{\tau }\lambda_{i}^{2}\psi \left\{\mathrm{prox}\left(c_{\tau }\lambda_{i}^{2}\rho \right)\left({\tilde{r}}_{i,(i)}\right)\right\}\right]}^{2}}{\lambda_{i}^{2}}\right)+o\left(1\right).$$

Finally, since by definition,

∀x∈R,x=prox(cρ)(x)+cψ(prox(cρ)(x)),

we have

$$\frac{1}{n}\sum_{i=1}^{n}E\left(\frac{{\left[c_{\tau }\lambda_{i}^{2}\psi \left\{\mathrm{prox}\left(c_{\tau }\lambda_{i}^{2}\rho \right)\left({\tilde{r}}_{i,(i)}\right)\right\}\right]}^{2}}{\lambda_{i}^{2}}\right)=\frac{1}{n}\sum_{i=1}^{n}E\left(\frac{{\left[{\tilde{r}}_{i,(i)}-\mathrm{prox}\left(c_{\tau }\lambda_{i}^{2}\rho \right)\left({\tilde{r}}_{i,(i)}\right)\right]}^{2}}{\lambda_{i}^{2}}\right).$$

□

**Lemma A11** 
(El Karoui [21], Lemma 3.32). *Suppose x is a real and ρ is twice differentiable and convex. Then, for c>0, we have*

$$\frac{\partial }{\partial c}\mathrm{prox}\left(c\rho \right)\left(x\right)=-\frac{\psi (\mathrm{prox}(c\rho )(x))}{1+c\psi^{\prime }\left(\mathrm{prox}\left(c\rho \right)\left(x\right)\right)},$$

*and*

$$\frac{\partial }{\partial c}\rho \left(\mathrm{prox}\left(c\rho \right)\left(x\right)\right)=-\frac{\psi^{2}\left(\mathrm{prox}\left(c\rho \right)\left(x\right)\right)}{1+c\psi^{\prime }\left(\mathrm{prox}\left(c\rho \right)\left(x\right)\right)}.$$

*In particular, at x given c→ρ(prox(cρ)(x)) is decreasing in c.*

### Appendix E.5. Last Steps of the Proof

#### Appendix E.5.1. On the Asymptotic Behavior of r ˜ i,(i)

We have the following result.

**Lemma A12.** 
*Under Assumptions* ***O1–O7*** *and* ***P1–P4*** *, as n and p tend to infinity, ${\tilde{r}}_{i,(i)}=\epsilon_{i}+x_{i}^{⊤}\left(w_{0}-\hat{w}\right)-x_{i}^{⊤}\left({\hat{\delta }}_{\left(i\right)}-\delta_{0}\right)$ behaves like $\epsilon_{i}+\lambda_{i}\sqrt{E(∥\hat{\beta }-\beta_{0}{∥}^{2})}Z_{i}$. where $Z_{i}∼N\left(0,1\right)$ is independent of $\epsilon_{i}$ and $\lambda_{i}$, in the sense of weak convergence.*
*Furthermore, if i≠j, ${\tilde{r}}_{i,(i)}$ and ${\tilde{r}}_{j,(j)}$ are asymptotically (pairwise) independent. The same is true for $\left({\tilde{r}}_{i,(i)},\lambda_{i}\right)$ and $\left({\tilde{r}}_{j,(j)},\lambda_{j}\right)$.*

**Proof.** 
**First part.**
In this part, we will show that $X_{i}^{⊤}\left({\hat{\delta }}_{\left(i\right)}-\delta_{0}+\hat{w}-w_{0}\right)$ is asymptotically $N(0,E(∥\hat{\beta }-\beta_{0}{∥}^{2}))$. Recall that ${\hat{\delta }}_{\left(i\right)}$ is independent of $X_{i}$ and that $E(X_{i})=0$, $\mathrm{cov}(X_{i})=I$ and that, for any finite *k*, the first *k* moments of its entries are bounded uniformly in *n*. We have shown that in Proposition A4 that $\mathrm{var}(∥\hat{\beta }-\beta_{0}{∥}^{2})\to 0$. In light of Lemma A3, we also know that $E(∥\hat{\beta }-\beta_{0}{∥}^{2})$ is uniformly bounded. Furthermore, in the proof of Proposition A4, we showed that $E(∥\hat{\delta }+\hat{w}-\delta_{0}-w_{0}{∥}^{2}-∥{\hat{\delta }}_{\left(i\right)}+\hat{w}-\delta_{0}-w_{0}{∥}^{2})\to 0$. We use a simple generalization of the standard Lindeberg-Feller theorem (see e.g., [38]). Indeed, if $a_{n,p}\left(k\right)$ are random variables with $\sqrt{\sum_{k=1}^{p}a_{n,p}{\left(k\right)}^{2}}=A_{n}$, $E(A_{n}^{2})$ remains bounded in *n*, and $a_{n,p}\left(k\right)$’s are independent of $X_{i}$, we see that: a) if $z∼N(0,I_{p})$, independent of $a_{n,p}\left(k\right)$, then $a_{n,p}^{⊤}z∼A_{n}N$ where N∼N(0,1) and independent of $A_{n}$ (conditionally and unconditionally on $a_{n,p}$); b) Theorem 2.1.5 and its proof in [38] hold provided that $\sum_{i=1}^{n}E(|a_{n,p}\left(k\right){|}^{3})=o\left(1\right)$. The proof simply needs to be started conditionally on $a_{n,p}\left(k\right)$, and the final moment bounds are then taken unconditionally. This very mild generalization gives, if ϕ is a $C^{3}$ function, with bounded 2nd and 3rd derivatives,

$$\begin{array}{cc} \; & \forall \epsilon >0,|E\left\{\phi \left(a_{n,p}^{⊤}X_{i}\right)\right\}-E\left\{\phi \left(A_{n}N\right)\right\}|\\ \; & \;\leq K\left[\epsilon ∥\phi^{\left(3\right)}{∥}_{\infty }E\left\{\sum_{k=1}^{p}a_{n,p}{\left(k\right)}^{2}\right\}+\frac{∥\phi^{\left(2\right)}{∥}_{\infty }}{\epsilon }\sum_{k=1}^{p}E(|a_{n,p}\left(k\right){|}^{3})\right], \end{array}$$

where *K* is a constant depending on the second and third absolute moments of the entries of $X_{i}$. It is therefore independent of *n* and *p* under our assumptions on $X_{i}$. We can apply this result to $a_{n,p}\left(k\right)={\hat{\delta }}_{\left(i\right)}\left(k\right)-\delta_{0}\left(k\right)+\hat{w}\left(k\right)-w_{0}\left(k\right)$. Recall that we have shown that

$$\hat{\delta }\left(k\right)-\delta_{0}\left(k\right)+\hat{w}\left(k\right)-w_{0}\left(k\right)=O_{L_{k}}\left(\frac{\mathrm{polyLog}(n)}{n^{1/2}\wedge n^{e}}\right)$$

for each coordinate *k*. The same arguments apply also to ${\hat{\delta }}_{\left(i\right)}\left(k\right)$, the *k*th coordinate of ${\hat{\delta }}_{\left(i\right)}$. We have

$$E(|{\hat{\delta }}_{\left(i\right)}\left(k\right)-\delta_{0}\left(k\right)+\hat{w}\left(k\right)-w_{0}\left(k\right){|}^{3})=O\left\{\frac{\mathrm{polyLog}(n)}{{\left(n^{1/2}\wedge n^{e}\right)}^{3}}\right\}$$

Provided that e>1/3, we have

$$E\left(\sum_{k=1}^{p}{\left|{\hat{\delta }}_{\left(i\right)}\left(k\right)-\delta_{0}\left(k\right)+\hat{w}\left(k\right)-w_{0}\left(k\right)\right|}^{3}\right)=O\left\{\frac{\mathrm{polyLog}(n)n}{{\left(n^{1/2}\wedge n^{e}\right)}^{3}}\right\}=o\left(1\right).$$

Therefore, in connection with Corollary 2.1.9 in [38], $X_{i}^{⊤}\left({\hat{\delta }}_{\left(i\right)}-\delta_{0}+\hat{w}-w_{0}\right)$ behaves asymptotically like $∥{\hat{\delta }}_{\left(i\right)}-\delta_{0}+\hat{w}-w_{0}∥N$ in the sense of weak convergence. Since $∥{\hat{\delta }}_{\left(i\right)}-\delta_{0}+\hat{w}-w_{0}∥-E∥{\hat{\delta }}_{\left(i\right)}-\delta_{0}+\hat{w}-w_{0}∥\to 0$ in probability and the expectations are uniformly bounded, Slutsky’s lemma gives that

$$X_{i}^{⊤}\left({\hat{\delta }}_{\left(i\right)}-\delta_{0}+\hat{w}-w_{0}\right)\;\mathrm{behaves}\;\mathrm{like}\;E(∥{\hat{\delta }}_{\left(i\right)}-\delta_{0}+\hat{w}-w_{0}∥)N$$

asymptotically in the sense of weak convergence. Using the fact that $E(∥\hat{\delta }-\delta_{0}+\hat{w}-w_{0}{∥}^{2}-∥{\hat{\delta }}_{\left(i\right)}-\delta_{0}+\hat{w}-w_{0}{∥}^{2})\to 0$, another application of Slutsky’s lemma yields

$$X_{i}^{⊤}\left({\hat{\delta }}_{\left(i\right)}-\delta_{0}+\hat{w}-w_{0}\right)\;\mathrm{behaves}\;\mathrm{like}\;E(∥\hat{\delta }-\delta_{0}+\hat{w}-w_{0}∥)N$$

in the sense of weak convergence. We note that the same reasoning applies when replacing $a_{n,p}\left(k\right)={\hat{\delta }}_{\left(i\right)}\left(k\right)-\delta_{0}\left(k\right)+\hat{w}\left(k\right)-w_{0}\left(k\right)$ by ${\tilde{a}}_{n,p}\left(k\right)=\lambda_{i}\left\{{\hat{\delta }}_{\left(i\right)}\left(k\right)-\delta_{0}\left(k\right)+\hat{w}\left(k\right)-w_{0}\left(k\right)\right\}$, provided that $\lambda_{i}$ has 3 moments. It shows that

$$X_{i}^{⊤}\left({\hat{\delta }}_{\left(i\right)}-\delta_{0}+\hat{w}-w_{0}\right)\;\mathrm{behaves}\;\mathrm{like}\;\lambda_{i}E(∥\hat{\beta }-\beta_{0}∥)N.$$

This shows the first part of the lemma, since $E(∥\hat{\beta }-\beta_{0}∥)=\sqrt{E(∥\hat{\beta }-\beta_{0}{∥}^{2})}+o\left(1\right)$ by Proposition A4.
**Second part.**
For the second part, we use a leave-two-out approach. More precisely, we use the approximation

$${\tilde{r}}_{i,(i)}=\epsilon_{i}+x_{i}^{⊤}\left(w_{0}-\hat{w}\right)-x_{i}^{⊤}\left({\hat{\delta }}_{\left(i\right)}-\delta_{0}\right)=\epsilon_{i}+x_{i}^{⊤}\left(w_{0}-\hat{w}\right)-x_{i}^{⊤}\left({\hat{\delta }}_{\left(ij\right)}-\delta_{0}\right)+o_{L_{k}}\left(1\right),$$

and similarly for ${\tilde{r}}_{j,(j)}$, which follows from Theorem A1. Here ${\hat{\delta }}_{\left(ij\right)}$ is computed by solving Problem (5) without $\left(x_{i},y_{i}\right)$ and $\left(x_{j},y_{j}\right)$. It is clear that ${\tilde{r}}_{i,(i)}$ and ${\tilde{r}}_{j,(j)}$ are asymptotically independent conditional on $X_{\left(ij\right)}$, i.e., the design matrix without the *i*-th and *j*-th rows. Similarly, we have

$$E\left(\sum_{k=1}^{p}{\left|{\hat{\delta }}_{\left(ij\right)}\left(k\right)-\delta_{0}\left(k\right)+\hat{w}\left(k\right)-w_{0}\left(k\right)\right|}^{3}\right)=O\left\{\frac{\mathrm{polyLog}(n)n}{{\left(n^{1/2}\wedge n^{e}\right)}^{3}}\right\}=o\left(1\right).$$

Therefore, we also have

$$\sum_{k=1}^{p}{\left|{\hat{\delta }}_{\left(ij\right)}\left(k\right)-\delta_{0}\left(k\right)+\hat{w}\left(k\right)-w_{0}\left(k\right)\right|}^{3}=o_{p}\left(1\right).$$

Note that ${\hat{\delta }}_{\left(ij\right)}$ depends only on $\left\{X_{\left(ij\right)},\epsilon_{\left(ij\right)}\right\}$. We call $P_{\left(ij\right)}$ the joint probability measure $P_{\left(ij\right)}=\prod_{k\neq (i,j)}P_{x_{k},\epsilon_{k}}$, i.e., probability computed with respect to all our random variables except $\left(x_{i},\epsilon_{i}\right)$ and $\left(x_{j},\epsilon_{j}\right)$. So we have found $E_{\left(ij\right)}^{n}$, which depends only on $\left(X_{\left(ij\right)},\epsilon_{\left(ij\right)}\right)$, such that $P_{\left(ij\right)}\left(E_{\left(ij\right)}^{n}\right)\to 1$ and $\sum_{k=1}^{p}{\left|{\hat{\delta }}_{\left(ij\right)}\left(k\right)-\delta_{0}\left(k\right)+\hat{w}\left(k\right)-w_{0}\left(k\right)\right|}^{3}=o\left(1\right)$ when $\left(X_{\left(ij\right)},{\left\{\epsilon_{k}\right\}}_{k\neq (i,j)}\right)\in E_{\left(ij\right)}^{n}$. By treating $a_{n,p}$’s as deterministic quantities, the arguments we gave above then imply that, when $\left(X_{\left(ij\right)},\epsilon_{\left(ij\right)}\right)\in E_{\left(ij\right)}^{n}$,

$$X_{i}^{⊤}\left({\hat{\delta }}_{\left(ij\right)}-\delta_{0}+\hat{w}-w_{0}\right)|\left(X_{\left(ij\right)},\epsilon_{\left(ij\right)}\right)\;\mathrm{behaves}\;\mathrm{like}\;(∥{\hat{\delta }}_{\left(ij\right)}-\delta_{0}∥)N.$$

We now use characteristic function arguments. Let $\alpha_{i}=X_{i}^{⊤}\left({\hat{\delta }}_{\left(ij\right)}-\delta_{0}+\hat{w}-w_{0}\right)$ and $\alpha_{j}=X_{j}^{⊤}\left({\hat{\delta }}_{\left(ij\right)}-\delta_{0}+\hat{w}-w_{0}\right)$. Let $\left(w_{i},w_{j}\right)\in R^{2}$ be fixed and

(A25) $$\chi \left(w_{i},w_{j}\right)=E\left\{e^{i(w_{1}\alpha_{i}+w_{2}\alpha_{j})}\right\}=E\left\{e^{i(w_{1}\alpha_{i}+w_{2}\alpha_{j})}\left(1_{E_{\left(ij\right)}^{n}}+1_{{\left[E_{\left(ij\right)}^{n}\right]}^{c}}\right)\right\}.$$

Since $P\left(E_{\left(ij\right)}^{n}\right)=P_{\left(ij\right)}\left(E_{\left(ij\right)}^{n}\right)\to 1$, we can just focus on $E\{e^{i(w_{1}\alpha_{i}+w_{2}\alpha_{j})}1_{E_{\left(ij\right)}^{n}}\}$, since the modulus of the functions we are integrating is bounded by 1. Now, we have

$$E\left\{e^{i(w_{1}\alpha_{i}+w_{2}\alpha_{j})}1_{E_{\left(ij\right)}^{n}}\right\}=E\left[1_{E_{\left(ij\right)}^{n}}E\left\{e^{i(w_{1}\alpha_{i}+w_{2}\alpha_{j})}|X_{\left(ij\right)},\epsilon_{\left(ij\right)}\right\}\right],$$

since $1_{E_{\left(ij\right)}^{n}}$ is a deterministic function of $\left(X_{\left(ij\right)},\epsilon_{\left(ij\right)}\right)$. Independence of $X_{i}$ and $X_{j}$ gives

$$E\left\{e^{i(w_{1}\alpha_{i}+w_{2}\alpha_{j})}|X_{\left(ij\right)},\epsilon_{\left(ij\right)}\right\}=E\left(e^{iw_{1}\alpha_{i}}|X_{\left(ij\right)},\epsilon_{\left(ij\right)}\right)E\left(e^{iw_{2}\alpha_{j}}|X_{\left(ij\right)},\epsilon_{\left(ij\right)}\right).$$

Also, the conditional Gaussian approximation established above implies that

$$1_{E_{\left(ij\right)}^{n}}\left\{E\left(e^{i(w_{1}\alpha_{i}+w_{2}\alpha_{j})}|X_{\left(ij\right)},\epsilon_{\left(ij\right)}\right)-e^{-\left(w_{1}^{2}/2+w_{2}^{2}/2\right){∥{\hat{\delta }}_{\left(ij\right)}-\delta_{0}∥}^{2}}\right\}\to 0$$

in $P_{\left(ij\right)}$-probability. So we conclude that

$$E\left\{1_{E_{\left(ij\right)}^{n}}e^{i(w_{1}\alpha_{i}+w_{2}\alpha_{j})}\right\}-E\left\{1_{E_{\left(ij\right)}^{n}}e^{-\left(w_{1}^{2}/2+w_{2}^{2}/2\right){∥{\hat{\delta }}_{\left(ij\right)}-\delta_{0}∥}^{2}}\right\}\to 0.$$

Since $P(E_{\left(ij\right)}^{n})\to 1$ and $∥{\hat{\delta }}_{\left(ij\right)}-\delta_{0}{∥}^{2}$ is asymptotically deterministic by arguments similar to those in the proof of Proposition A4, we have

$$E\left\{1_{E_{\left(ij\right)}^{n}}e^{-\left(w_{1}^{2}/2+w_{2}^{2}/2\right){∥{\hat{\delta }}_{\left(ij\right)}-\delta_{0}∥}^{2}}\right\}-e^{-\left(w_{1}^{2}/2+w_{2}^{2}/2\right)E(∥{\hat{\delta }}_{\left(ij\right)}-\delta_{0}{∥}^{2})}\to 0.$$

Therefore,

$$E\left\{e^{i(w_{1}\alpha_{i}+w_{2}\alpha_{j})}\right\}-E\left(e^{iw_{1}\alpha_{i}}\right)E\left(e^{iw_{2}\alpha_{j}}\right)\to 0.$$

This shows that $\alpha_{i}$ and $\alpha_{j}$ are asymptotically independent. It easily follows that ${\tilde{r}}_{i,(i)}$ and ${\tilde{r}}_{j,(j)}$ are asymptotically independent. The same reasoning applies to $\left({\tilde{r}}_{i,(i)},\lambda_{i}\right)$ and $\left({\tilde{r}}_{j,(j)},\lambda_{j}\right)$, since ${\hat{\delta }}_{\left(ij\right)}$ is independent of $\lambda_{i}$ and $\lambda_{j}$ under Assumption **O6**. □

#### Appendix E.5.2. On the Asymptotic Behavior of c τ

We now show that $c_{\tau }=n^{-1}\mathrm{tr}\left\{{\left(S+\tau I_{p}\right)}^{-1}\right\}$ is asymptotically deterministic. This require several steps.

**Lemma A13.** 
*We work under Assumptions* ***O1–O7*** *,* ***P1–P4*** *and* ***F2–F4*** *. Consider the random function*

$$g_{n}\left(x\right)=\frac{1}{n}\sum_{i=1}^{n}\frac{1}{1+x\lambda_{i}^{2}\psi^{\prime }\left\{\mathrm{prox}\left(x\lambda_{i}^{2}\rho \right)\left({\tilde{r}}_{i,(i)}\right)\right\}},\;\mathrm{defined}\;\mathrm{for}\;x\geq 0.$$

*Let B>0 be in $R_{+}$. We have, for any $\left(x,y\right)\in R_{+}^{2}$, and 0≤x≤B, 0≤y≤B,*

$$\underset{(x,y):|x-y|\leq \eta ,0\leq x\leq B,0\leq y\leq B}{\sup}|g_{n}\left(x\right)-g_{n}\left(y\right)|\leq \eta \frac{1}{n}\sum_{i=1}^{n}(\lambda_{i}^{2}∥\psi^{\prime }{∥}_{\infty }+BL\left(n\right)\lambda_{i}^{4}{∥\psi ∥}_{\infty }).$$

*In particular, under Assumption* ***P2*** *and* ***F3–F4*** *, we have for C a constant independent of n and p,*

(A26) $${\mathrm{Pr}}^{*}\left(\underset{(x,y):|x-y|\leq \eta ,0\leq x\leq B,0\leq y\leq B}{\sup}\left|g_{n}\left(x\right)-g_{n}\left(y\right)\right|>\delta \right)\leq \frac{\eta }{\delta }C.$$

*Hence, $g_{n}$ is stochastic equicontinuous on [0,B] for any B>0 given.*

We used the notation ${\mathrm{Pr}}^{*}$ above to denote outer probability and avoid a discussion of potential measure theoretic issues associated with taking a supremum over a noncountable collection of random variables (see e.g., van der Vaart [39] (Sect. 18.2)).

**Proof.** Consider the function defined for x≥0,

$$h_{u}^{\left(i\right)}\left(x\right)=\frac{1}{1+x\lambda_{i}^{2}\psi^{\prime }\left\{\mathrm{prox}\left(x\lambda_{i}^{2}\rho \right)\left(u\right)\right\}}=\frac{\partial }{\partial u}\mathrm{prox}\left(x\lambda_{i}^{2}\rho \right)\left(u\right).$$

The last equality comes from Lemma 3.33 from El Karoui [21]. Since $\psi^{\prime }$ is non-negative,

$$\forall u,\;|h_{u}^{\left(i\right)}\left(x\right)-h_{u}^{\left(i\right)}\left(y\right)|\leq |x\lambda_{i}^{2}\psi^{\prime }\left(\mathrm{prox}\left(x\lambda_{i}^{2}\rho \right)\left(u\right)\right)-y\lambda_{i}^{2}\psi^{\prime }\left(\mathrm{prox}\left(y\lambda_{i}^{2}\rho \right)\left(u\right)\right)|\wedge 1.$$

Thus, since x,y≥0, for all u,

$$\begin{array}{cc} |h_{u}^{\left(i\right)}\left(x\right)-h_{u}^{\left(i\right)}\left(y\right)|\leq & \lambda_{i}^{2}\left|x-y\right|\psi^{\prime }\left(\mathrm{prox}\left(x\lambda_{i}^{2}\rho \right)\left(u\right)\right)\\ \; & +\lambda_{i}^{2}y\left|\psi^{\prime }\left(\mathrm{prox}\left(x\lambda_{i}^{2}\rho \right)\left(u\right)\right)-\psi^{\prime }\left(\mathrm{prox}\left(y\lambda_{i}^{2}\rho \right)\left(u\right)\right)\right|. \end{array}$$

In particular, if |x−y|≤η, and x∨y≤B, with x,y≥0, for all *u*,

$$\begin{array}{cc} \underset{y:|x-y|\leq \eta ;x\vee y\leq B}{\sup}|h_{u}^{\left(i\right)}\left(x\right)-h_{u}^{\left(i\right)}\left(y\right)|\leq & \lambda_{i}^{2}\eta \psi^{\prime }\left\{\mathrm{prox}\left(x\lambda_{i}^{2}\rho \right)\left(u\right)\right\}\\ \; & +B\lambda_{i}^{2}\underset{y:|x-y|\leq \eta ,x\vee y\leq B}{\sup}\left|\psi^{\prime }\left\{\mathrm{prox}\left(x\lambda_{i}^{2}\rho \right)\left(u\right)\right\}-\psi^{\prime }\left\{\mathrm{prox}\left(y\lambda_{i}^{2}\rho \right)\left(u\right)\right\}\right|. \end{array}$$

Under Assumption **O3**, $\psi^{\prime }$ is L(n)-Lipschitz. Therefore, for $x_{i}=x\lambda_{i}^{2}$, $y_{i}=y\lambda_{i}^{2}\geq 0$, we have

$$\forall u,|\psi^{\prime }\left\{\mathrm{prox}\left(x_{i}\rho \right)\left(u\right)\right\}-\psi^{\prime }\left\{\mathrm{prox}\left(y_{i}\rho \right)\left(u\right)\right\}|\leq L\left(n\right)\left|\mathrm{prox}\left(x_{i}\rho \right)\left(u\right)-\mathrm{prox}\left(y_{i}\rho \right)\left(u\right)\right|.$$

Recall that according to Lemma A11,

$$\frac{\partial }{\partial x}\mathrm{prox}\left(x\rho \right)\left(u\right)=-\frac{\psi \{\mathrm{prox}(x\rho )(u)\}}{1+x\psi^{\prime }\left\{\mathrm{prox}\left(x\rho \right)\left(u\right)\right\}}.$$

Hence we have

$$\underset{x}{\sup}|\frac{\partial }{\partial x}\mathrm{prox}\left(x\rho \right)\left(u\right)|\leq {∥\psi ∥}_{\infty }.$$

We conclude that

$$\forall u,\;|\psi^{\prime }\left\{\mathrm{prox}\left(x_{i}\rho \right)\left(u\right)\right\}-\psi^{\prime }\left\{\mathrm{prox}\left(y_{i}\rho \right)\left(u\right)\right\}|\leq \{L\left(n\right){∥\psi ∥}_{\infty }|x_{i}-y_{i}\left|\right\}\wedge {2∥\psi ∥}_{\infty }.$$

We therefore have, for x∨y≤B and x,y≥0,

$$\forall u,\underset{y:|x-y|\leq \eta }{\sup}|h_{u}^{\left(i\right)}\left(x\right)-h_{u}^{\left(i\right)}\left(y\right)|\leq \lambda_{i}^{2}\eta \psi^{\prime }\left\{\mathrm{prox}\left(x\lambda_{i}^{2}\rho \right)\left(u\right)\right\}+B\lambda_{i}^{4}L\left(n\right){∥\psi ∥}_{\infty }\eta .$$

Thus, for x,y≥0,

$$\forall u,\underset{(x,y):|x-y|\leq \eta ,x\vee y\leq B}{\sup}|h_{u}^{\left(i\right)}\left(x\right)-h_{u}^{\left(i\right)}\left(y\right)|\leq \lambda_{i}^{2}\eta ∥\psi^{\prime }{∥}_{\infty }+B\lambda_{i}^{4}L\left(n\right){∥\psi ∥}_{\infty }\eta .$$

Since the right-hand side does not depend on *u*, we have

$$\underset{u}{\sup}\underset{(x,y):|x-y|\leq \eta ,x\vee y\leq B}{\sup}|h_{u}^{\left(i\right)}\left(x\right)-h_{u}^{\left(i\right)}\left(y\right)|\leq \lambda_{i}^{2}\eta ∥\psi^{\prime }{∥}_{\infty }+B\lambda_{i}^{4}L\left(n\right){∥\psi ∥}_{\infty }\eta .$$

Naturally, $g_{n}\left(x\right)$ can be written as

$$g_{n}\left(x\right)=\frac{1}{n}\sum_{i=1}^{n}h_{{\tilde{r}}_{i,(i)}}^{\left(i\right)}\left(x\right).$$

Therefore, for any x,y≥0,

$$|g_{n}\left(x\right)-g_{n}\left(y\right)|\leq \frac{1}{n}\sum_{i=1}^{n}\left|h_{{\tilde{r}}_{i,(i)}}^{\left(i\right)}\left(x\right)-h_{{\tilde{r}}_{i,(i)}}^{\left(i\right)}\left(y\right)\right|.$$

The bound we have obtained above on $\sup_{u}\left|h_{u}^{\left(i\right)}\left(x\right)-h_{u}^{\left(i\right)}\left(y\right)\right|$ when *x* and *y* are sufficiently close to one another can now be used. This shows that for *x* given, if x,y≥0, |x−y|≤η, and x∨y≤B, we have

$$\underset{(x,y):|x-y|\leq \eta ,0\leq x\leq B,0\leq y\leq B}{\sup}|g_{n}\left(x\right)-g_{n}\left(y\right)|\leq \eta \frac{1}{n}\sum_{i=1}^{n}(\lambda_{i}^{2}∥\psi^{\prime }{∥}_{\infty }+BL\left(n\right)\lambda_{i}^{4}{∥\psi ∥}_{\infty }).$$

Under Assumptions **P2** and **F3–F4**, all the terms on the right-hand side are bounded in $L_{1}$. We can now take expectations and obtain the result in $L_{1}$. □

**Lemma A14.** 

*Let us call $G_{n}\left(x\right)=E\left(g_{n}\left(x\right)\right)$. Let B>0 be given. For any given $x_{0}\leq B$,*

$$g_{n}\left(x_{0}\right)-G_{n}\left(x_{0}\right)=o_{L_{2}}\left(1\right).$$

*Under Assumptions* ***O1–O7*** *,* ***P1–P4*** *and* ***F1–F5*** *, we have*

$$E^{*}\left(\underset{0\leq x\leq B}{\sup}\left|g_{n}\left(x\right)-G_{n}\left(x\right)\right|\right)\to 0.$$

**Proof.** Under Assumptions **F1** and **F5**, we can divide the index set {1,…,n} into finite *K* subsets $A_{1},\ldots ,A_{K}$, in which ${\left(x_{i},\epsilon_{i}\right)}_{i\in A_{j}}$ play a symmetric role. Hence, $\mathrm{var}(g_{n}\left(x_{0}\right))$ can be expressed as a sum of variances and covariances of finitely many functions of finitely many random variables $\left(\lambda_{i},{\tilde{r}}_{i,(i)}\right)$: for those random variables, we just need to pick a representative in each subset ${\left\{A_{j}\right\}}_{j=1}^{K}$. Since $\psi^{\prime }$ is Lipschitz, $g_{n}$ is an average of bounded continuous functions of the random variables of interest to us. Asymptotic pairwise independence of $\left(\lambda_{i},{\tilde{r}}_{i,(i)}\right)$’s implies that

$$\mathrm{var}\left(g_{n}\left(x_{0}\right)\right)=o\left(1\right).$$

and therefore gives the first result. Now we pick ϵ>0. By the stochastic equicontinuity of $g_{n}$ and the bound in (A26), we can find $x_{1},\ldots ,x_{K}$, independent of *n*, such that for all x∈[0,B], there exists *l* such that when *n* is large enough,

$$E(|g_{n}\left(x\right)-g_{n}\left(x_{l}\right)|)\leq \epsilon .$$

Notice that

$$|g_{n}\left(x\right)-G_{n}\left(x\right)|\leq |g_{n}\left(x\right)-g_{n}\left(x_{l}\right)\left|+\right|g_{n}\left(x_{l}\right)-G_{n}\left(x_{l}\right)\left|+\right|G_{n}\left(x_{l}\right)-G_{n}\left(x\right)|.$$

We immediately get

$$E^{*}\left(\underset{0\leq x\leq B}{\sup}\left|g_{n}\left(x\right)-G_{n}\left(x\right)\right|\right)\leq 2\epsilon +E\left(\underset{1\leq l\leq K}{\sup}\left|g_{n}\left(x_{l}\right)-G_{n}\left(x_{l}\right)\right|\right).$$

Because *K* is finite, the fact that for all *l*, $g_{n}\left(x_{l}\right)-G_{n}\left(x_{l}\right)=o_{L_{2}}\left(1\right)$ implies that $\sup_{1\leq l\leq K}\left|g_{n}\left(x_{l}\right)-G_{n}\left(x_{l}\right)\right|=o_{L_{2}}\left(1\right)$. In particular, if *n* is sufficiently large, we have

$$E\left(\underset{1\leq l\leq K}{\sup}\left|g_{n}\left(x_{l}\right)-G_{n}\left(x_{l}\right)\right|\right)\leq \epsilon .$$

This gives the result. □

**Lemma A15.** 
*Assume* ***O1–O7*** *,* ***P1–P4*** *and* ***F1–F5*** *. Recall that $c_{\tau }=n^{-1}\mathrm{tr}\left\{{\left(S+\tau I_{p}\right)}^{-1}\right\}$. Call as before*

$$g_{n}\left(x\right)=\frac{1}{n}\sum_{i=1}^{n}\frac{1}{1+x\lambda_{i}^{2}\psi^{\prime }\left\{\mathrm{prox}\left(x\lambda_{i}^{2}\rho \right)\left({\tilde{r}}_{i,(i)}\right)\right\}}.$$

*Then $c_{\tau }$ is a near solution of*

$$\frac{p}{n}-\tau x-1+g_{n}\left(x\right)=0,$$

*i.e., $p/n-\tau c_{\tau }-1+g_{n}\left(c_{\tau }\right)=o_{L_{k}}\left(1\right)$, when 3α−1/2<0.*
*Asymptotically, near solution of*

$$\delta_{n}\left(x\right)≜\frac{p}{n}-\tau x-1+g_{n}\left(x\right)=0,$$

*are close to solutions of*

$$\Delta_{n}\left(x\right)=\frac{p}{n}-\tau x-1+E\left\{g_{n}\left(x\right)\right\}=0.$$

*More precisely, call $T_{n,\epsilon }=\left\{x:\right|\Delta_{n}\left(x\right)\left|\leq \epsilon \right\}$. We note that $T_{n,\epsilon }\subseteq \left(0,p/\left(n\tau \right)+\epsilon /\tau \right)$. For any given ϵ, as n→∞, near solutions of $\delta_{n}\left(x\right)=0$ belong to $T_{n,\epsilon }$ with high probability.*
*Our assumptions concerning the possible distributions of $\epsilon_{i}$’s, specifically Assumption* ***F1*** *, imply that as n→∞, there is a unique solution to $\Delta_{n}\left(x\right)=0$.*
*Hence $c_{\tau }$ is asymptotically deterministic.*

**Proof.** Note that $g_{n}\left(x\right)\leq 1$. Let $\delta_{n}\left(x\right)$ be the function

$$\delta_{n}\left(x\right)=\frac{p}{n}-\tau x-1+g_{n}\left(x\right),$$

and $\Delta_{n}\left(x\right)=E\left\{\delta_{n}\left(x\right)\right\}$. Call $x_{n}$ a solution of $\delta_{n}\left(x_{n}\right)=0$ and $x_{n,0}$ a solution of $\Delta_{n}\left(x_{n,0}\right)=0$. Since $0\leq g_{n}\left(x\right)\leq 1$, we have $x_{n}\leq p/\left(n\tau \right)$, for otherwise, $\delta_{n}\left(x\right)<0$. The same arguments shows that if x>(p/n+ϵ)/τ, then $\delta_{n}\left(x\right)<-\epsilon$ and $x\notin T_{n,\epsilon }$. Similarly, near solutions of $\delta_{n}\left(x\right)=0$ must be less or equal to (p/n+ϵ)/τ.

- **Proof of the fact that $c_{\tau }$ is such that $\delta_{n}\left(c_{\tau }\right)=o\left(1\right)$**

Recall that in the notation of Lemma A8, we have

$$\frac{p-1}{n}-\tau c_{\tau ,p}=\frac{1}{n}\mathrm{tr}\left(I_{n}-M\right).$$

According to (A20),

$$\frac{1}{n}\mathrm{tr}\left(I_{n}-M\right)=1-\frac{1}{n}\sum_{i=1}^{n}\frac{1}{1+\psi^{\prime }\left(r_{i,[p]}\right)\frac{1}{n}v_{i}^{⊤}{\left(S_{p}\left(i\right)+\tau I_{p}\right)}^{-1}v_{i}}.$$

According to Lemmas A8–A10, we have

$$\underset{i}{\sup}|\frac{1}{n}v_{i}^{⊤}{\left(S_{p}\left(i\right)+\tau I_{p}\right)}^{-1}v_{i}-\lambda_{i}^{2}c_{\tau ,p}|=O_{L_{k}}\left(\frac{\mathrm{polyLog}(n)}{n^{1/2-2\alpha }}\right).$$

When x≥0 and y≥0, |1/(1+x)−1/(1+y)|≤|x−y|∧1. Hence, we have

$$\begin{array}{cc} \; & \left|\frac{1}{n}\sum_{i=1}^{n}\frac{1}{1+\psi^{\prime }\left(r_{i,[p]}\right)\frac{1}{n}v_{i}^{⊤}{\left(S_{p}\left(i\right)+\tau I_{p}\right)}^{-1}v_{i}}-\frac{1}{n}\sum_{i=1}^{n}\frac{1}{1+\psi^{\prime }\left(r_{i,[p]}\right)\lambda_{i}^{2}c_{\tau ,p}}\right|\\ \; & \;\leq \underset{1\leq i\leq n}{\sup}|\frac{1}{n}v_{i}^{⊤}{\left(S_{p}\left(i\right)+\tau I_{p}\right)}^{-1}v_{i}-\lambda_{i}^{2}c_{\tau ,p}|{∥\psi^{\prime }∥}_{\infty }. \end{array}$$

We conclude that

$$p/n-\tau c_{\tau ,p}-1+\frac{1}{n}\sum_{i=1}^{n}\frac{1}{1+\lambda_{i}^{2}c_{\tau ,p}\psi^{\prime }\left(r_{i,[p]}\right)}=O_{L_{k}}\left(n^{-1/2+2\alpha }\mathrm{polyLog}\left(n\right)\right).$$

Exactly the same computations can be performed for $c_{\tau }$. We have established that

$$p/n-\tau c_{\tau }-1+\frac{1}{n}\sum_{i=1}^{n}\frac{1}{1+\lambda_{i}^{2}c_{\tau }\psi^{\prime }\left(R_{i}\right)}=O_{L_{k}}\left(n^{-1/2+2\alpha }\mathrm{polyLog}\left(n\right)\right).$$

Now we have seen in Theorem A1 that

$$\underset{i}{\sup}\left|R_{i}-\mathrm{prox}\left(c_{i}\rho \right)\left({\tilde{r}}_{i,(i)}\right)\right|=O_{L_{k}}\left(n^{-1/2+\alpha }\mathrm{polyLog}\left(n\right)\right).$$

By the assumptions on $\psi^{\prime }$, this implies that

$$\underset{i}{\sup}\left|\psi^{\prime }\left(R_{i}\right)-\psi^{\prime }\left\{\mathrm{prox}\left(c_{i}\rho \right)\left({\tilde{r}}_{i,(i)}\right)\right\}\right|=O_{L_{k}}\left(n^{-1/2+\alpha }\mathrm{polyLog}\left(n\right)\right).$$

We have furthermore noted that $\sup_{i}\left|c_{i}-\lambda_{i}^{2}c_{\tau }\right|=O_{L_{k}}\left(n^{-1/2+2\alpha }\mathrm{polyLog}\left(n\right)\right)$ in Corollary A1. Using Lemma A11, we can write

$$|\mathrm{prox}\left(c_{i}\rho \right)\left({\tilde{r}}_{i,(i)}\right)-\mathrm{prox}\left(\lambda_{i}^{2}c_{\tau }\rho \right)\left({\tilde{r}}_{i,(i)}\right){|\leq ∥\psi ∥}_{\infty }\left|c_{i}-\lambda_{i}^{2}c_{\tau }\right|$$

and hence

$$|\psi^{\prime }\left\{\mathrm{prox}\left(c_{i}\rho \right)\left({\tilde{r}}_{i,(i)}\right)\right\}-\psi^{\prime }\left\{\mathrm{prox}\left(\lambda_{i}^{2}c_{\tau }\rho \right)\left({\tilde{r}}_{i,(i)}\right)\right\}|=O_{L_{k}}{(∥\psi ∥}_{\infty }n^{-1/2+3\alpha }\mathrm{polyLog}\left(n\right)).$$

Gathering all these results, we have

$$|\psi^{\prime }\left(R_{i}\right)-\psi^{\prime }\left(\mathrm{prox}\left(\lambda_{i}^{2}c_{\tau }\rho \right)\left({\tilde{r}}_{i,(i)}\right)\right)|=O_{L_{k}}{\{(∥\psi ∥}_{\infty }+1)n^{-1/2+3\alpha }\mathrm{polyLog}\left(n\right)\}.$$

So we have shown that $\delta_{n}\left(c_{\tau }\right)=O_{L_{k}}\left(n^{-1/2+3\alpha }\mathrm{polyLog}\left(n\right)\right)$.

- **Final details**

By Lemma A14, we have $\delta_{n}\left(x\right)-\Delta_{n}\left(x\right)=o_{p}\left(1\right)$ for any given *x*. In our case, using the notation of this lemma, B=p/(nτ)+η/τ, for η>0 given. This implies that for any given ϵ>0,

$$\underset{x\in (0,p/(n\tau )+\eta /\tau )}{\sup}\left|\delta_{n}\left(x\right)-\Delta_{n}\left(x\right)\right|<\epsilon ,$$

with high probability when *n* is large enough. Therefore, for any ϵ>0, if $x_{n}$ is a solution to $\delta_{n}\left(x\right)=0$,

$$|\Delta_{n}\left(x_{n}\right)|<\epsilon \;\mathrm{with}\;\mathrm{high}\;\mathrm{probability}.$$

This means that $x_{n}\in T_{n,\epsilon }$ with high probability. The same reasoning applies for near solution of $\delta_{n}\left(x\right)=0$, which must belong to $T_{n,\epsilon }$ as n→∞ with high probability for any given ϵ>0. Note that $T_{n,\epsilon }$ is compact because it is bounded and closed, using the fact that $G_{n}=E\left(g_{n}\right)$ is continuous. If $T_{n,0}$ were reduced to a single point, we would have established the asymptotically deterministic character of $c_{\tau }$. Given our work on the asymptotic behavior of ${\tilde{r}}_{i,(i)}$ and our assumptions on $\epsilon_{i}$’s, we see that Lemma 3.39 from El Karoui [21] applies to $\lim_{n\to \infty }\Delta_{n}\left(x\right)$ under Assumption **F1**. Therefore, $T_{n,0}$ is reduced to a single point as n→∞ and $c_{\tau }$ is asymptotically deterministic. □

**Proof of Theorem 1.** Notice that

$$\frac{\partial }{\partial t}\mathrm{prox}\left(c\rho \right)\left(t\right)=\mathrm{prox}{\left(c\rho \right)}^{\prime }\left(t\right)=\frac{1}{1+c\psi^{\prime }\left(\mathrm{prox}\left(c\rho \right)\left(t\right)\right)}.$$

Therefore, $\Delta_{n}$ can be written as

$$\Delta_{n}\left(x\right)=\frac{p}{n}-\tau x-1+\frac{1}{n}\sum_{i=1}^{n}E\left\{{\mathrm{prox}}^{\prime }\left(x\lambda_{i}^{2}\rho \right)\left({\tilde{r}}_{i,(i)}\right)\right\}.$$

Hence, the limiting root of $\Delta_{n}\left(x\right)=0$ satisfies the first fixed-point equation in Theorem 1. Since Lemma A15 shows that $c_{\tau }$ is asymptotically arbitrarily close to this root, the first equation follows. The second equation comes from (A24). Theorem 1 is proved, with $c_{\rho }\left(\kappa \right)$ being the limit of $c_{\tau }$. □
